# Thermally Driven Supramolecular Chirality Evolution in Low‐Bandgap Fused‐Ring Conjugated Molecules for High‐Performance NIR Circularly Polarized Light Detection

**DOI:** 10.1002/advs.76299

**Published:** 2026-06-28

**Authors:** Jaeyong Ahn, Kwangmin Kim, Sangwook Lee, Seul Lee, SungHyun Hur, BongSoo Kim, Joon Hak Oh

**Affiliations:** ^1^ School of Chemical and Biological Engineering Institute of Chemical Processes Seoul National University Seoul Republic of Korea; ^2^ Department of Chemical Engineering Stanford University Stanford California USA; ^3^ Department of Chemistry Ulsan National Institute of Science and Technology (UNIST) Ulsan Republic of Korea; ^4^ Graduate School of Semiconductor Materials and Device Engineering Ulsan National Institute of Science and Technology (UNIST) Ulsan Republic of Korea; ^5^ Graduate School of Carbon Neutrality Ulsan National Institute of Science and Technology (UNIST) Ulsan Republic of Korea

**Keywords:** circular polarization, optoelectronics, organic field‐effect transistor, organic semiconductor, photodetector, supramolecular chirality, vertical channel

## Abstract

Near‐infrared (NIR) circularly polarized light (CPL) photodetection is of great importance due to its broad application potential in bioimaging, wearable healthcare, optical communication, and advanced optoelectronic systems. In this study, a supramolecular chirality evolution strategy in chiral low‐bandgap fused‐ring conjugated molecules (LFCs) is presented for high‐performance NIR CPL photodetection using Schottky barrier vertical organic field‐effect transistors (SB‐VOFETs). Halogen substitution combined with thermal annealing drives inversion and amplification of supramolecular chirality in enantiopure LFC thin films. F‐substituted LFCs exhibit progressive domain growth and hierarchical ordering with increasing annealing temperature, whereas Cl‐substituted LFCs show limited structural evolution above 150°C. These distinct crystallization behaviors directly correlate with chiroptical responses, with F‐substituted LFCs achieving a maximum absorption dissymmetry factor (|*g*_abs_|) of ∼0.1. When integrated into SB‐VOFETs, the optimized chiral films enable highly efficient NIR CPL photodetection, delivering a photocurrent dissymmetry factor (|*g*_ph_|) of ∼0.1, a specific detectivity of 4.9 × 10^1^ ^1^ Jones, an external quantum efficiency exceeding 900%, and a fast response time of ∼600 µs at 850 nm. These metrics represent the highest performance reported for NIR CPL. This study provides design guidelines for advancing high‐performance chiral optoelectronic devices through the synergistic integration of atomic substitution, thermal annealing, and device architecture engineering.

## Graphical abstract

A supramolecular chirality evolution strategy in chiral low‐bandgap fused‐ring conjugated molecules enables high‐performance near‐infrared (NIR) circularly polarized light photodetection. Halogen substitution and thermal annealing induce chiral amplification and hierarchical ordering. Integrated into Schottky barrier vertical transistors, the optimized films deliver record‐high photocurrent dissymmetry, outstanding detectivity, and fast response in the NIR region, demonstrating the potential of precisely engineered chiral optoelectronic systems.

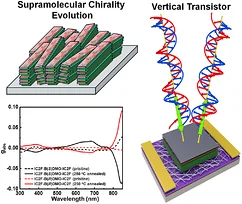

## 1 Introduction

Light carries various forms of optical information, including intensity, wavelength, and polarization during its propagation. Among the different polarization states, circular polarization is unique in that it carries spin angular momentum, setting it apart for specialized optical applications [1, 2]. Circularly polarized light (CPL) holds great potential for wide range of applications, including 3D displays, polarization imaging, drug sensing, optical communication, security systems, and even quantum computation [3, 4, 5, 6, 7]. In particular, CPL in the near‐infrared (NIR) region is highly attractive for biomedical imaging and autonomous driving applications due to its low‐loss characteristics in biological tissues and the atmosphere [8, 9, 10]. This has fueled intense interest in chiral materials, which interact asymmetrically with CPL and are key to enabling selective CPL detection and manipulation. However, achieving efficient NIR photodetection remains challenging due to the intrinsically weak NIR absorption and limited charge transport [11, 12].

The ability to manipulate and amplify chirality is crucial for controlling the interaction between CPL and matter. To modulate the chirality of materials, both thermodynamic and kinetic protocols have been developed. Thermodynamic control typically involves functional group modification to tailor electronic interactions and steric environments, thereby guiding supramolecular organization [13, 14]. As an example, previous research from our group demonstrated that bay‐substitution on chiral perylene diimide significantly influenced the supramolecular chirality of self‐assembled single crystals [13]. The kinetic protocols involve non‐equilibrium processes where external stimuli dynamically influence molecular self‐assembly pathways [15, 16, 17, 18]. For instance, temperature‐dependent supramolecular rearrangement can induce helical inversion in chiral materials, while solvent vapor annealing or light irradiation can guide molecular orientation through transient interactions or photoisomerization [19, 20, 21]. In helical‐shaped molecules, conformational interconversion becomes accessible once the applied energy surpasses the intrinsic *P*/*M* helicity inversion barrier [22, 23]. Such molecular‐level inversion can propagate to the mesoscale, altering the handedness of supramolecular assemblies and thereby modulating the overall chiroptical response. Therefore, an integrated understanding of these thermodynamic and kinetic strategies is pivotal for effectively modulating chirality of materials in advanced chiral optoelectronics. However, many aspects, including optimal molecular design, intermolecular interactions, self‐assembly kinetics, and external stimuli (thermal annealing, light, etc) that affect chirality, remain underexplored. A systematic investigation of how these parameters influence one another is necessary for effectively modulating and amplifying chiroptical properties toward real‐world chiral optoelectronic applications.

Numerous chiral compounds, including organic materials, organic–inorganic hybrid perovskites, and metal–organic complexes, have been explored [24, 25]. Among them, chiral organic semiconductors are particularly attractive owing to their tunability, processability, mechanical flexibility, and low weight [26, 27]. In recent years, low‐bandgap fused‐ring conjugated molecules (LFCs) featuring push–pull structures have been extensively investigated for high‐efficiency organic solar cells [28, 29, 30, 31]. In the context of chiral light detection, LFCs based on the Y6 molecular framework are especially promising due to their advantageous electronic and structural characteristics [29]. Y6 and its derivatives possess an acceptor–donor–acceptor–donor–acceptor (A–D–A–D–A) π‐conjugated backbone with high polarizability and strong intermolecular π–π stacking. These features lower the exciton binding energy, thereby facilitating efficient exciton dissociation and direct charge photogeneration upon light absorption [32, 33]. In addition, Y6 derivatives can adopt curved or twisted conformations as a result of steric interactions between alkyl chains attached to the nitrogens of conjugated backbone. Consequently, they can exhibit intrinsic chirality in the form of *P*‐ or *M*‐helical geometries [34]. Liu et al. reported chiral Y6 derivatives with chiral centers in the alkyl substituents and demonstrated that the point chirality of these substituents influences the supramolecular chirality of the stacked LFCs [9, 35]. Moreover, Li et al. reported that such supramolecular structure can produce spin polarization and reduce charge carrier recombination, resulting in extended charge carrier lifetime and high external quantum efficiency (EQE) [33]. Collectively, these features make Y6 derivatives bearing chiral centers excellent candidates for chiral organic photodetectors. Their intrinsic molecular packing and charge‐transport properties can synergistically enhance CPL detection upon the incorporation of chirality. Furthermore, Y6 derivatives exhibit strong absorption in the NIR region, and CPL photodetection in this spectral range remains rarely reported and generally shows limited performance [9, 36]. This limitation mainly arises from the twisted molecular structures of chiral materials, which hinder dense molecular packing and charge transport [10, 12]. Therefore, systematic investigations of chiral Y6 derivatives, considering both thermodynamic factors (e.g., chemical structural modifications) and kinetic factors (e.g., temperature‐ or solvent‐dependent processes), are highly warranted.

Conventional lateral transistors have intrinsic limitations due to the inherently long channel lengths required to prevent gate leakage, which in turn leads to high resistance and low current densities. Furthermore, their structure is incompatible with face‐on stacked semiconductors, resulting in inefficient charge transport. In contrast, vertical transistors, whose channel length is determined by the thickness of the active layer, offer great opportunities to overcome these limitations and can also serve as a common testbed for chiroptical properties [37, 38]. Among various types of vertical transistors, Schottky barrier‐vertical organic field‐effect transistors (SB‐VOFETs) utilize gate potential to control the height of Schottky barrier at the source‐organic semiconductor interface. Owing to their short channel length, they can achieve both high current density and the fast transition frequency. Moreover, this device configuration is well‐suited for face‐on‐stacked organic semiconductors such as Y6 derivatives, which favor vertical charge transport over lateral transport. Consequently, VOFET‐based photodetectors are an effective platform that can enhance photogenerated charge carrier collection and responsivity with chiral LFCs through short channel length and gate modulation.

Herein, we report the first demonstration of CPL photodetection based on chiral LFCs integrated into SB‐VOFET structures. By combining end group modification and thermal annealing, we induced distinct thermally driven evolutions of supramolecular chirality, resulting in tunable CPL selectivity. Furthermore, morphological and structural investigations of the annealed thin films allowed for identifying the optimal annealing temperature. The devices fabricated under these conditions exhibited a remarkable maximum photocurrent dissymmetry factor (|*g*_ph_|) of approximately 0.1 at 850 nm, a specific detectivity (*D* ^*^) of 4.9 × 10^1^ ^1^ Jones, and a response time of around 600 µs, which represent the highest performance reported for NIR CPL photodetectors. This study demonstrates a powerful and generalizable supramolecular chirality evolution strategy for supramolecular chirality engineering and its practical application in chiral optoelectronics.

## 2 Results and Discussion

### 2.1 Optical and Electrochemical, and Thermal Properties of Chiral LFCs

**FIGURE 1:**
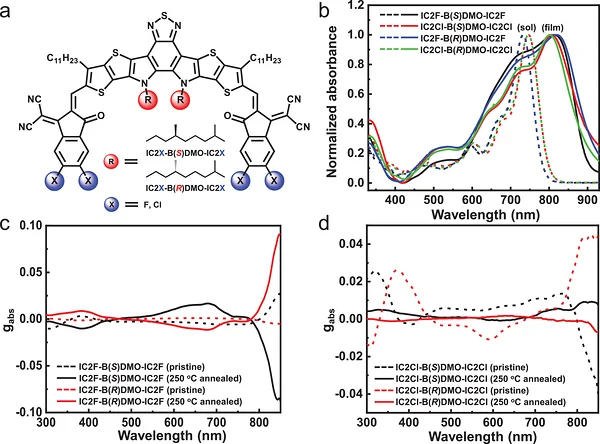
Molecular structures and chiroptical properties of chiral LFCs. (a) Chemical structures of chiral LFCs in this study. (b) UV–vis absorption spectra of chiral LFCs in solution (dotted lines) and thin‐film (solid lines) states. Circular dichroism spectra of pristine (dotted lines) and 250°C annealed (solid lines) thin films of (c) F‐substituted and (d) Cl‐substituted chiral LFCs.

The chemical structures of the synthesized chiral LFCs, IC2F‐B(*S*)DMO‐IC2F, IC2F‐B(*R*)DMO‐IC2F, IC2Cl‐B(*S*)DMO‐IC2Cl and IC2Cl‐B(*R*)DMO‐IC2Cl, composed of a 3,9‐diundecyl‐12,13‐dihydro‐[1,2,5]thiadiazolo[3,4‐*e*]thieno[2'',3'':4',5']thieno[2',3':4,5]pyrrolo‐[3,2‐g]thieno[2',3':4,5]thieno[3,2‐*b*]indole (TPTI) core and 1,1‐dicyanomethylene‐3‐indanone (IC) end‐capping group, are shown in Figure 1a. Enantiopure 3,7‐dimethyloctyl side chains are introduced on the pyrrole moieties, and fluorine (or chlorine) atoms are substituted on the end‐capping group. The detailed synthetic routes are provided in Supporting Information, together with spectroscopic data (Figures S1–S18). The synthesized molecules exhibited ultraviolet, visible, and near‐infrared absorption, with absorption peaks at 709 and 723 nm in solution for the F‐substituted LFCs and Cl‐substituted LFCs, respectively (Figure 1b). The Cl‐substituted LFCs have a wider light absorption because stronger intermolecular packing and lower optical bandgap than F‐substituted one [39]. In the film state, all LFCs exhibited a clear red‐shift, attributable to the intermolecular interactions between the molecules through intermolecular aggregation. Notably, the F‐substituted LFC films displayed a more pronounced redshift than the Cl‐substituted counterparts, suggesting stronger intermolecular coupling in the slip‐stacked J‐type aggregation of the F‐substituted films [40].

Cyclic voltammetry measurements were taken for all LFCs (Figure S19). The IC2F‐B(*S*)DMO‐IC2F and IC2F‐B(*R*)DMO‐IC2F have the highest occupied molecular orbital (HOMO) / the lowest unoccupied molecular orbital (LUMO) energy levels of −5.71/−3.88 and −5.70/−3.89 eV, respectively. Meanwhile, the IC2Cl‐B(*S*)DMO‐IC2Cl and IC2Cl‐B(*R*)DMO‐IC2Cl showed deeper‐lying levels with HOMO/LUMO energy levels of −5.76/−4.05 and −5.78/ −4.05 eV, respectively.

To investigate the chiroptical properties of the LFCs, circular dichroism (CD) spectra were recorded in both solution and thin‐film states. Enantiopure LFCs did not exhibit any detectable CD signals in solution (Figure S20a), presumably because the point chirality of the alkyl side chains alone is insufficient to induce a detectable chiroptical response at the molecular level. In contrast, thin films of IC2F‐B(*S*)DMO‐IC2F and IC2F‐B(*R*)DMO‐IC2F displayed pronounced CD signals (Figure 1c), indicating the emergence of supramolecular chirality in the solid state, potentially accompanied by intermolecular excitonic interactions [41]. Because the TPTI and IC groups adopt either *P*‐or *M*‐type helicity, the LFCs with these helical comformations stack to form long‐range asymmetric structures, thereby significantly amplifying the overall chiroptical response [8, 41]. To our surprise, the CD signals were clearly reversed upon thermal annealing at 180°C and increased up to 250°C (Figure 1c and Figure S20b), indicating that high‐temperature annealing promotes reorganization of the helical stacks. The observed CD inversion may arise from thermally driven redistribution or reorganization of multiple chiral domains with distinct packing arrangements [42, 43]. Remarkably, the annealed IC2F‐B(*S*)DMO‐IC2F thin films achieved a maximum dissymmetry factor (*g*_abs_) of around 0.1 at 850 nm. This phenomenon is also observed in Cl‐substituted chiral LFCs, which showed a transition in the CD spectra after annealed above 150°C (Figure 1d and Figure S20c). The annealing temperature dependent ellipticity is summarized in Figure S20d. A notable point is that the LFCs containing IC2F exhibit an increase in |*g*_abs_| from 0.03 to 0.1 after 250°C annealing, whereas LFCs with IC2Cl show a decrease in |*g*_abs_| from 0.04 to 0.01 after 250°C annealing (Figure 1c,d). Moreover, we note that the enantiopure Cl‐substituted LFCs exhibited nearly mirror‐image CD spectra compared to the F‐substituted LFCs (Figure S21a). This observation suggests that subtle end‐group substitution, (i.e. IC2F vs. IC2Cl), can alter intermolecular interactions and packing preferences, which may shift the helical bias of supramolecular crystal growth in opposite directions [19, 39]. The origin of this difference can be attributed to the distinct steric and electronic characteristics of the F and Cl substituents. Chlorine is larger and more polarizable than fluorine, which can lead to different end‐group contact geometries, steric constraints, and halogen‐related intermolecular interactions. These differences may influence the relative stability and kinetic accessibility of polymorphic packing motifs during thermal annealing, resulting in different temperature‐dependent CD responses. Both F‐ and Cl‐substituted chiral LFCs exhibited similar spectra under forward and backward light incidence to the films, confirming their intrinsic chirality and ruling out any linear dichroism/linear birefringence effects (Figure S21b,c) [44, 45, 46].

**FIGURE 2:**
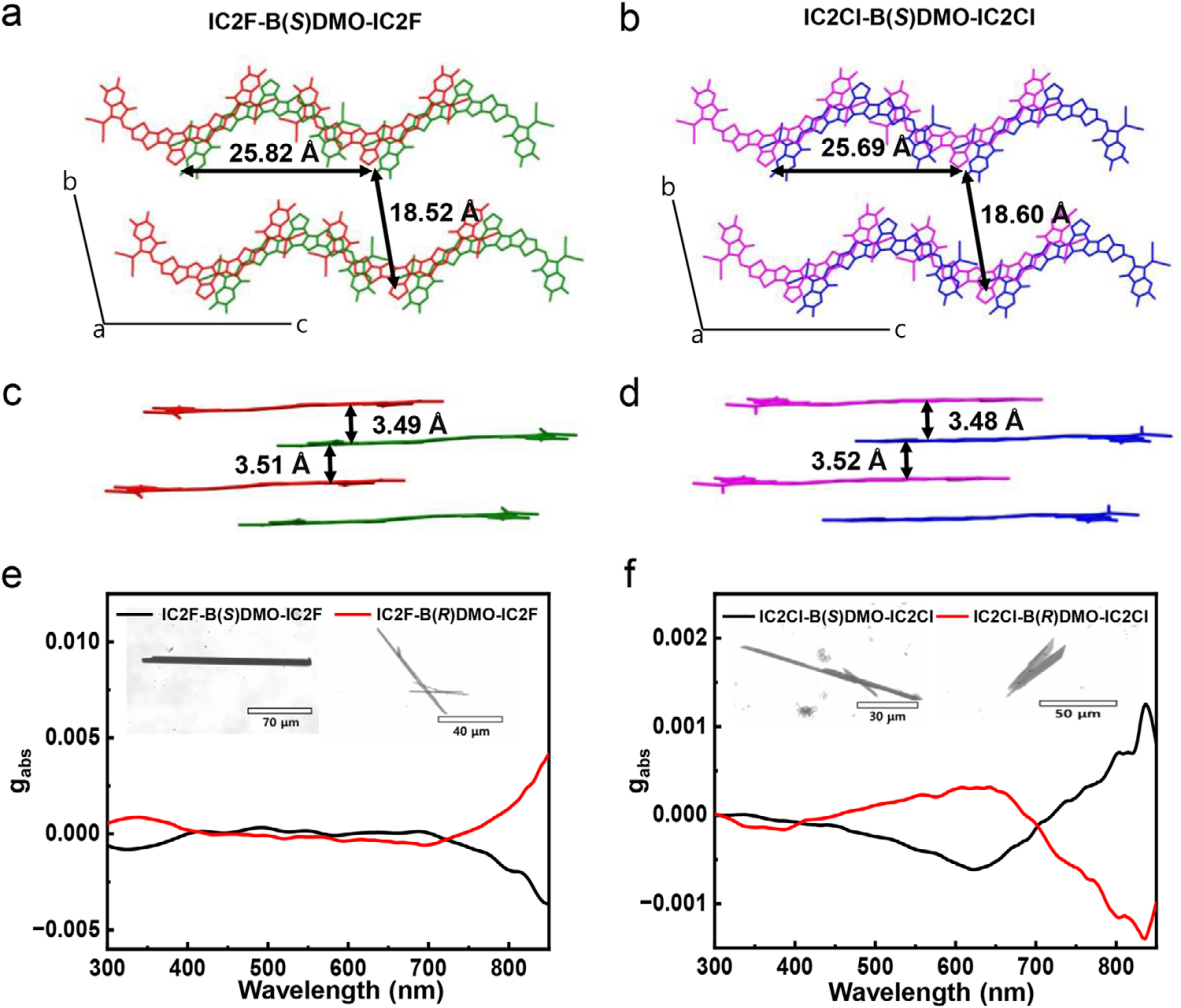
Structural analysis and resulting chiroptical properties of chiral LFC single crystals. Molecular packing of (a) IC2F‐B(*S*)DMO‐IC2F and (b) IC2Cl‐B(*S*)DMO‐IC2Cl viewing along the a‐axis. π–π stacking of (c) IC2F‐B(*S*)DMO‐IC2F and (d) IC2Cl‐B(*S*)DMO‐IC2Cl (for the clarity, all the alkyl chains are omitted). Circular dichroism spectra and optical microscope images of single crystal dispersions in methanol for (e) F‐substituted and (f) Cl‐substituted chiral LFCs.

To gain insight into the molecular packing and supramolecular organization of chiral LFCs, single crystals were grown by a slow solvent diffusion method. Single‐crystal X‐ray diffraction analysis was conducted on chiral LFC single crystals. The stacking information is presented in Figure 2a–d and Table S1. As chiral LFCs shared the same backbone structure with Y6, their single‐crystal structures exhibit banana‐shaped geometric features. The chiral LFCs adopt a quasi‐2D supramolecular packing motif, consistent with the crystal structure previously reported by the Liu group [41]. The intermolecular distances along the *b* and *c* axes of the unit cell are approximately 18.5 and 25.7 Å, respectively, and the π–π stacking distance is around 3.5 Å. Owing to their molecular conformations and supramolecular organizations, the chiral LFCs produced needle‐like crystals, as shown by optical microscopy images (Figure S22). To correlate the molecular packing with the chiroptical response, CD measurements were further analyzed. Interestingly, both chiral LFC single crystals with IC2F and IC2Cl end groups exhibited the same CD spectral sign as the 250°C annealed films (Figure 2e,f). The inverted CD spectral profile relative to that of the pristine film indicates that helicity inversion occurs during the crystallization process as a result of structural reorganization. Accordingly, the annealed films possess a high degree of crystallinity comparable to that of the single crystal.

**FIGURE 3:**
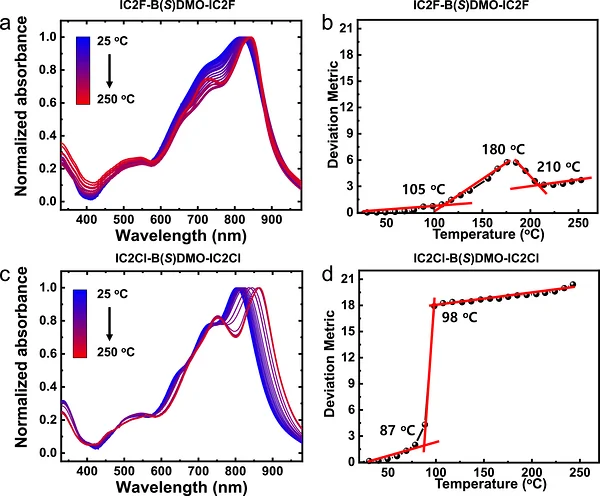
Temperature‐dependent aggregation behavior of chiral LFC thin films. (a) Normalized temperature‐dependent UV–vis absorption spectra and (b) its deviation metric plot from 600 to 900 nm of IC2F‐B(*S*)DMO‐IC2F thin film. (c) Normalized temperature‐dependent UV–vis absorption spectra and (d) its deviation metric plot from 600 to 900 nm of IC2Cl‐B(*S*)DMO‐IC2Cl thin film.

Thermal properties of all chiral LFCs were examined by thermogravimetric analysis (TGA), differential scanning calorimetry (DSC), and UV–vis absorption deviation metric analysis. TGA revealed that these chiral LFCs remain thermally stable up to 280°C (Figure S23). In DSC analysis, endothermic peaks were observed at 183°C and 187°C for the *S*‐ and *R*‐chiral LFCs with the IC2F end group, respectively, while LFCs with the IC2Cl end group exhibited no noticeable thermal transitions (Figure S24). The UV–vis absorption deviation metric method revealed more detailed phase transitions (Figure 3). In the IC2F‐B(*S*)DMO‐IC2F film, the first transition occurs between 105°C and 180°C, consistent with the DSC data, followed by minimal change until a second transition initiates at 210°C. This behavior is likely associated with the CD signal inversion, i.e. different helical growth in the film state. In the IC2Cl‐B(*S*)DMO‐IC2Cl film, a transition begins at 87°C, after which the material remains stable with no further changes at higher temperatures. Similar temperature‐responsive phase transitions were observed for IC2F‐B(*R*)DMO‐IC2F and IC2Cl‐B(*R*)DMO‐IC2Cl (Figure S25). Thermal characterization thus reveals a non‐monotonic evolution in the degree of molecular phase transition, which is strongly dependent on the nature of the end group. These findings underscore the critical role of end‐group engineering in modulating the supramolecular structure and thermal behavior of chiral LFCs.

### 2.2 Morphological and Structural Characterization

**FIGURE 4:**
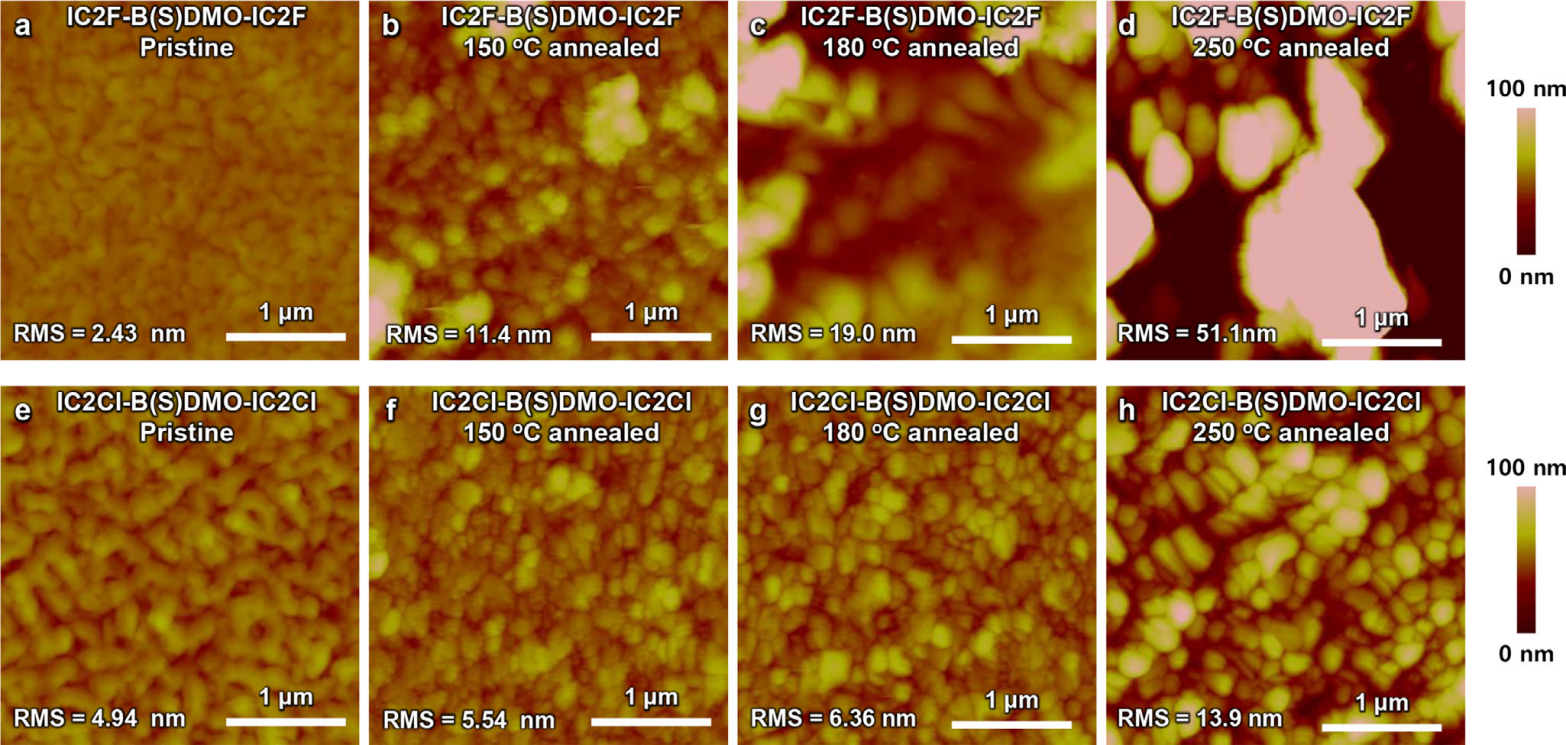
Annealing‐dependent surface morphology of chiral LFC thin films. AFM height images of (a–d) IC2F‐B(*S*)DMO‐IC2F and (e–h) IC2Cl‐B(*S*)DMO‐IC2Cl thin films at different annealing temperatures: (a,e) Pristine, (b,f) 150°C, (c,g) 180°C, and (d,h) 250°C.

To explore the thermal evolution of crystallinity and molecular packing in chiral LFCs, atomic force microscope (AFM) imaging was conducted on the LFCs films. The height images of LFCs films are shown in Figure 4 and the corresponding phase images are displayed in Figure S26. The pristine films of both IC2F‐B(*S*)DMO‐IC2F and IC2Cl‐B(*S*)DMO‐IC2Cl exhibit small domains, with root mean square roughness (*R*_rms_) values of 2.43 and 4.94 nm, respectively. The two LFCs exhibited domain growth upon thermal annealing. For the IC2F‐B(*S*)DMO‐IC2F film, the *R*_rms_ value increased to 11.4, 19.0, and 51.1 nm after annealing at 150°C, 180°C, and 250°C, respectively. These results indicate that molecular aggregation and crystallization are strongly promoted at elevated temperatures. For the IC2Cl‐B(*S*)DMO‐IC2Cl film, *R*_rms_ value increased with much lower change, reaching 5.54, 6.36, and 13.9 nm at 150°C, 180°C, and 250°C, respectively. The lower degree of *R*_rms_ change is consistent with the higher thermal stability of IC2Cl‐B(*S*)DMO‐IC2Cl.

**FIGURE 5:**
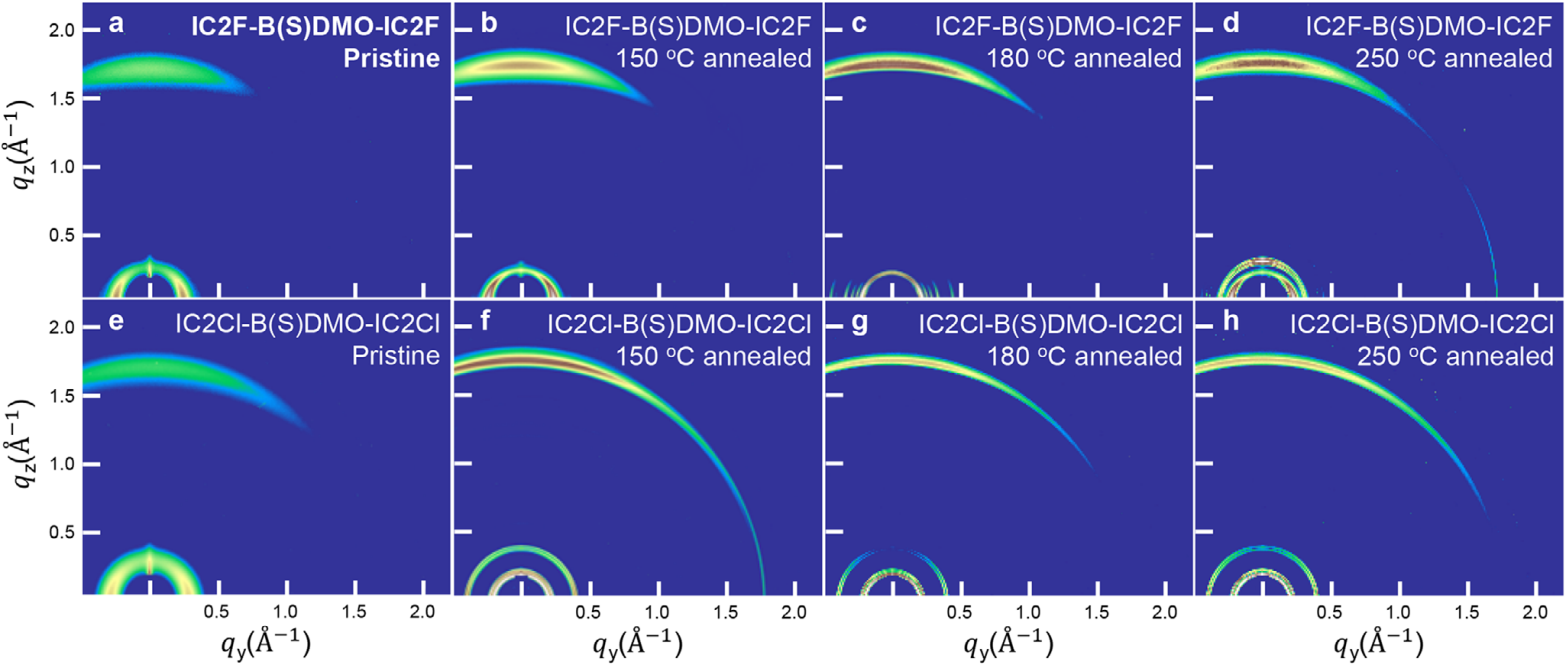
Annealing‐dependent structural analysis of chiral LFC thin films. GIWAXS images of (a–d) IC2F‐B(*S*)DMO‐IC2F and (e–h) IC2Cl‐B(*S*)DMO‐IC2Cl thin films at different annealing temperatures: (a,e) Pristine, (b,f) 150°C, (c,g) 180°C, and (d,h) 250°C.

**FIGURE 6:**
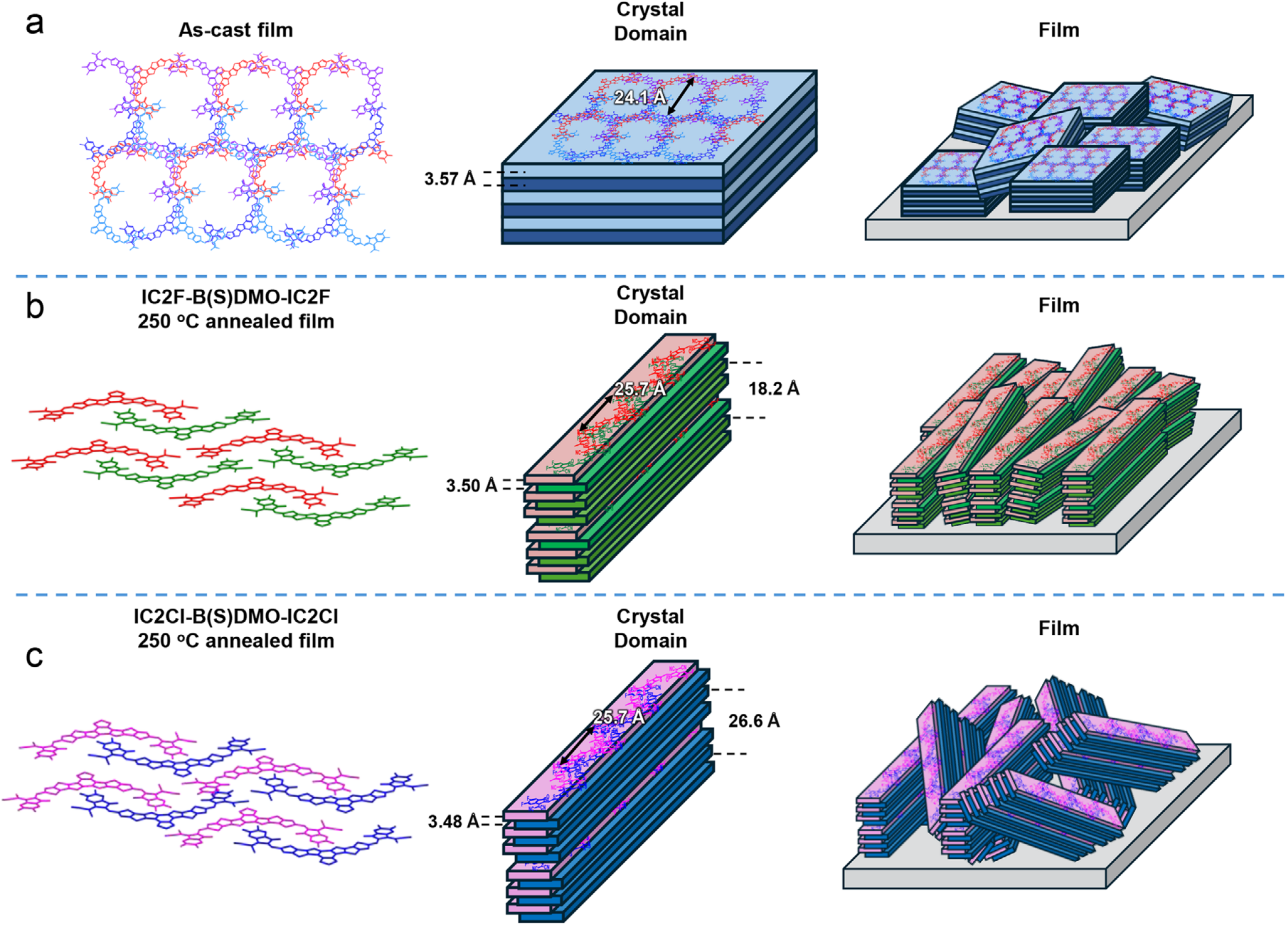
A schematic view of the molecular packing networks of chiral LFCs in the films. (a) pristine IC2F‐B(*S*)DMO‐IC2F film. (b) IC2F‐B(*S*)DMO‐IC2F film after thermal annealing at 250°C. (c) IC2Cl‐B(*S*)DMO‐IC2Cl film after thermal annealing at 250°C.

While AFM revealed the surface morphology and domain growth behavior, grazing incidence wide angle X‐ray scattering (GIWAXS) provided insights into the underlying molecular packing and crystallinity. Annealing‐dependent GIWAXS images of chiral LFC thin films are shown in Figure 5. Their corresponding line‐cut profiles and fittings are presented in Figures S27–S29, and the extracted key structural parameters are summarized in Table S2. In the pristine films, both IC2F‐B(*S*)DMO‐IC2F and IC2Cl‐B(*S*)DMO‐IC2Cl exhibited strong π–π stacking peaks in the out‐of‐plane (*q*_z_) direction at *q* = 1.75 Å^−1^ (*d* ≈ 3.57 Å), indicating that the molecules are aligned in a face‐on orientation relative to the substrate. In the in‐plane (*q*_y_) direction, lamellar stacking peaks were observed at *q* = 0.26 and 0.31 Å^−1^, respectively. These GIWAXS results, along with the schematic molecular packing diagram shown in Figure 6a, suggest that the crystal domain exhibited a 3D honeycomb stacking structure similar to the Y6 structure [47].

Upon thermal annealing, however, the two materials exhibited markedly different structural evolutions. In the IC2F‐B(*S*)DMO‐IC2F film annealed at 150°C, a reduced π–π stacking distance along with increased correlation length indicates the growth of well‐ordered crystalline domains. Upon annealing at 180°C, a strong π–π stacking peak appeared in the *q*_z_ direction at *q* = 1.79 Å^−1^ (*d* = 3.51 Å), accompanied by a markedly increased correlation length to beyond 11 nm. Notably, new diffraction features appeared at *q* ≈ 0.25 Å^−1^ along the *q*_z_ direction and in the range of *q* = 0.21–0.47 Å^−1^ along the *q*_y_ direction. This structural transformation indicates that thermal annealing induces a transition to the new structural motif, which in turn leads to the inversion of the chiroptical signal. After annealing at 250°C, the structural ordering of stacking domains became even more refined. The π–π stacking peak remained in the *q_z_* direction at *q* = 1.80 Å^−1^ (*d* = 3.50 Å), while strong lamellar‐like diffraction peaks were observed around *q* = 0.35 Å^−1^ (*d* ≈ 18 Å), with high intensity and a large correlation length, suggesting denser and more uniform reorganization of the stacking domains. In the *q*_y_ direction, a single dominant peak emerged at *q* = 0.25 Å^−1^ (*d* = 25.7 Å). These results suggest that the film annealed at 250°C adopts a quasi‐2D supramolecular packing motif similar to that of the single crystal, as illustrated in Figure 6b. This assignment is further supported by the agreement in key diffraction peak positions between the simulated powder X‐ray diffraction pattern derived from the single‐crystal structure and the GIWAXS profiles of the annealed thin film (Figure S30). The sign inversion observed in the CD spectra was attributed to a structural transition from the 3D honeycomb stacking structure in the pristine film to a quasi‐2D supramolecular packing structure upon annealing. Additionally, enhanced hierarchical ordering is consistent with the higher |*g*_abs_| value of the 250°C annealed thin films than the pristine film in the CD spectra.

IC2Cl‐B(*S*)DMO‐IC2Cl exhibited a much more limited structural response to thermal annealing. Upon thermal annealing above 150°C, the π–π stacking peak shifted to approximately *q* ≈ 1.80 Å^−1^ (*d* = 3.50 Å), and the correlation length markedly increased to 12 nm, suggesting the growth of stacking domains. In the *q*_y_ direction, new lamellar‐like stacking peaks emerged in the *q* ≈ 0.25 Å^−1^ (*d* ≈ 25.7 Å) region, accompanied by significantly enhanced intensity compared to the pristine film. Once this stacking structure formed at 150 °C, no notable changes were observed at 180 °C and 250 °C. These results suggest that the annealed IC2Cl‐B(*S*)DMO‐IC2Cl film also exhibits a quasi‐2D supramolecular packing structure and alignment were already saturated at 150 °C. The GIWAXS pattern of annealed IC2Cl‐B(*S*)DMO‐IC2Cl film exhibits a more isotropic shape compared to that of IC2F‐B(*S*)DMO‐IC2F. This suggests that the annealed stacking orientation of the annealed IC2Cl‐B(*S*)DMO‐IC2Cl films becomes more dispersed and partially randomized than that of the annealed IC2F‐B(*S*)DMO‐IC2F films (Figure 6c). These results demonstrate that IC2Cl‐based LFCs also observed sign inversion in the CD spectra upon annealing but exhibit lower CD intensities than the pristine film. The polarized optical microscopy images revealed that both annealed F‐ and Cl‐ substituted LFC films exhibited birefringent crystalline domains, confirming the presence of locally anisotropic ordered domains (Figure S31).

### 2.3 Vertical Transistor Based CPL Detectors

**FIGURE 7:**
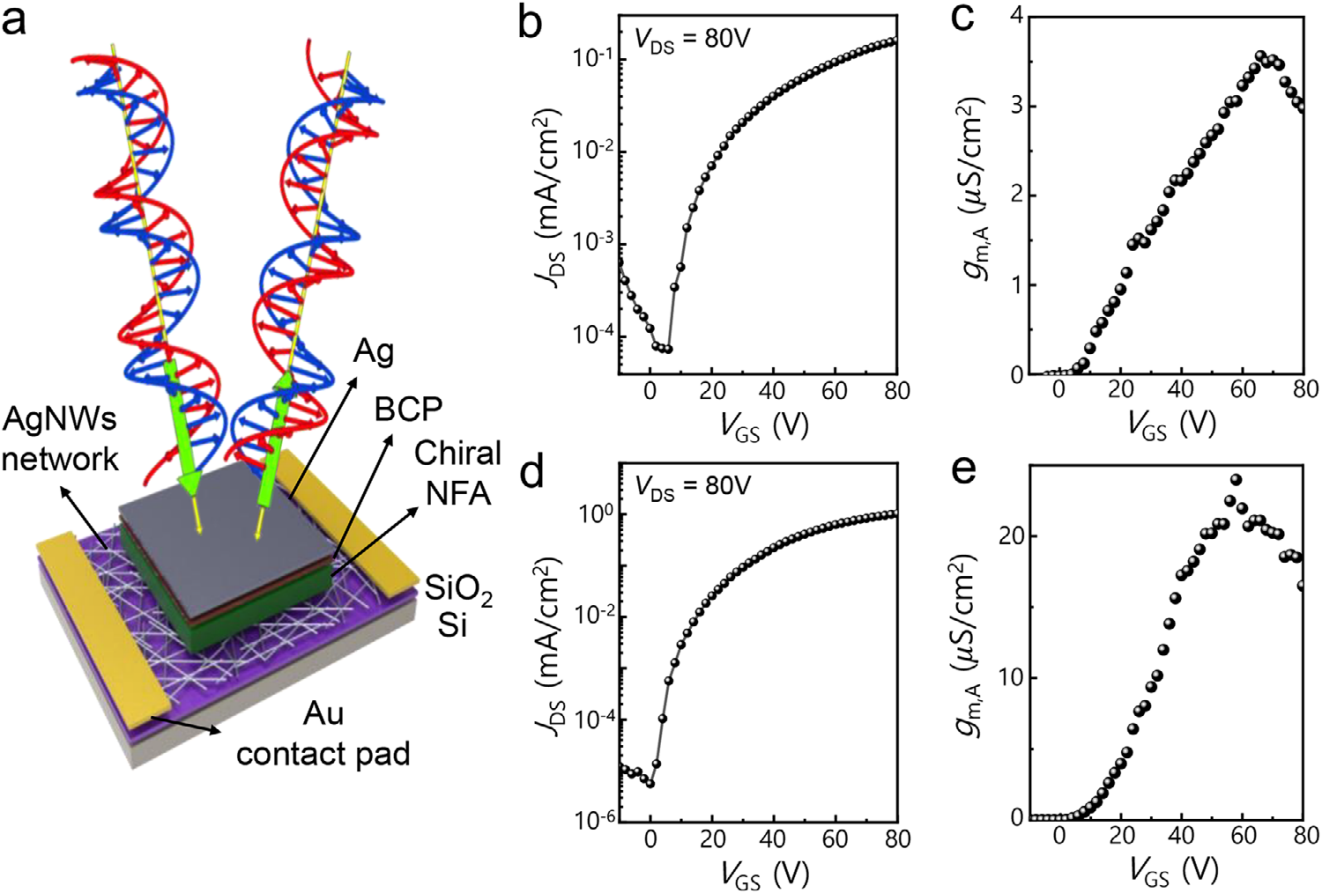
Electrical characteristics of SB‐VOFETs based on chiral LFC thin films. (a) Schematic illustration of fabricated chiral LFC‐based SB‐VOFETs for CPL detection. (b) Transfer characteristics of SB‐VOFETs based on IC2F‐B(*S*)DMO‐IC2F thin films annealed at 230°C under dark and vacuum conditions. (c) Corresponding specific transconductance values normalized by the unit area. (d) Transfer characteristics of SB‐VOFETs based on IC2Cl‐B(*S*)DMO‐IC2Cl thin films annealed at 230°C under dark and vacuum conditions. (e) Corresponding specific transconductance values normalized by the unit area.

To take advantage of the inherent vertical charge transport characteristics of chiral LFCs, we employed a vertical transistor configuration. SB‐VOFET structure was constructed by inserting chiral active layer between silver nanowire (AgNW) source and bathocuproine (BCP)/Ag drain (Si/SiO_2_/AgNW/chiral LFC/BCP/Ag). When the gate potential is applied, the height of the Schottky barrier between source electrode and semiconductor contact is modulated, affecting the charge carrier injection probability. Therefore, the density of the AgNW for source network needed to be carefully optimized to ensure the appropriate electric field penetration and charge accumulation in our SB‐VOFET configuration, thereby controlling the penetration of the gate field through source electrode network [48]. Thermal annealing at 230°C was applied to the chiral active layers for the stable transistor operation, as higher annealing temperatures led to degraded device stability. As a result, we demonstrated for the first time the vertical transistor configuration for CPL photodetection. A schematic illustration of chiral LFCs based SB‐VOFETs is presented in Figure 7a. Both IC2F‐B(*S*)DMO‐IC2F and IC2Cl‐B(*S*)DMO‐IC2Cl based SB‐VOFETs exhibited stable *n*‐type transistor operation (Figure 7b,c). The transfer characteristics in Figure 7b indicates that IC2F‐B(*S*)DMO‐IC2F‐based devices deliver current density exceeding 0.1 mA cm^−2^, with an I_on_/I_off_ ratio above 10^3^. The Cl‐substituted chiral LFC, IC2Cl‐B(*S*)DMO‐IC2Cl exhibited higher current densities over 1 mA cm^−2^, while displaying outstanding I_on_/I_off_ ratio above 10^5^. Consequently, the resulting area‐normalized transconductance was estimated to be 20 µS cm^−2^, which is higher than that of the F‐substituted LFC based VOFETs (Figure 7d,e). Notably, the materials possessing opposite chirality yielded similar characteristics, as shown in Figure S32. In addition, these devices demonstrated stable operation, as evidenced by output behavior across a wide range of gate voltages and negligible changes in drain current and threshold voltage during cyclic operation (Figures S32 and S33).

**FIGURE 8:**
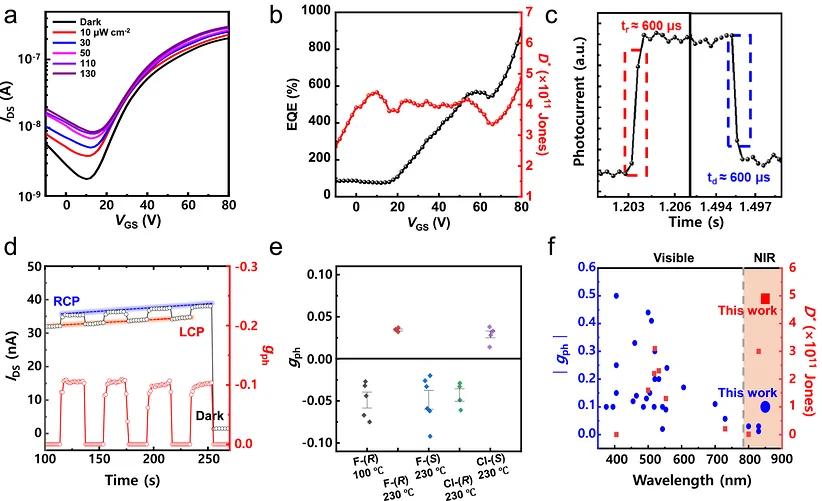
CPL photodetection characteristics of chiral LFC‐based SB‐VOFETs. (a) *I*_DS_–*V*_GS_ characteristics of SB‐VOFETs based on IC2F‐B(*S*)DMO‐IC2F thin films annealed at 230°C under monochromatic light illumination (λ = 850 nm) with varying intensities. (b) EQE and *D* ^*^ as a function of *V*_GS_ for the same devices (*λ* = 850 nm, *I* = 10 µW cm^−2^). (c) Real‐time photoswitching response used for rise and decay time measurements. (d) Time‐dependent CPL detection and corresponding *g*_ph_ values of 230°C annealed IC2F‐B(*S*)DMO‐IC2F based SB‐VOFETs. (e) Summary of *g*_ph_ values of SB‐VOFET based CPL photodetectors employing various chirality‐tuned chiral LFCs. (f) |*g*_ph_| (circle symbol) and D* (square symbol) plotted against wavelength from our work and previous reports. The values obtained from our work were indicated with larger symbols.

The fabricated chiral LFC‐based SB‐VOFETs were employed as NIR photodetectors, and their fundamental photodetection properties were investigated prior to assessing CPL sensitivity. IC2F‐B(*S*)DMO‐IC2F based SB‐VOFETs were illuminated with 850 nm monochromatic light. Under gradually increasing light intensities, IC2F‐B(*S*)DMO‐IC2F based SB‐VOFET exhibited an increase in drain current and a negative shift in their threshold voltage (Figure 8a). The extracted EQE and *D* ^*^ parameters depending on the gate voltages are shown in Figure 8b. When thermally annealed at 230°C, IC2F‐B(*S*)DMO‐IC2F yielded maximum EQE and *D* ^*^ values of 909% and 4.9 × 10^11^ Jones, under 10 µW cm^−2^ light irradiation. Benefiting from the gate field effect, the excellent charge transporting capability of the LFCs, and the unique device configuration matching with molecular stacking direction, our devices exhibited outstanding EQE compared to previously reported NIR CPL photodetectors [8, 9, 12, 36, 49]. The maximum values of these photodetection parameters at different illumination intensities are summarized in Figure S34. As the light intensity increased, photoresponsibility, EQE and *D* ^*^ values declined, mainly due to a shortened lifetime of photogenerated charge carriers resulting from saturated charge trap states at higher intensities [50, 51]. Dynamic photoresponses of chiral LFC‐based SB‐VOFETs were displayed in Figure 8c. The rise time (*t*_r_) was determined by the time it took the current to increase from 10% to 90% of its peak value once illumination was switched on, and the decay time (*t*_d_) corresponded to the time it took the current to drop from 90% to 10% of its peak value after illumination was switched off. Both the extracted rise and decay times were less than 600 µs, demonstrating a fast photoresponse suitable for high‐speed photoswitching applications. As shown in Figure S35, the devices exhibited stable photoswitching operation with multiple light on/off cycles within a few seconds.

Finally, the CPL detection capability of chiral LFC‐based SB‐VOFETs was examined. The *g*_ph_ is defined as follows:

(1) $$g_{\mathrm{ph}}=\frac{2(I_{L}-I_{R})}{I_{L}+I_{R}}$$

where *I*_L_ and *I*_R_ describe the photocurrent of the SB‐VOFETs under left‐ and right‐handed CPL irradiation. To investigate how CPL detection performance is influenced by chirality evolution of the thin films, we compared the devices subjected to different thermal annealing conditions as well as end‐group modifications. Since devices fabricated without thermal annealing exhibited low yield and poor reproducibility, thin films based on F‐substituted LFCs were annealed at 100°C to ensure consistent VOFET operation while maintaining their chiroptical properties. These films were further compared with those annealed at 230°C, which exhibited distinguishable chiroptical responses in CD measurements. The resulting SB‐VOFETs displayed clear real‐time photocurrent differences under left‐ and right‐handed CPL irradiation at 850 nm (Figures S36 and S37). The maximum |*g*_ph_| value reached 0.1 for IC2F‐B(*S*)DMO‐IC2F film based SB‐VOFETs (Figure 8d). Notably, CPL preference was reversed depending on annealing temperature and end‐group modification, consistent with the CD data. For example, IC2F‐B(*R*)DMO‐IC2F films annealed at 230°C favored left‐handed CPL, whereas those annealed at 100°C produced higher photocurrents under right‐handed CPL. The average *g*_ph_ value was −0.049 in SB‐VOFETs based on IC2F‐B(*R*)DMO‐IC2F annealed at 100°C, but reversed to 0.034 in devices annealed at 230°C along with the growth and rearrangement of crystalline domains, as displayed in Figure 8e. A similar reversal was observed when the end group was changed from fluorine to chlorine. SB‐VOFETs based on IC2Cl‐B(*R*)DMO‐IC2Cl films annealed at 230°C exhibited a preference for right‐handed CPL with an average *g*_ph_ value of −0.043. In principle, CPL selectivity devices may also be affected by processes beyond optical absorption, including chirality induced spin selectivity, device configuration, electric and magnetic fields, recombination location, and irradiation direction [26, 33, 52]. However, in our devices, the reversal of CPL preference upon changing the annealing temperature and halogen end group closely follows the corresponding reversal in the CD spectra. This correlation suggests that differential absorption of left‐ and right‐handed CPL is the primary origin of the observed photocurrent dissymmetry, although additional contributions from spin‐dependent transport or recombination cannot be fully ruled out. The |*g*_ph_| and D^*^ value from our work and previous reports are summarized in Figure 8f, showing that our devices achieve the highest performance among NIR CPL photodetectors. Notably, although these chiral Y6 derivatives have been investigated previously [9, 36], the superior performance achieved in this work originates from supramolecular organization enabled by thermodynamic and kinetic modulation, together with the exploitation of a distinct vertical‐type device architecture. Collectively, this work demonstrates record‐performance NIR CPL photodetectors through a synergistic interplay of supramolecular assembly, processing optimization, and device engineering.

## 3 Conclusion

In this study, we demonstrated supramolecular chirality evolution in LFCs through thermodynamic and kinetic processes in the solid state. IC2F‐B(*S*)DMO‐IC2F films underwent a clear evolution in crystallinity with increasing annealing temperature, marked by a transition from a 3D honeycomb structure to a quasi‐2D supramolecular packing structure. At 250°C, highly ordered hierarchical domains well‐aligned in both vertical and lateral directions were formed. In contrast, IC2Cl‐B(*S*)DMO‐IC2Cl films showed limited structural changes beyond 150°C, with more dispersed stacking orientation. These differences in thermally induced crystallization behavior reflect distinct sensitivities of the two LFCs to supramolecular reorganization and are likely key contributors to the observed disparities in chiroptical activity. Notably, the annealed IC2F‐B(*S*)DMO‐IC2F films achieved a high |*g*_abs_| value of around 0.1. In addition, these high‐performance chiral active layers were integrated into devices to realize SB‐VOFET based NIR CPL photodetectors for the first time. By carefully balancing the gate field effect at the semiconductor/source interface and the conductivity of the source electrodes, SB‐VOFET based CPL photodetectors were successfully fabricated. Owing to the short channel length and efficient photogenerated charge transport in LFC films, the devices exhibited high EQEs exceeding 900% under 850 nm illumination, along with a fast response time of around 600 µs. Moreover, the selective detection of CPL could be effectively tuned through high‐temperature treatment and end‐group engineering, achieving a maximum |*g*_ph_| value of 0.1, which is consistent with the chiroptical properties of the active layers. Overall, our strategy of introducing appropriate chiral alkyl chains into Y6‐based LFCs and employing a SB‐VOFET device configuration synergically enables NIR CPL detectors with record‐high performance.

## Author Contributions

**SungHyun Hur**: investigation, validation. **Jaeyong Ahn**: conceptualization, methodology, writing – original draft, investigation, validation, formal analysis, data curation, visualization. **Sangwook Lee**: investigation, validation. **BongSoo Kim**: conceptualization, writing – review and editing, project administration, resources, funding acquisition, supervision. **Joon Hak Oh**: conceptualization, writing – review and editing, project administration, resources, funding acquisition, supervision. **Kwangmin Kim**: writing – original draft, conceptualization, methodology, investigation, validation, formal analysis, data curation, visualization. **Seul Lee**: investigation, validation.

## Conflicts of Interest

The authors declare no conflicts of interest.

## Supporting information




**Supporting File**: advs76299‐sup‐0001‐SuppMat.pdf.

## Data Availability

The data that support the findings of this study are available from the corresponding author upon reasonable request.
