# Supplementary material for: Thermally Driven Supramolecular Chirality Evolution in Low‐Bandgap Fused‐Ring Conjugated Molecules for High‐Performance NIR Circularly Polarized Light Detection
Source: Adv Sci (Weinh). 2026 Jun 28;13(52):e76299. doi: 10.1002/advs.76299 (PMC13336905; doi:10.1002/advs.76299)
Supplement: Supplementary file 1 [file ADVS-13-e76299-s001.pdf]

Supporting Information

Thermally Driven Supramolecular Chirality Evolution in Low-Bandgap Fused-Ring Conjugated Molecules for High-Performance NIR Circularly Polarized Light Detection

Jaeyong Ahn,[1,2†] *Kwangmin Kim,*[3†] *Sangwook Lee,*[1] *Seul Lee,*[3] *SungHyun Hur,*[4] *BongSoo Kim*[3,4,5,*], *and Joon Hak Oh*[1,*]

Dr. J. Ahn,[1,2] S. Lee,[1] Prof. J. H. Oh[1]
[1]School of Chemical and Biological Engineering, Institute of Chemical Processes, Seoul National University, 1 Gwanak-ro, Gwanak-gu, Seoul 08826, Republic of Korea
[2]Department of Chemical Engineering, Stanford University, Stanford, CA 94305, USA
E-mail: joonhoh@snu.ac.kr

K. Kim,[3] S. Lee,[3] S. Hur,[4] Prof. B. Kim[3,4,5]
[3]Department of Chemistry, Ulsan National Institute of Science and Technology (UNIST), 50 UNIST-gil, Ulsan 44919, Republic of Korea
[4]Graduate School of Semiconductor Materials and Device Engineering, Ulsan National Institute of Science and Technology (UNIST), 50 UNIST-gil, Ulsan 44919, Republic of Korea
[5]Graduate School of Carbon Neutrality, Ulsan National Institute of Science and Technology (UNIST), 50 UNIST-gil, Ulsan 44919, Republic of Korea
E-mail: bongsoo@unist.ac.kr

Keywords: chirality, circularly polarized light, non-fullerene acceptors, vertical transistors, photodetectors

[†] These authors contributed equally to this work

Experimental Details

Material Synthesis

Scheme S1. Synthetic Routes of IC2F-B(*S*)-IC2F, IC2F-B(*R*)DMO-IC2F, IC2Cl-B(*S*)DMO-IC2Cl and IC2Cl-B(*R*)DMO-IC2Cl.

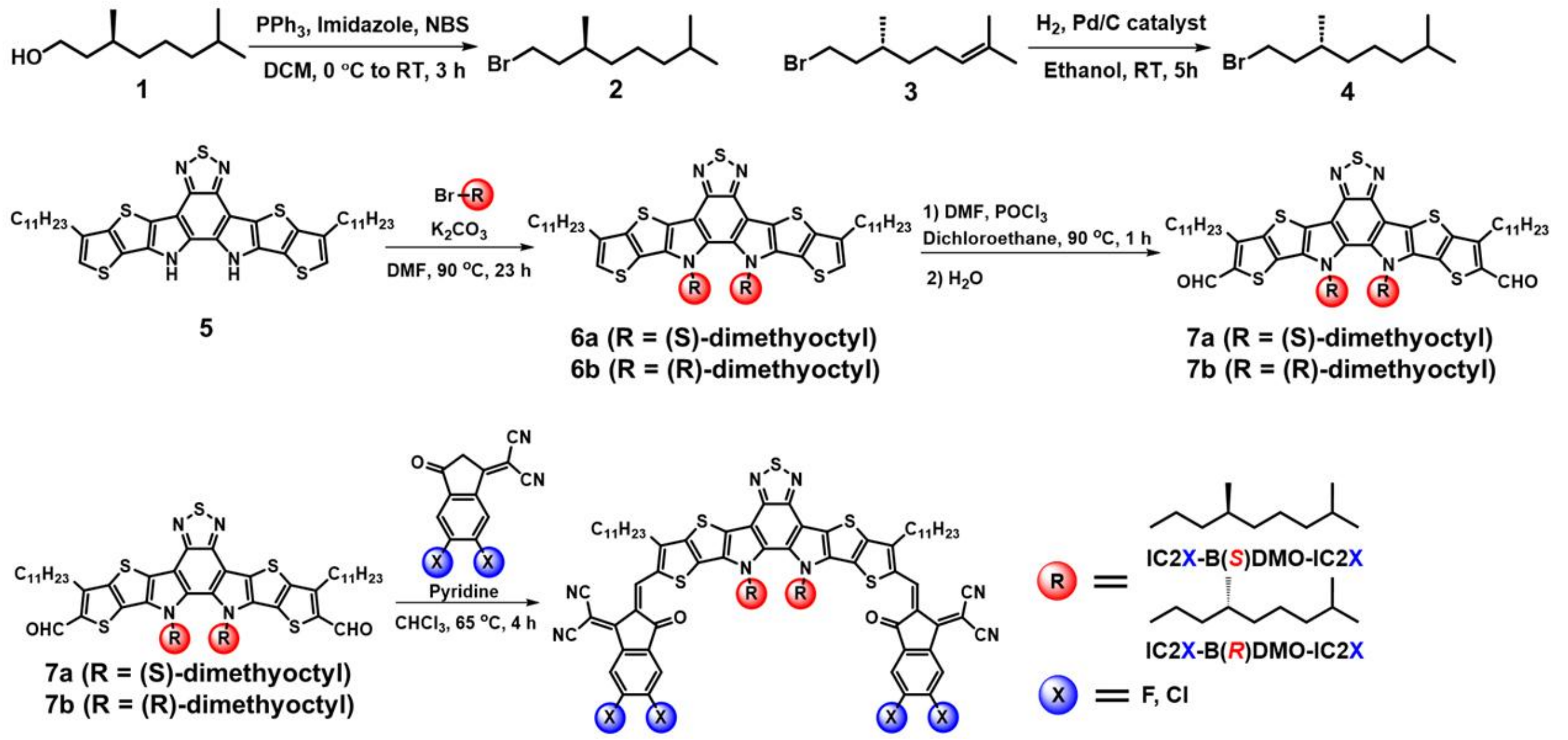

Chemicals and Materials for synthesis: Compound **1** was purchased from Arctom Scientific (Westlake Village, CA, USA). Compound **3** was purchased from Sigma-Aldrich Korea (Yongin, Republic of Korea). Compound **5** was purchased from JavaChem Co., Ltd (Seongnam, Republic of Korea). 2-(5,6-difluoro-3-oxo-2,3-dihydro-1*H*-inden-1-ylidene)malononitrile (IC2F) and 2-(5,6-dichloro-3-oxo-2,3-dihydro-1*H*-inden-1-ylidene)malononitrile (IC2Cl) were purchased from Aaron Pharmatech Ltd (Hangzhou, China). All low-bandgap fused-ring conjugated molecules (LFCs) share similar synthetic routes to Y6 except for the chiral side chain.[S1] Deuterated chloroform ($CDCl_3$) for measurement of ^{1}H-NMR spectrum was purchased from Cambridge Isotope Laboratories, Inc (Tewksbury, MA, USA). Tetrabutylammonium hexafluorophosphate ($TBAPF_6$) was purchased from Tokyo Chemical Industry Co., Ltd

(Tokyo, Japan). Other chemicals were purchased from commercial sources and used without further purification if not mentioned.

Synthesis of Synthesis of (*S*)-1-bromo-3,7-dimethyloctane (2): Imidazole (323 mg, 4.74 mmol) and PPh_3 (1,243 mg, 4.74 mmol) were dissolved in anhydrous dichloromethane (6.3 mL), and compound **1** (500 mg, 3.16 mmol) was added to the reaction mixture. After that, NBS (843 mg, 4.74 mmol) was added portion-wise at 0 °C, and the mixture was stirred at room temperature for 16 h. After reaction, the organic layer was extracted using 60 mL of dichloromethane and 30 mL of distilled water three times. The remaining water was removed by $MgSO_4$. Organic solvents were dried under reduced pressure. The resulting crude product was purified by silica gel column chromatography using *n*-hexane as the eluent to obtain compound **2** as a transparent liquid (669 mg, 96%). ^{1}H-NMR (400 MHz, $CDCl_3$) δ (ppm): 3.44-3.57 (m, 2H), 1.87-1.89 (m, 1H), 1.63-1.69 (m, 2H), 1.51-1.54 (m, 1H) 1.13-1.28 (m, 6H), 0.86-0.90 (t, 9H).

Synthesis of Synthesis of (*R*)-1-bromo-3,7-dimethyloctane (4): Compound **3** (1 g, 4.56 mmol) and Pd/C catalyst (51.2 mg, 0.45 mmol) were added to a round-bottom flask, and ethanol (4 mL) was added under an argon atmosphere. Hydrogen was supplied via a balloon connected to the flask and stirred at room temperature for 5 h. After 5 h, the reaction mixture was first filtered to remove the catalyst, followed by vacuum evaporation to obtain compound **4** as a transparent liquid (890 mg, 88%). ^{1}H-NMR (400 MHz, $CDCl_3$) δ (ppm): 3.39-3.47 (m, 2H), 1.85-1.92 (m, 1H), 1.62-1.69 (m, 2H), 1.49-1.54 (m, 1H) 1.13-1.28 (m, 6H), 0.86-0.89 (t, 9H).

Synthesis of 12,13-bis((*S*)-3,7-dimethyloctyl)-3,9-diundecyl-12,13-dihydro-[1,2,5]thiadiazolo[3,4-*e*]thieno[2'',3'':4',5']thieno[2',3':4,5]pyrrolo[3,2-*g*]thieno[2',3':4,5]thieno[3,2-*b*]indole (6a): Compound **5** (373.6 mg, 0.50 mmol) and K_2CO_3 (276 mg, 2.00 mmol) were

added to a round-bottom flask, and dry DMF (5 mL) was added under an argon atmosphere. Then, compound 2 (442.4 mg, 2.00 mmol) was added and stirred at 90 °C for 22 h. After drying the DMF under reduced pressure, the organic layer was extracted three times using 60 mL of chloroform and 30 mL of distilled water. The remaining water was removed by $MgSO_4$. Organic solvents were dried under reduced pressure. The resulting crude product was purified by silica gel column chromatography using chloroform:*n*-hexane (1:2, v/v) as an eluent to obtain compound **6a** as an orange solid (263 mg, 51%). ^{1}H-NMR (400 MHz, $CDCl_3$) δ (ppm): 7.08 (s, 2H), 4.72-4.79 (m, 4H), 2.87-2.91 (t, 4H), 0.91-1.95 (m, 68H), 0.77–0.79 (dd, 12H).

Synthesis of 12,13-bis((*R*)-3,7-dimethyloctyl)-3,9-diundecyl-12,13-dihydro-[1,2,5]thiadiazolo[3,4-*e*]thieno[2'',3'':4',5']thieno[2',3':4,5]pyrrolo[3,2-*g*]thieno[2',3':4,5]thieno[3,2-*b*]indole (6b): Followed by the above synthetic procedure of compound **6b**. Yield: 414 mg (75%), ^{1}H-NMR (400 MHz, $CDCl_3$) δ (ppm): 7.02 (s, 2H), 4.61-4.74 (m, 4H), 2.87-2.91 (t, 4H), 0.91-1.95 (m, 68H), 0.77–0.79 (dd, 12H).

Synthesis of 12,13-bis((*S*)-3,7-dimethyloctyl)-3,9-diundecyl-12,13-dihydro-[1,2,5]thiadiazolo[3,4-*e*]thieno[2'',3'':4',5']thieno[2',3':4,5]pyrrolo[3,2-*g*]thieno[2',3':4,5]thieno[3,2-*b*]indole-2,10-dicarbaldehyde (7a): $POCl_3$ (235 mg, 1.53 mmol) was added dropwise to the dry DMF (112 mg, 1.53 mmol) at 0 °C, and the solution was stirred at RT for 1 h. Then, compound **6a** (263 mg, 0.26 mmol) was dissolved in 1,2-dichloroethane (3 mL) and added to the reaction mixture, and stirred at 90 °C for 1 h. Then, 30 mL of distilled water was added to the reaction mixture. The organic layer was extracted with 60 mL of chloroform three times. The organic layer was further purified by column chromatography using chloroform as an eluent to yield compound **7a** as a yellow solid (255 mg, 92% yield). ^{1}H-NMR (400 MHz, $CDCl_3$) δ

(ppm): 10.14 (s, 2H), 4.69-4.77 (m, 4H), 3.17-3.21 (t, 4H), 0.84–2.17 (m, 68H), 0.68–0.70 (dd, 12H).

Synthesis of 12,13-bis((*R*)-3,7-dimethyloctyl)-3,9-diundecyl-12,13-dihydro-[1,2,5]thiadiazolo[3,4-*e*]thieno[2'',3'':4',5']thieno[2',3':4,5]pyrrolo[3,2-*g*]thieno[2',3':4,5]thieno[3,2-*b*]indole-2,10-dicarbaldehyde (7b): Followed by the above synthetic procedure of compound **7a**. Yield: 352 mg (80%), ^{1}H-NMR (400 MHz, $CDCl_3$) δ (ppm): 10.14 (s, 2H), 4.70-4.77 (m, 4H), 3.18-3.22 (t, 4H), 0.83–1.94 (m, 68H), 0.67–0.68 (dd, 12H).

Synthesis of 2,2'-((2*Z*,2'*Z*)-((12,13-bis((*S*)-3,7-dimethyloctyl)-3,9-diundecyl-12,13-dihydro-[1,2,5]thiadiazolo[3,4-*e*]thieno[2'',3'':4',5']thieno[2',3':4,5]pyrrolo[3,2-*g*]thieno[2',3':4,5]thieno[3,2-*b*]indole-2,10-diyl)bis(methaneylylidene))bis(5,6-difluoro-3-oxo-2,3-dihydro-1*H*-indene-2,1-diylidene))dimalononitrile (IC2F-B(*S*)DMO-IC2F): Compound **7a** (127 mg, 0.117 mmol) and IC2F (135 mg, 0.585 mmol) were added to a round-bottom flask, and anhydrous chloroform (10 mL) was added under an argon atmosphere. Then, pyridine (0.1 mL) was added, and the mixture was stirred at 65 °C for 4 h. The reaction mixture was cooled to room temperature, and the solvent was evaporated. The resulting crude product was purified by silica gel column chromatography using chloroform:*n*-hexane (3:1, v/v) as an eluent to obtain IC2F-B(*S*)DMO-IC2F as a dark blue solid (149 g, 85%). ^{1}H-NMR (400 MHz, $CDCl_3$) δ (ppm): 8.79 (s, 2H), 8.43-8.47 (dd, 2H), 7.61-7.65 (t, 2H), 4.70-4.79(m, 4H), 2.99-3.03 (t, 4H), 2.00 (m, 2H), 1.73-1.79 (m, 6H) 0.85–1.47 (m, 60H), 0.71-0.73 (dd, 12H) ^{13}C NMR (100 MHz, $CDCl_3$) δ (ppm): 185.84, 157.54, 153.64, 146.93, 145.04, 137.05, 132.62, 132.42, 130.47, 119.52, 114.52, 114.39, 113.35, 68.92, 50.28, 39.10, 38.25, 37.52, 31.93, 31.48, 30.87, 29.82, 29.71, 29.65, 29.52, 29.41, 29.36, 27.96, 24.82, 22.71, 22.59, 22.53, 19.66, 14.15. MS (MALDI-TOF): Calcd. for $C_{86}H_{94}F_4N_8O_2S_5$ [M]+: 1506.60, Found: 1507.635.

Synthesis of 2,2'-((2*Z*,2'*Z*)-((12,13-bis((*R*)-3,7-dimethyloctyl)-3,9-diundecyl-12,13-dihydro-[1,2,5]thiadiazolo[3,4-*e*]thieno[2'',3'':4',5']thieno[2',3':4,5]pyrrolo[3,2-*g*]thieno[2',3':4,5]thieno[3,2-*b*]indole-2,10-diyl)bis(methaneylylidene))bis(5,6-difluoro-3-oxo-2,3-dihydro-1*H*-indene-2,1-diylidene))dimalononitrile (IC2F-B(*R*)DMO-IC2F): Followed by the above synthetic procedure of IC2F-B(*S*)DMO-IC2F. Yield: 180 mg (76%), 8.85 (s, 2H), 8.45-8.49 (t, 2H), 7.62-7.66 (t, 2H), 4.73-4.80(m, 4H), 3.05 (t, 4H), 1.99 (m, 2H), 1.76-1.78 (m, 6H) 0.85–1.48 (m, 60H), 0.70-0.72 (dd, 12H) ^{13}C NMR (100 MHz, $CDCl_3$) δ (ppm): 185.86, 157.60, 153.65, 146.95, 146.05, 137.07, 134.04, 132.63, 132.46, 130.49, 119.53, 114.83, 114.54, 114.40, 113.47, 110.00, 68.92, 50.28, 39.09, 38.23, 37.50, 31.93, 31.46, 30.89, 29.82, 29.71, 29.66, 29.64, 29.52, 29.42, 29.37, 27.95, 24.82, 22.71, 22.58, 22.52, 19.67, 14.15. MS (MALDI-TOF): Calcd. for $C_{86}H_{94}F_4N_8O_2S_5$ [M]+: 1506.60, Found: 1507.635.

Synthesis of 2,2'-((2*Z*,2'*Z*)-((12,13-bis((*S*)-3,7-dimethyloctyl)-3,9-diundecyl-12,13-dihydro-[1,2,5]thiadiazolo[3,4-*e*]thieno[2'',3'':4',5']thieno[2',3':4,5]pyrrolo[3,2-*g*]thieno[2',3':4,5]thieno[3,2-b]indole-2,10-diyl)bis(methaneylylidene))bis(5,6-dichloro-3-oxo-2,3-dihydro-1*H*-indene-2,1-diylidene))dimalononitrile (IC2Cl-B(*S*)DMO-IC2Cl): Followed by the above synthetic procedure of IC2F-B(*S*)DMO-IC2F. Yield: 133 mg (72%), ^{1}H-NMR (400 MHz, $CDCl_3$) δ (ppm): 8.72 (s, 2H), 8.67 (s, 2H), 7.85 (s, 2H), 4.69-4.76 (m, 4H), 2.96 (t, 4H), 2.01 (m, 2H), 1.71-1.76 (m, 6H) 0.85–1.45 (m, 60H), 0.74-0.76 (dd, 12H) ^{13}C NMR (100 MHz, $CDCl_3$) δ (ppm): 185.87, 153.80, 146.89, 145.15, 139.23, 138.85, 138.34, 137.02, 135.61, 134.54, 132.99, 132.39, 130.71, 126.62, 119.47, 114.63, 114.43, 113.50, 69.03, 39.14, 37.56, 31.93, 31.55, 30.84, 29.82, 29.71, 29.64, 29.51, 29.40, 29.36, 27.99, 24.88, 22.71, 22.64, 22.58, 19.69, 14.15. MS (MALDI-TOF): Calcd. for $C_{86}H_{94}Cl_4N_8O_2S_5$ [M]+: 1570.49, Found: 1572.556.

Synthesis of 2,2'-((2*Z*,2'*Z*)-((12,13-bis((*R*)-3,7-dimethyloctyl)-3,9-diundecyl-12,13-dihydro-[1,2,5]thiadiazolo[3,4-*e*]thieno[2'',3'':4',5']thieno[2',3':4,5]pyrrolo[3,2-*g*]thieno[2',3':4,5]thieno[3,2-*b*]indole-2,10-diyl)bis(methaneylylidene))bis(5,6-dichloro-3-oxo-2,3-dihydro-1*H*-indene-2,1-diylidene))dimalononitrile (IC2Cl-B(*R*)DMO-IC2Cl): Followed by the above synthetic procedure of IC2F-B(*S*)DMO-IC2F. Yield: 205 mg (82%), ^{1}H-NMR (400 MHz, $CDCl_3$) δ (ppm): 8.64 (s, 4H), 7.82 (s, 2H), 4.61-4.78 (m, 4H), 2.91 (t, 4H), 2.03 (m, 2H), 1.68-1.82 (m, 6H) 0.85–1.48 (m, 60H), 0.76-0.77 (d, 12H). ^{13}C NMR (100 MHz, $CDCl_3$) δ (ppm): 185.85, 157.29, 153.78, 146.84, 145.14, 139.23, 138.29, 138.84, 138.29, 135.56, 132.99, 132.34, 126.58, 124.50, 119.44, 114.62, 114.41, 69.01, 39.16, 37.60, 31.93, 31.61, 30.82, 30.16, 29.83, 29.71, 29.65, 29.52, 29.40, 29.36, 27.99, 24.89, 22.71, 22.65, 22.59, 19.68, 14.15. MS (MALDI-TOF): Calcd. for $C_{86}H_{94}Cl_4N_8O_2S_5$ [M]+: 1570.49, Found: 1574.566.

Instrumentation

Nuclear magnetic resonance (NMR) spectroscopy measurements: ^{1}H and ^{13}C-NMR spectrum was obtained on a Bruker Avance III HD 400 MHz and Agilent 400 MHz NMR spectrometer using $CDCl_3$ as a solvent.

Matrix-assisted laser desorption ionization-time of flight (MALDI-TOF) spectroscopy measurements: The molecular mass was confirmed by Bruker Autoflex max MALDI-TOF mass spectrometer, which is located in UNIST central research facilities (UCRF).

Glass transition temperature (T_g) measurement using temperature-dependent UV-visible absorption spectra[S2]: Deviation metric (DMT) that is the sum of the squared deviation in the absorbance between as-cast and annealed films

$$DM_T = \sum_{\lambda_{min}}^{\lambda_{max}} [I_{RT}(\lambda) - I_T(\lambda)]^2$$

where λ is the wavelength, λ_{min} and λ_{max} are the lower and upper bounds of the UV-visible absorption spectra, and I_{RT} (λ) and I_T (λ) are the normalized absorption intensities of the as-cast (room temperature) and annealed films, respectively. The films were annealed for 5 min on the surface of a hot plate and allowed to cool to room temperature (RT, ~25 °C) for 3 min prior to acquiring their annealed spectra. Each film was annealed in increments of temperature from RT (10 °C).

Cyclic voltammetry (CV) measurements: CV was performed using a CorrTest instrument electrochemical analyzer and degassed acetonitrile solution containing 0.1 M tetrabutylammonium hexafluorophosphate ($TBAPF_6$) was used as an electrolyte. The voltage sweep rate was 50 mV/s. A Pt wire electrode coated with a thin film of the molecule was used as the working

electrode, and another Pt wire was used as the counter electrode; Ag/AgCl was the reference electrode, and ferrocene (Fc) was used as the internal standard (Fc/Fc^+ potential was assumed to be -4.8 eV).

Optical and chiroptical analysis: A JASCO V-770 UV–visible spectrometer was used to perform the spectral measurements for UV–visible absorption. CD spectra were measured using a J-815 spectropolarimeter (JASCO). All thin films were deposited on glass quartz substrates via spin-coating at an optimized spin speed, and the spectra were obtained before and after thermal annealing the samples under a nitrogen (N_2) atmosphere.

Thermal analysis: TGA measurements were performed using Perkin Elmer TGA Pyris 6 from 50 °C to 500 °C at a heating rate of 10 °C /min under a nitrogen purge. DSC measurement was conducted on a Q200 Differential Scanning Calorimeter by TA Instruments at a heating rate of 10 °C/min between 0 and 270 °C under nitrogen.

Single crystal growth method: A ternary solvent system was utilized to grow the single crystals. In the NMR tube, 600 μL chiral LFCs solution (3 mg mL^{-1} in chlorobenzene) was added. A 400 μL toluene added along the wall of the tube onto the solution layer. The toluene acts as a buffer layer. A 1 mL methanol added along the wall of the tube onto the toluene, avoiding the interfaces being destroyed. The tube was placed in a quiet place to grow the single crystal slowly.

Single crystal X-ray crystallographic analysis: A crystal of chiral LFCs were coated with paratone-N oil and the diffraction data measured at 100 K with synchrotron radiation (I=0.63000 Å) on a Rayonix SX165 detector at BL2D SMC with a silicon (111) double crystal

monochromator at the Pohang Accelerator Laboratory, Korea. The PAL BL2D-SMDC program[S3] was used for data collection (detector distance is 66 mm, omega scan; Δω=3°, exposure time is 0.6 sec per frame) and HKL3000sm (Ver.716.7)[S4] was used for cell refinement, reduction and absorption correction. The crystal structure of chiral LFCs were solved by the intrinsic phasing method with SHELXT-2018 program[S5] and refined by full-matrix least-squares calculations with the SHELXL-2018 program.[S6]

Morphological analysis: AFM images of the films were obtained from a Dimension ICON (Bruker Nano Surface). The samples were prepared by spin-coating method on the bare glass.

Grazing incidence wide angle X-ray scattering (GIWAXS): GIWAXS measurements were carried out at PLS-II 6D UNIST-PAL beamline of Pohang Accelerator Laboratory (PAL) in Pohang, Republic of Korea. The energy of X-ray source was 11.564 keV and the sample-to-detector distance was about 240 mm. GIWAXS patterns were recorded with a 2D-CCD detector (MX225-HS, Rayonix LLC, USA). The samples were prepared by spin-coating method on the silicon wafer. The GIWAXS images were processed using pGIXS program[S7].

Materials for device fabrication: Anhydrous chloroform, isopropyl alcohol, and bathocuproine were obtained from Sigma-Aldrich. AgNWs for source networks were purchased from NIC Corp., Ltd (0.5 wt% dispersed in IPA; length ~20 μm; diameter ~20 nm).

Device fabrication: To make the source electrode contact pads, a 4/40 nm Cr/Au metal layer was thermally deposited under high vacuum conditions (< 3.0×10^{-6} Torr) using a shadow mask. Subsequently, AgNWs dispersed in isopropylalcohol (IPA) were spin-coated onto the substrate, followed by thermal annealing at 100 °C for 10 minutes. The chiral LFC material

was dissolved in chloroform (CF) at a concentration of 10 mg/mL, and the active layer was formed via 1000 rpm spin-coating. The prepared film was thermally annealed for 10 minutes to induce the desired chiroptical properties, followed by slow cooling to room temperature. Finally, the drain electrodes consisted of 9/15 nm BCP/Ag were deposited through a shadow mask (length and width of 200 μm) via thermal evaporation under high vacuum conditions ($< 3.0 \times 10^{-6}$ Torr).

Opto-electrical measurements: The optoelectronic characteristics of the devices were measured in a vacuum environment using a Keithley 4200-SCS semiconductor parameter analyzer. CPL was generated through a linear polarizer and a quarter-wave plate (Thorlab) between the light source and samples. To evaluate the performance of the SB-VOFETs, R and EQE were calculated using the following equations.

$$R = \frac{I_{ph}}{P_{inc}} = \frac{I_{light} - I_{dark}}{P_{inc}}$$

$$EQE = \frac{hcI_{ph}}{e\lambda P_{inc}} = \frac{hc}{e\lambda}R$$

In this equation, I_{ph} is photocurrent, P_{inc} is the incident illumination power, I_{light} is the current measured under light illumination conditions, and I_{dark} is the drain current measured under dark light conditions. Furthermore, h is Planck's constant, c is the speed of light, and λ represents the wavelength of the light respectively. D^* is calculated using the following equation under estimation where shot noise is the dominant noise source.

$$D* = \frac{R\sqrt{A}}{\sqrt{2eI_{dark}}}$$

Supplementary Figures and Tables

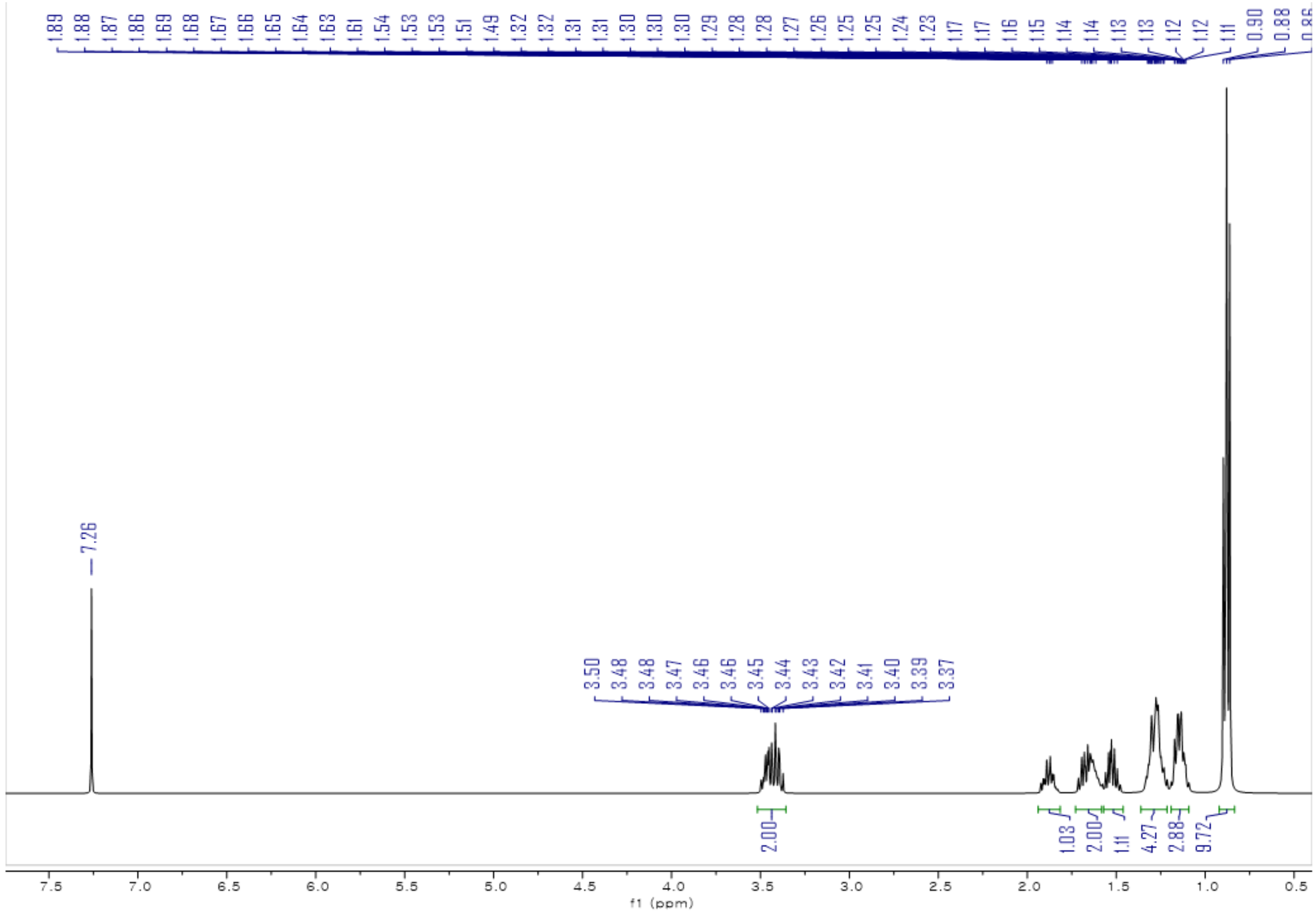

Figure S1. ^{1}H-NMR (400 MHz, $CDCl_3$) spectrum of compound 2.

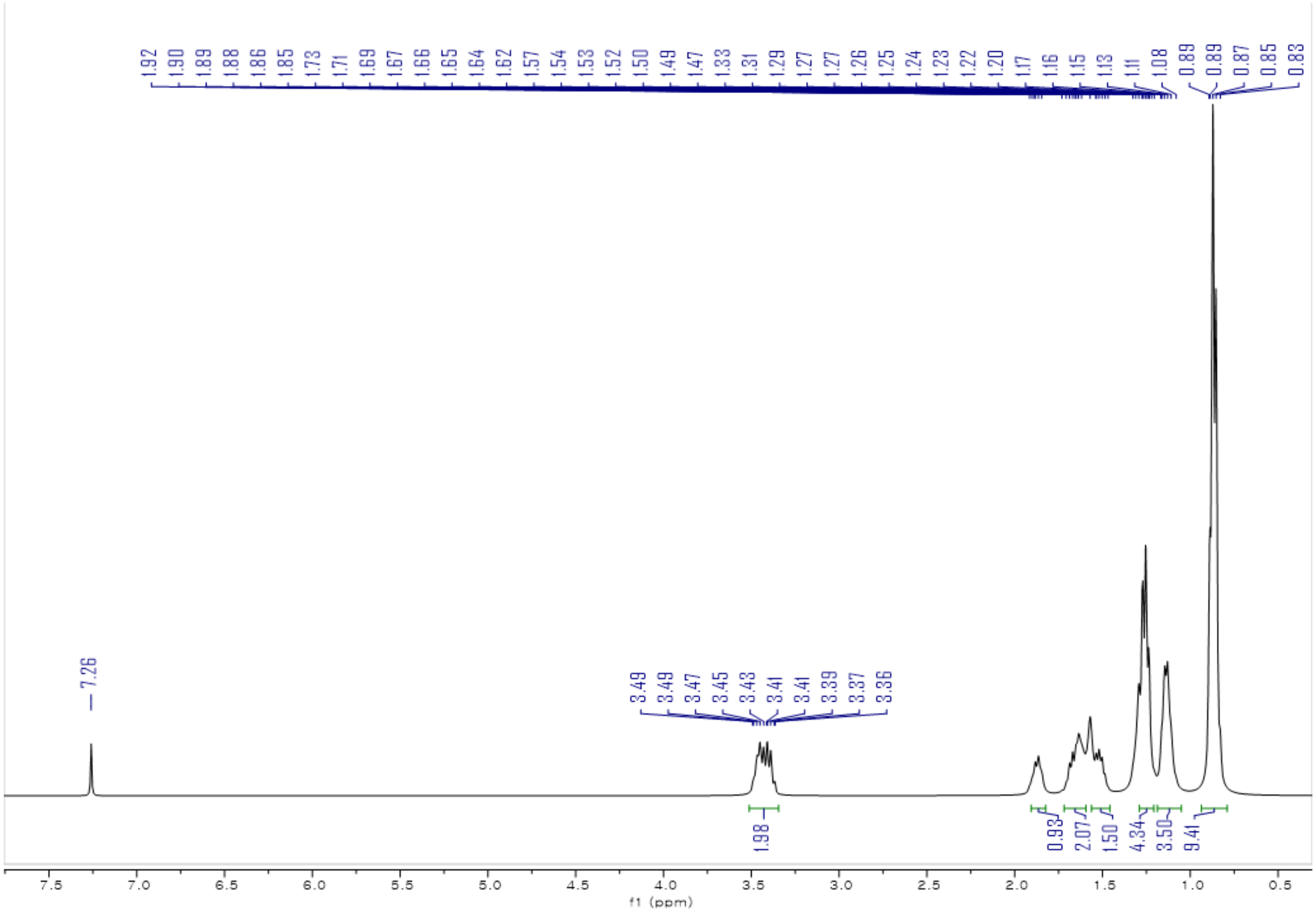

Figure S2. ^{1}H-NMR (400 MHz, $CDCl_3$) spectrum of compound 4.

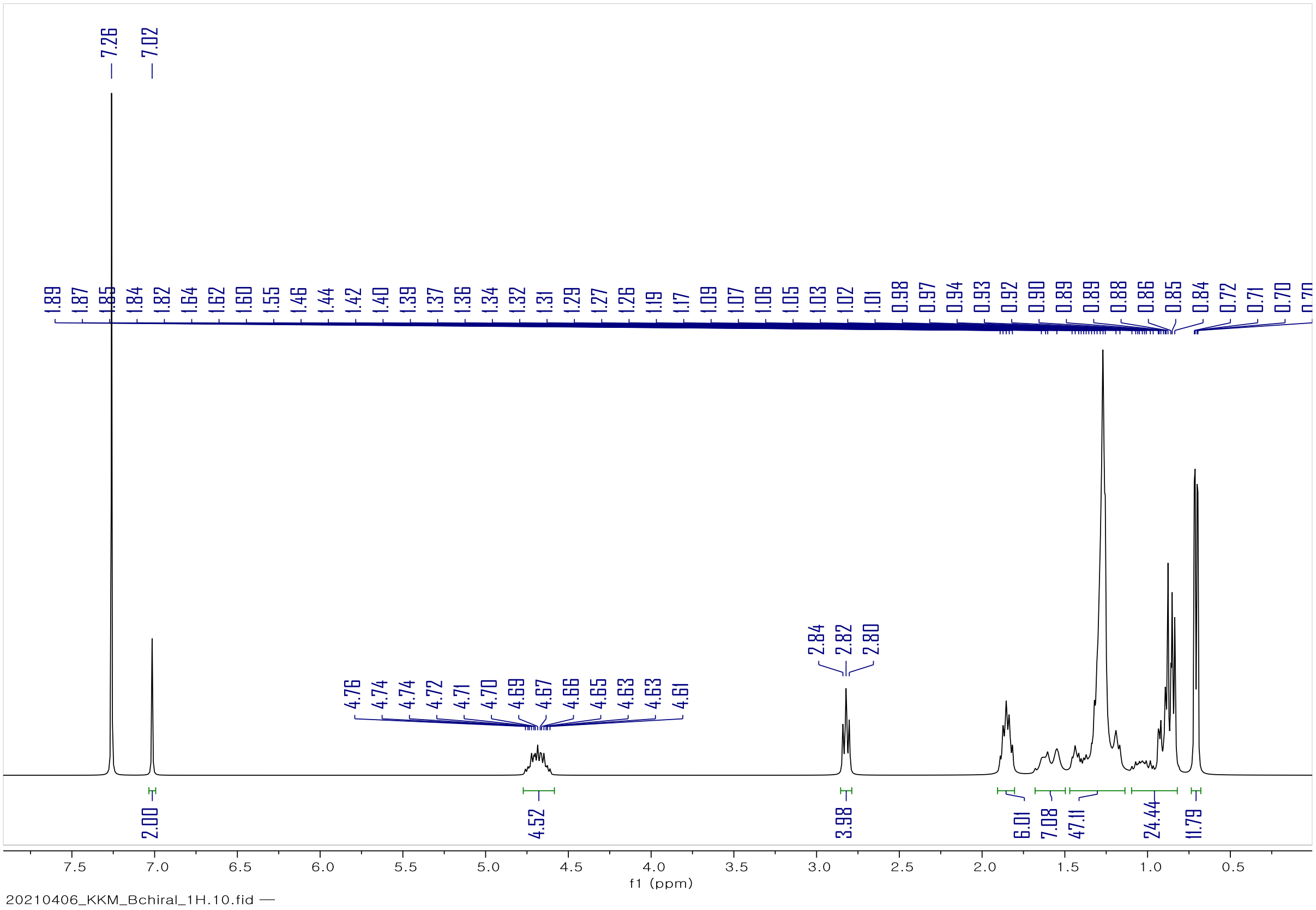

Figure S3. ^{1}H-NMR (400 MHz, $CDCl_3$) spectrum of compound 6a.

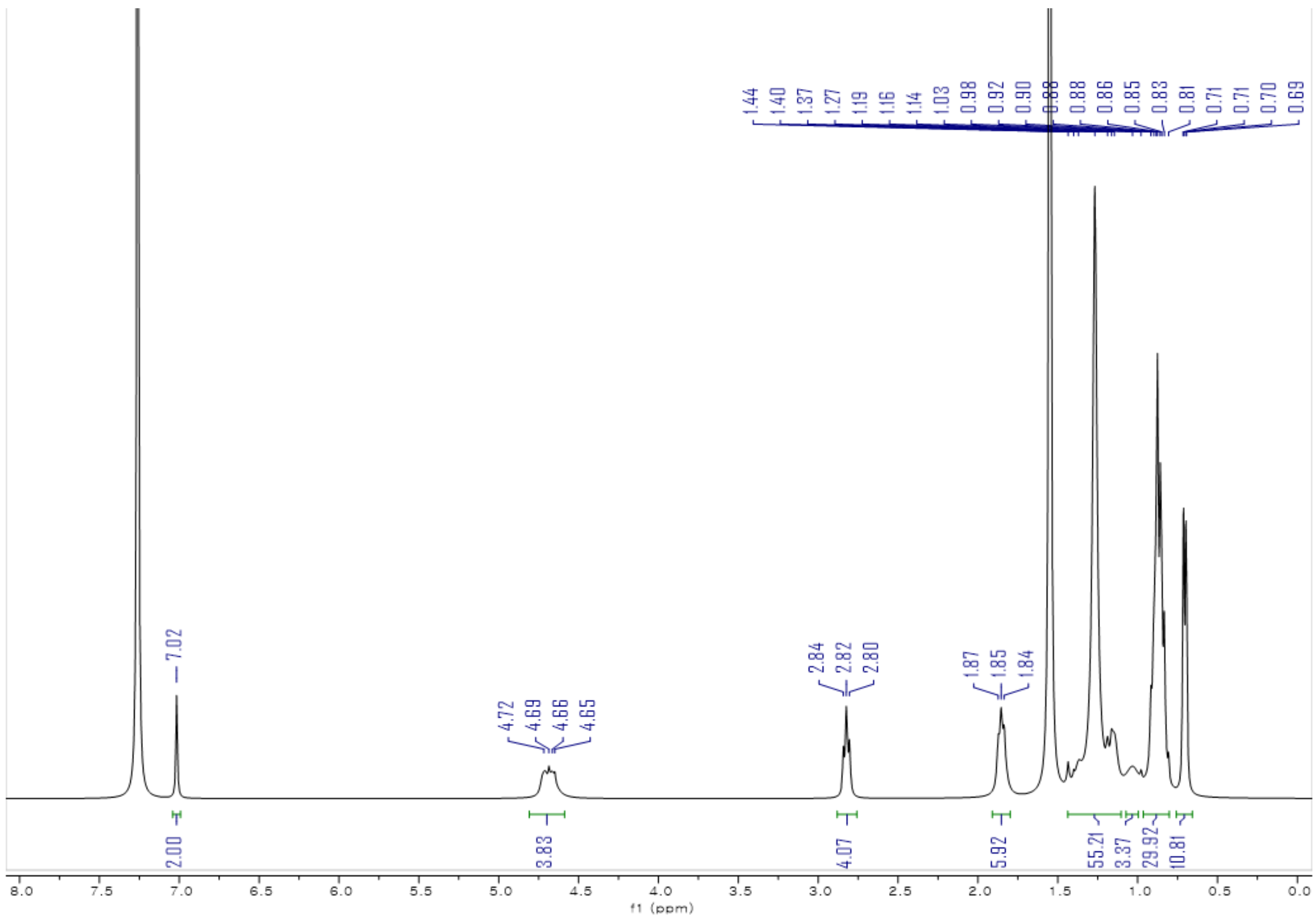

Figure S4. ^{1}H-NMR (400 MHz, $CDCl_3$) spectrum of compound 6b.

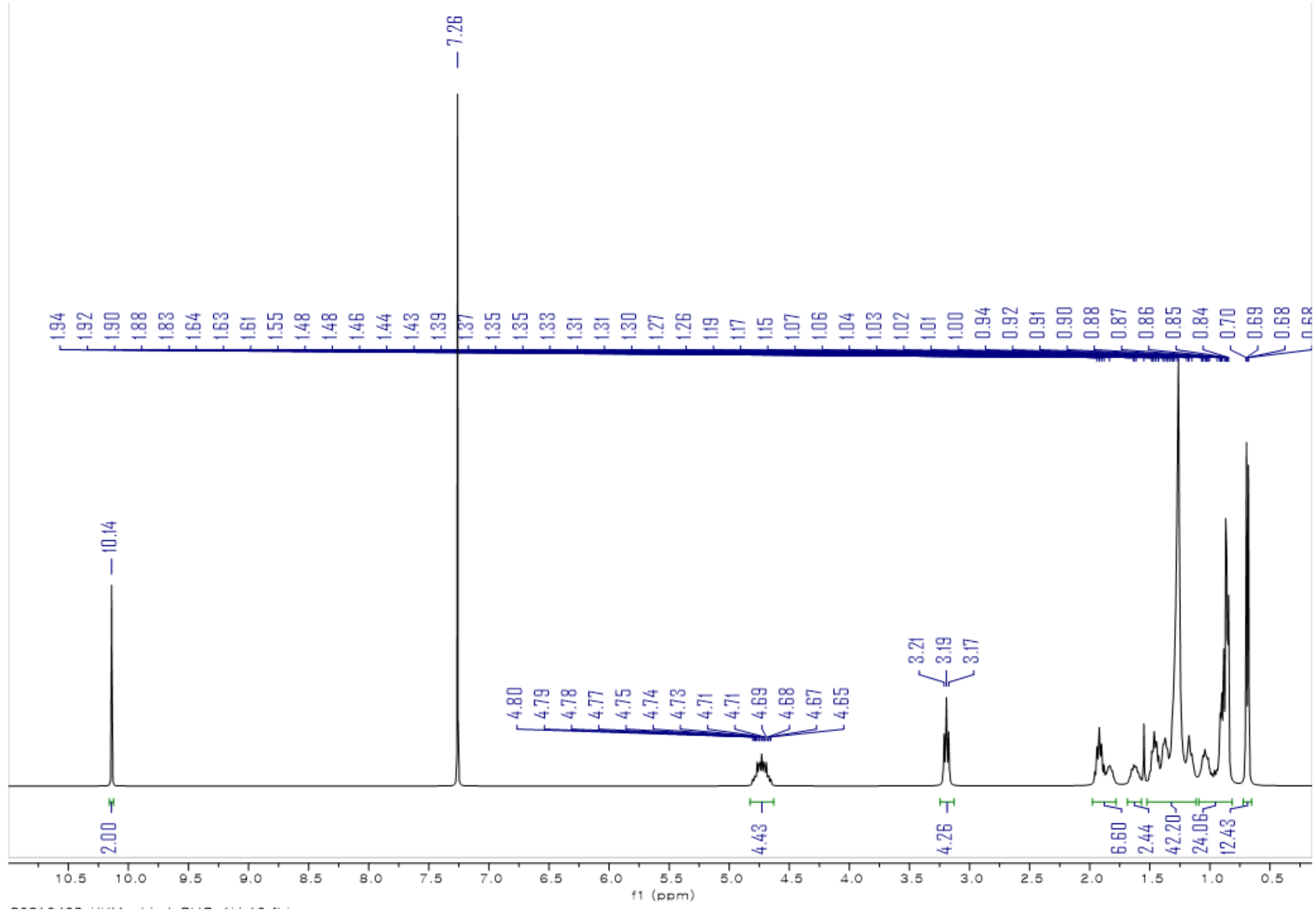

Figure S5. ^{1}H-NMR (400 MHz, $CDCl_3$) spectrum of compound 7a.

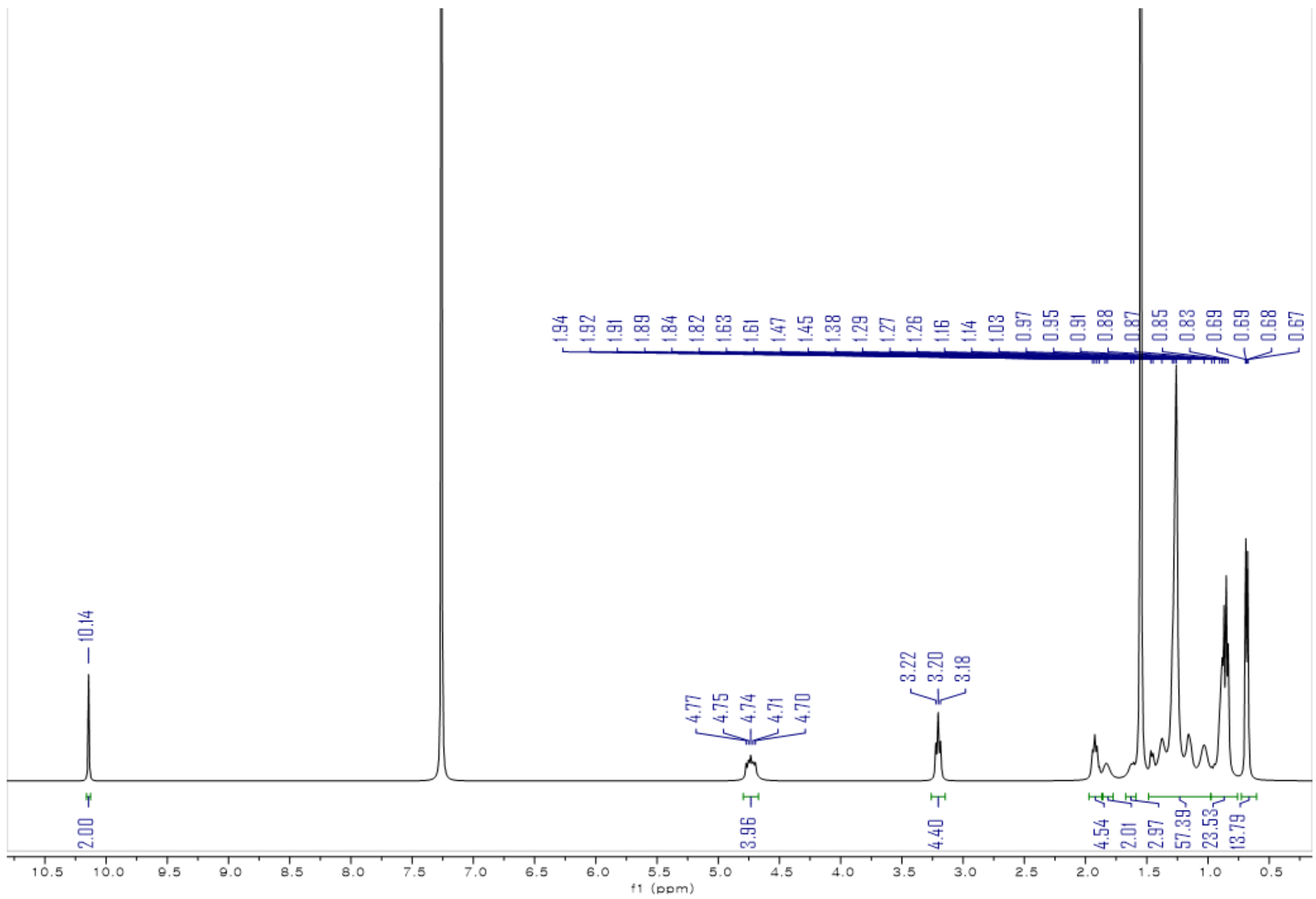

Figure S6. ^{1}H-NMR (400 MHz, $CDCl_3$) spectrum of compound 7b.

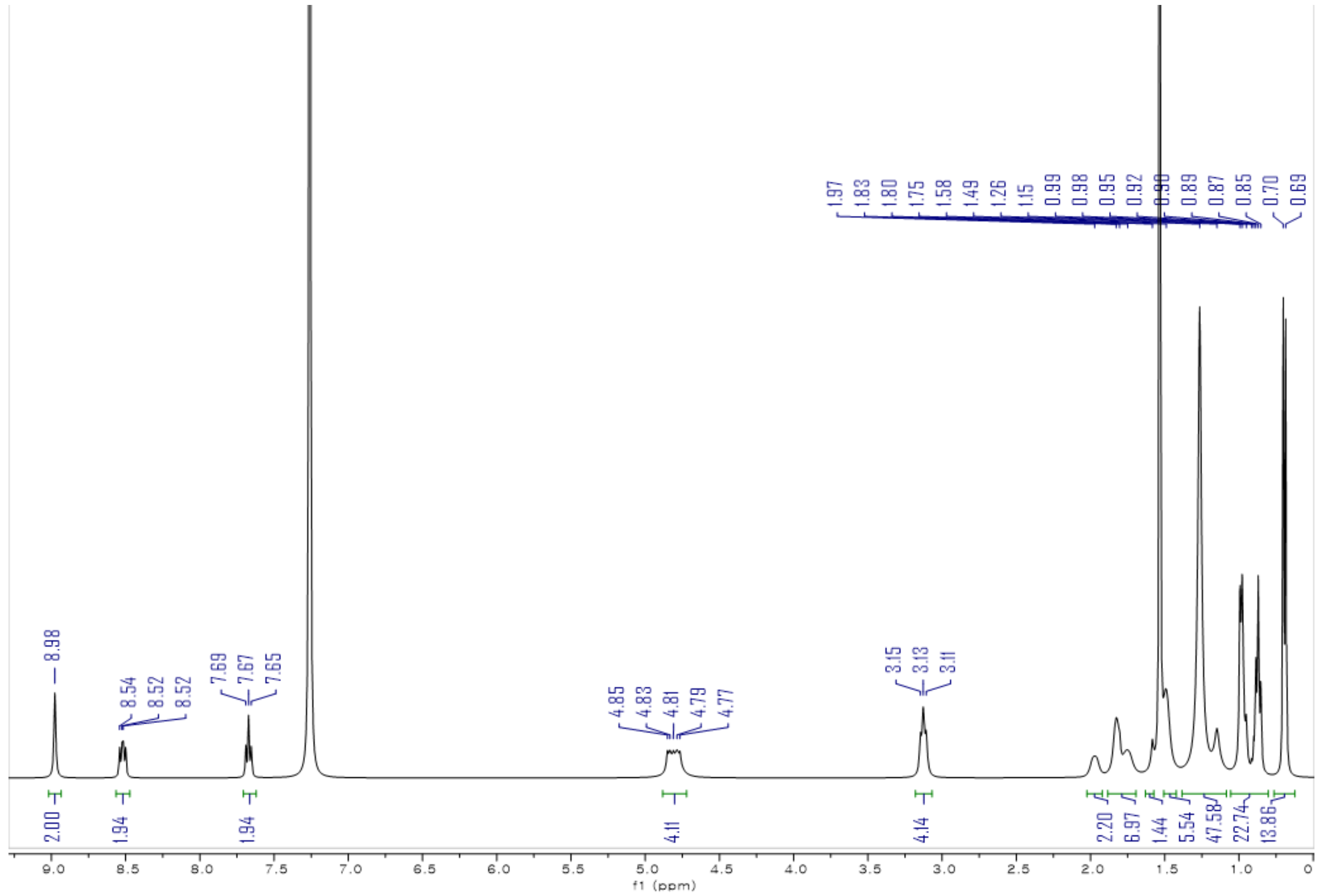

Figure S7. ^{1}H-NMR (400 MHz, $CDCl_3$) spectrum of compound IC2F-B(*S*)DMO-IC2F.

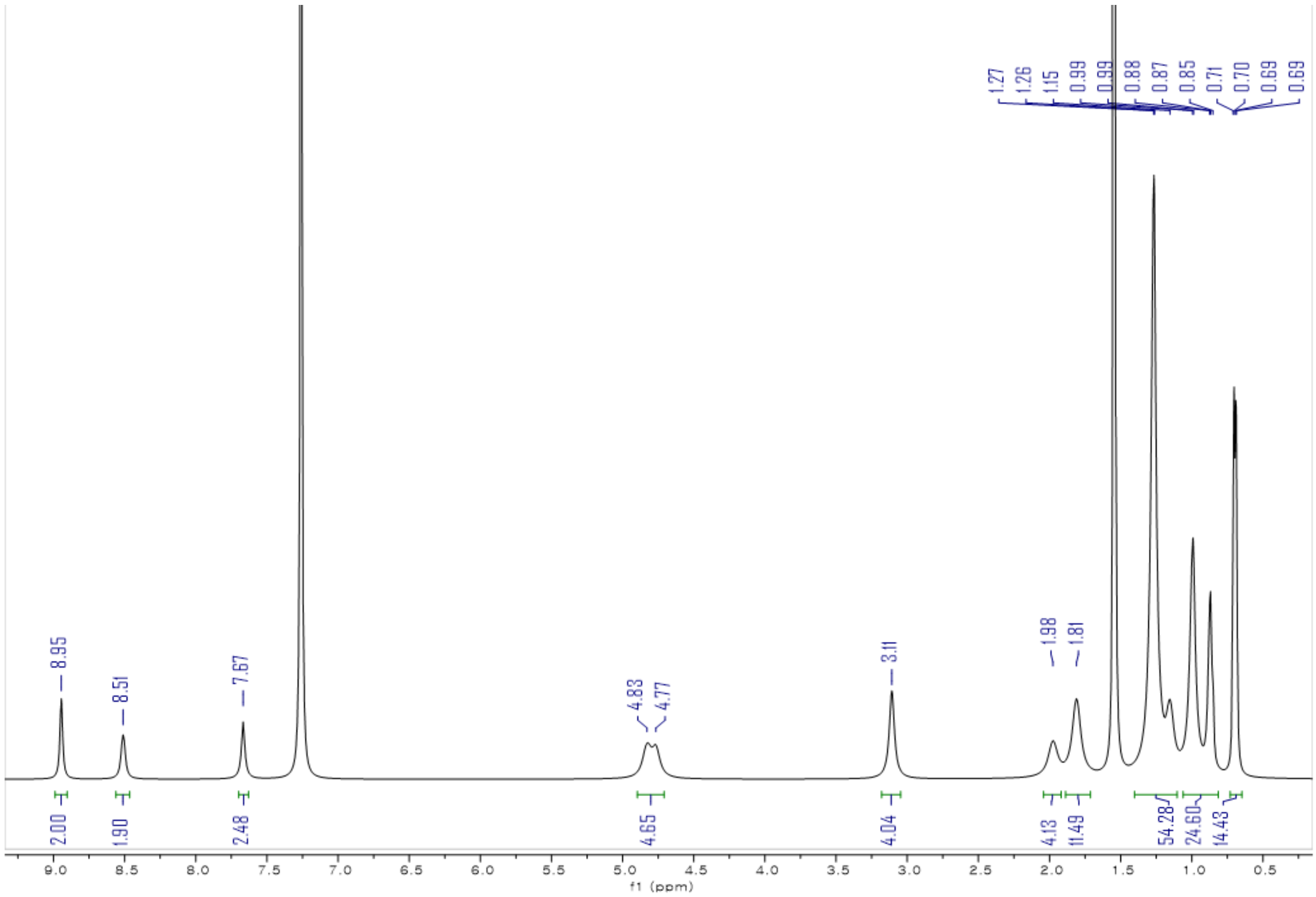

Figure S8. ^{1}H-NMR (400 MHz, $CDCl_3$) spectrum of IC2F-B(*R*)DMO-IC2F.

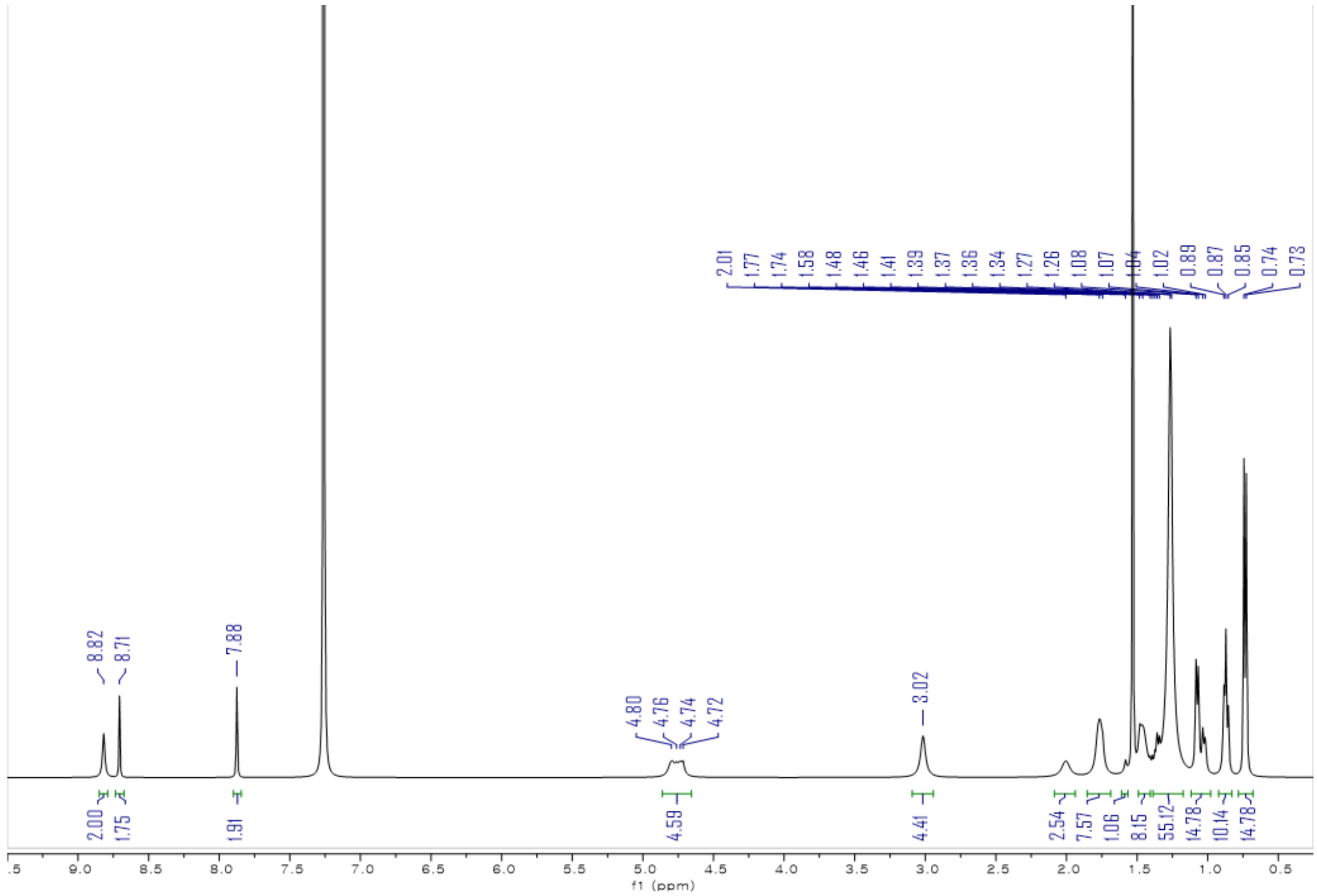

Figure S9. ^{1}H-NMR (400 MHz, $CDCl_3$) spectrum of IC2Cl-B(*S*)DMO-IC2Cl.

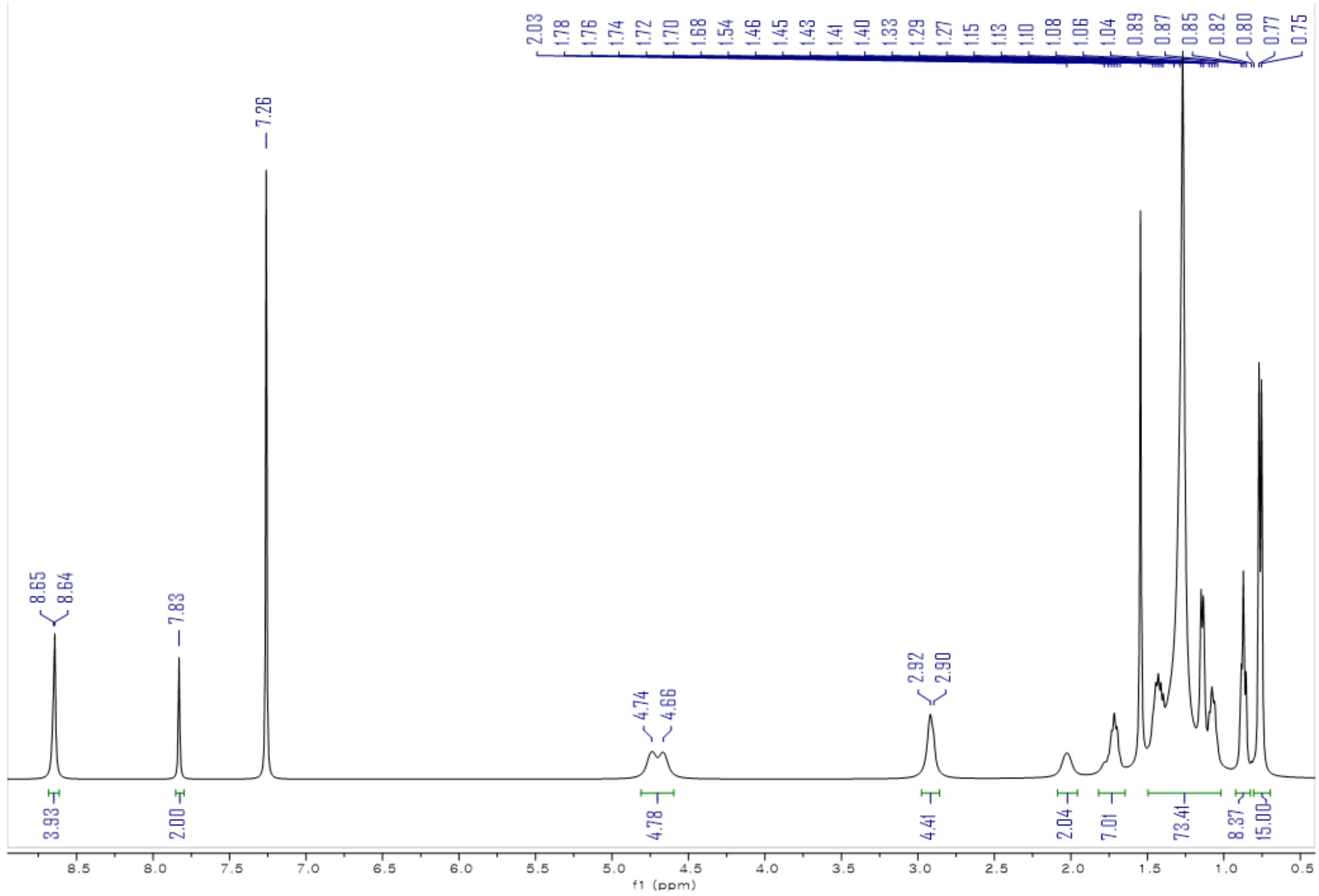

Figure S10. ^{1}H-NMR (400 MHz, $CDCl_3$) spectrum of IC2Cl-B(*R*)DMO-IC2Cl.

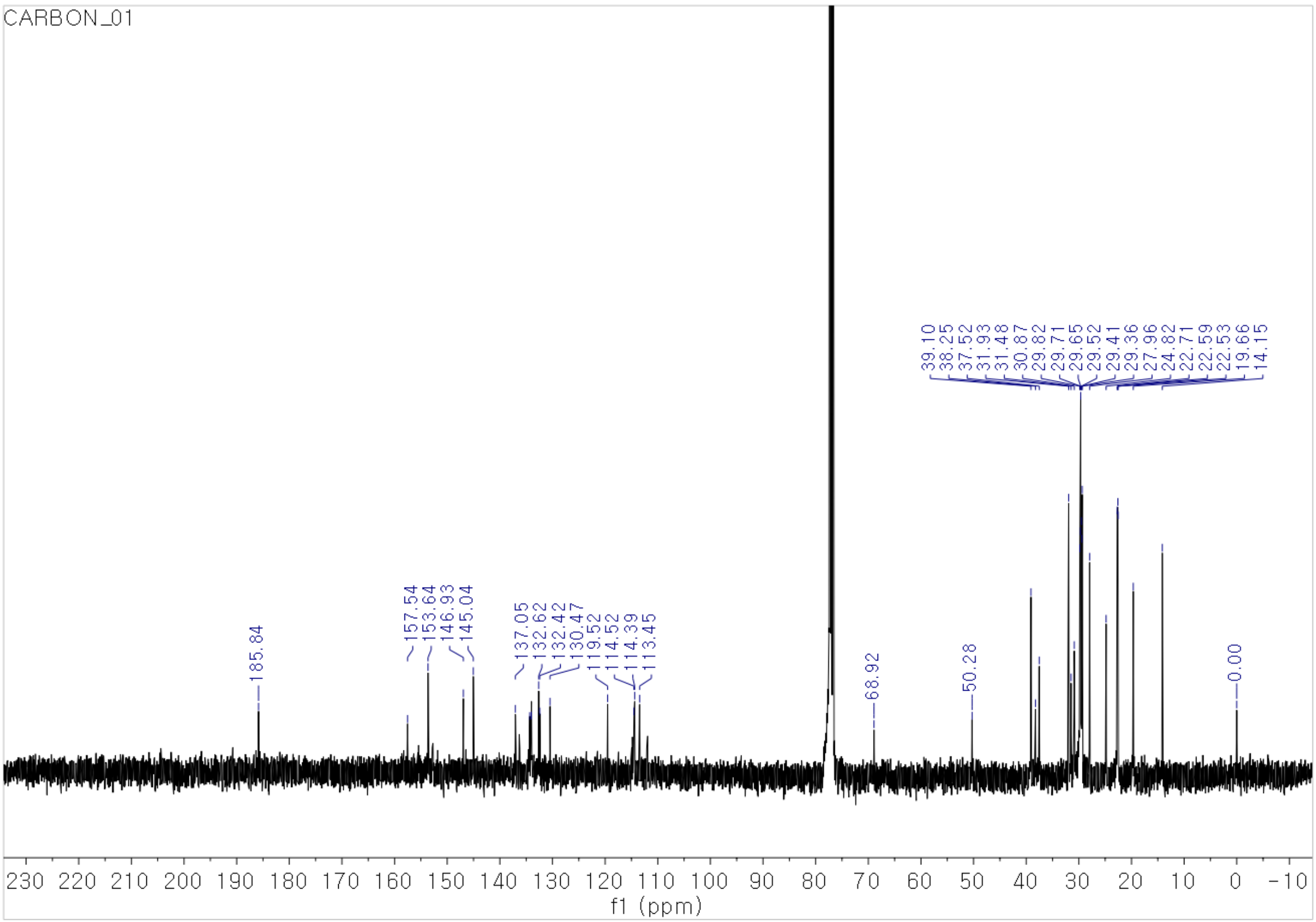

Figure S11. ^{13}C-NMR (100 MHz, $CDCl_3$) spectrum of IC2F-B(*S*)DMO-IC2F.

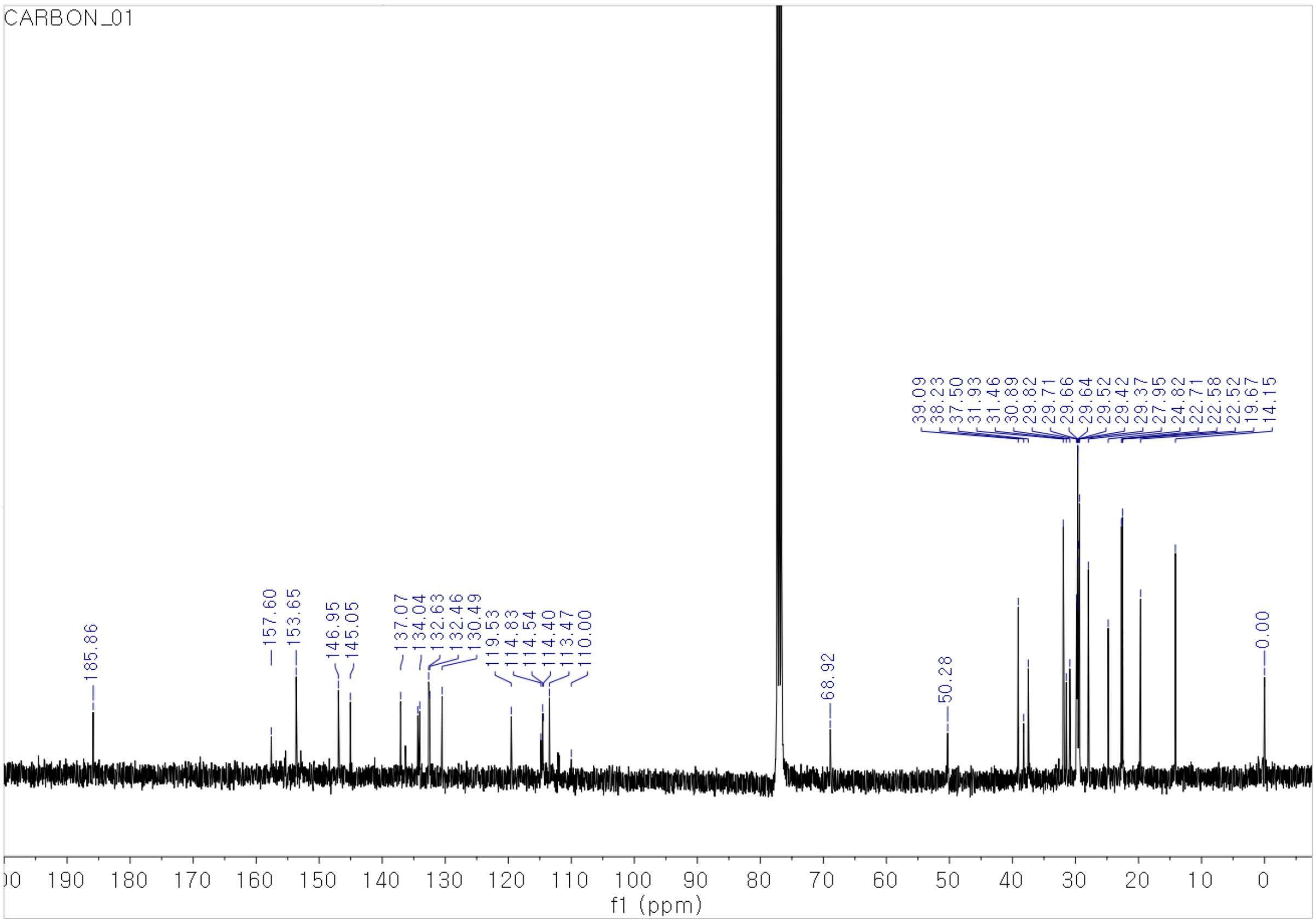

Figure S12. ^{13}C-NMR (100 MHz, $CDCl_3$) spectrum of IC2F-B(*R*)DMO-IC2F.

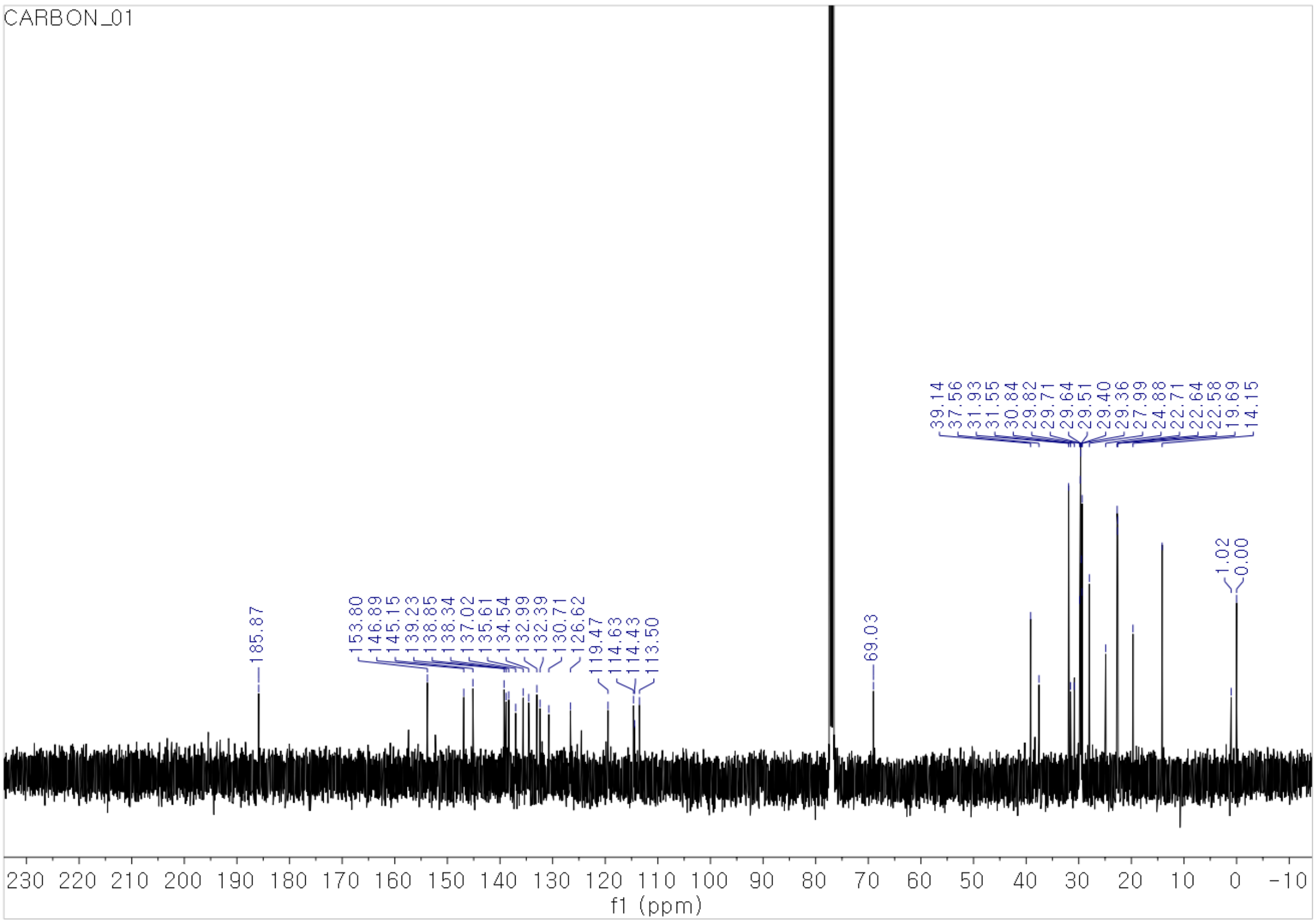

Figure S13. ^{13}C-NMR (100 MHz, $CDCl_3$) spectrum of IC2Cl-B(*S*)DMO-IC2Cl.

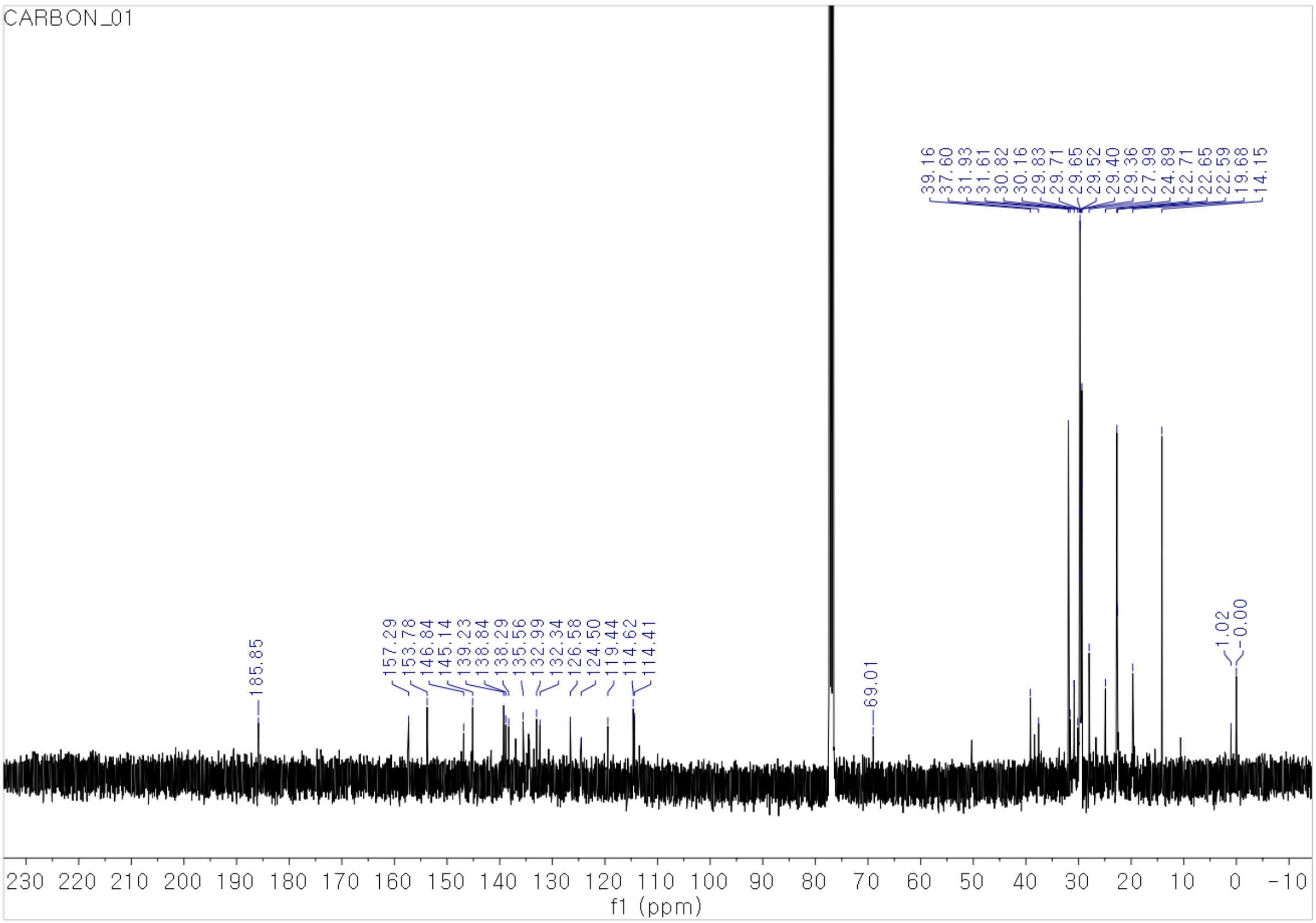

Figure S14. ^{13}C-NMR (100 MHz, $CDCl_3$) spectrum of IC2Cl-B(*R*)DMO-IC2Cl.

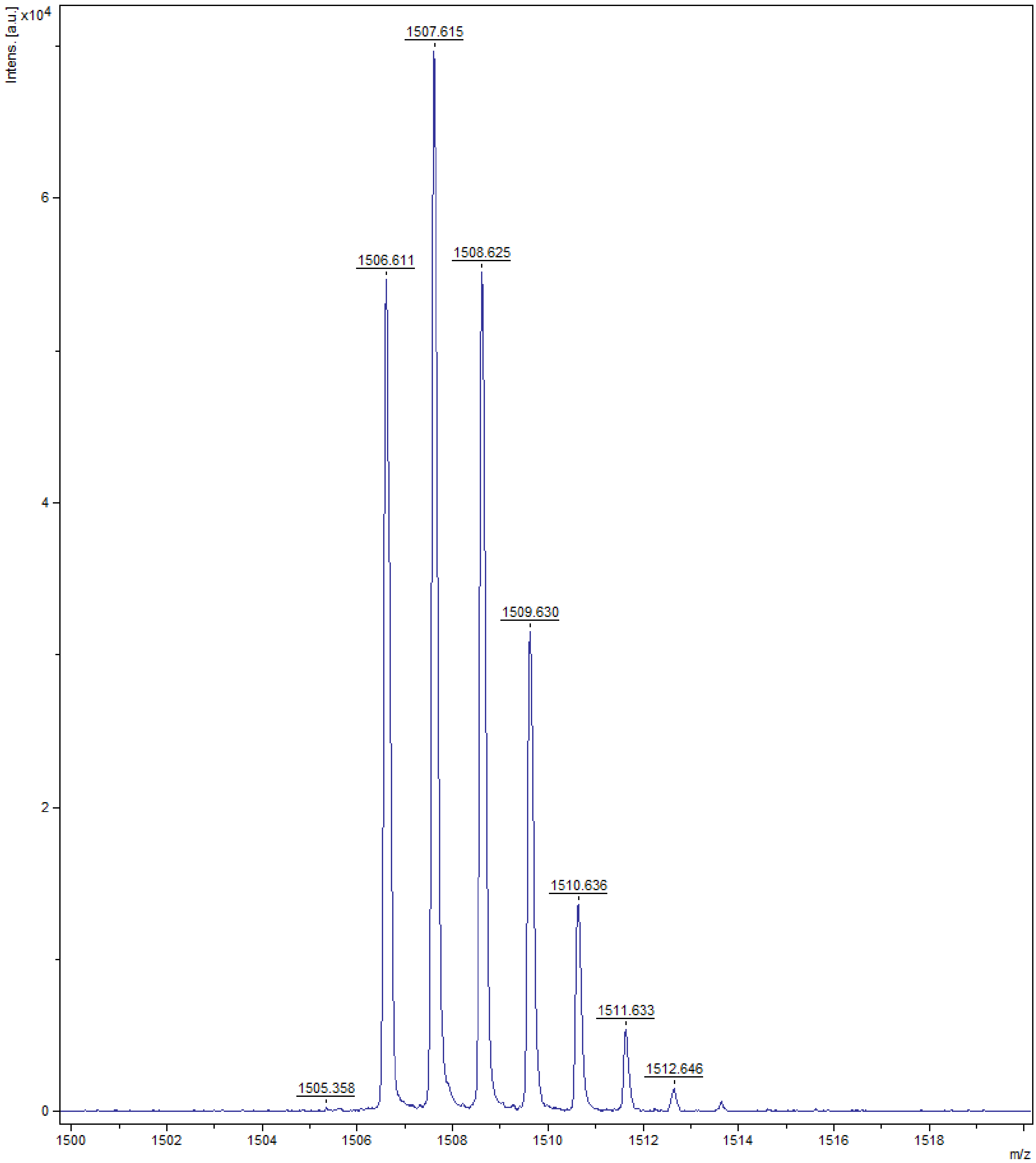

Figure S15. Mass spectrum of IC2F-B(*S*)DMO-IC2F by MALDI-TOF.

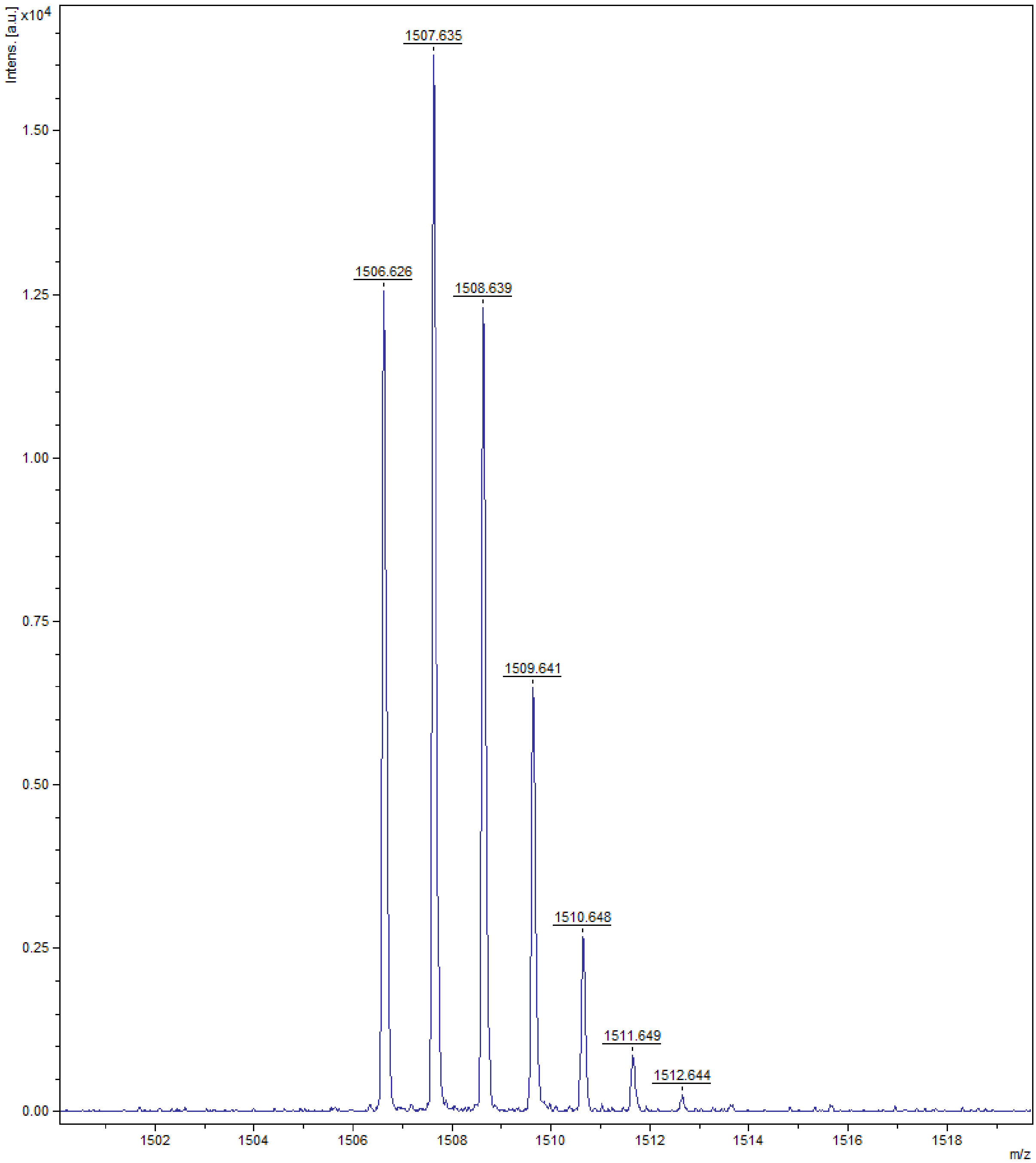

Figure S16. Mass spectrum of IC2F-B(*R*)DMO-IC2F by MALDI-TOF.

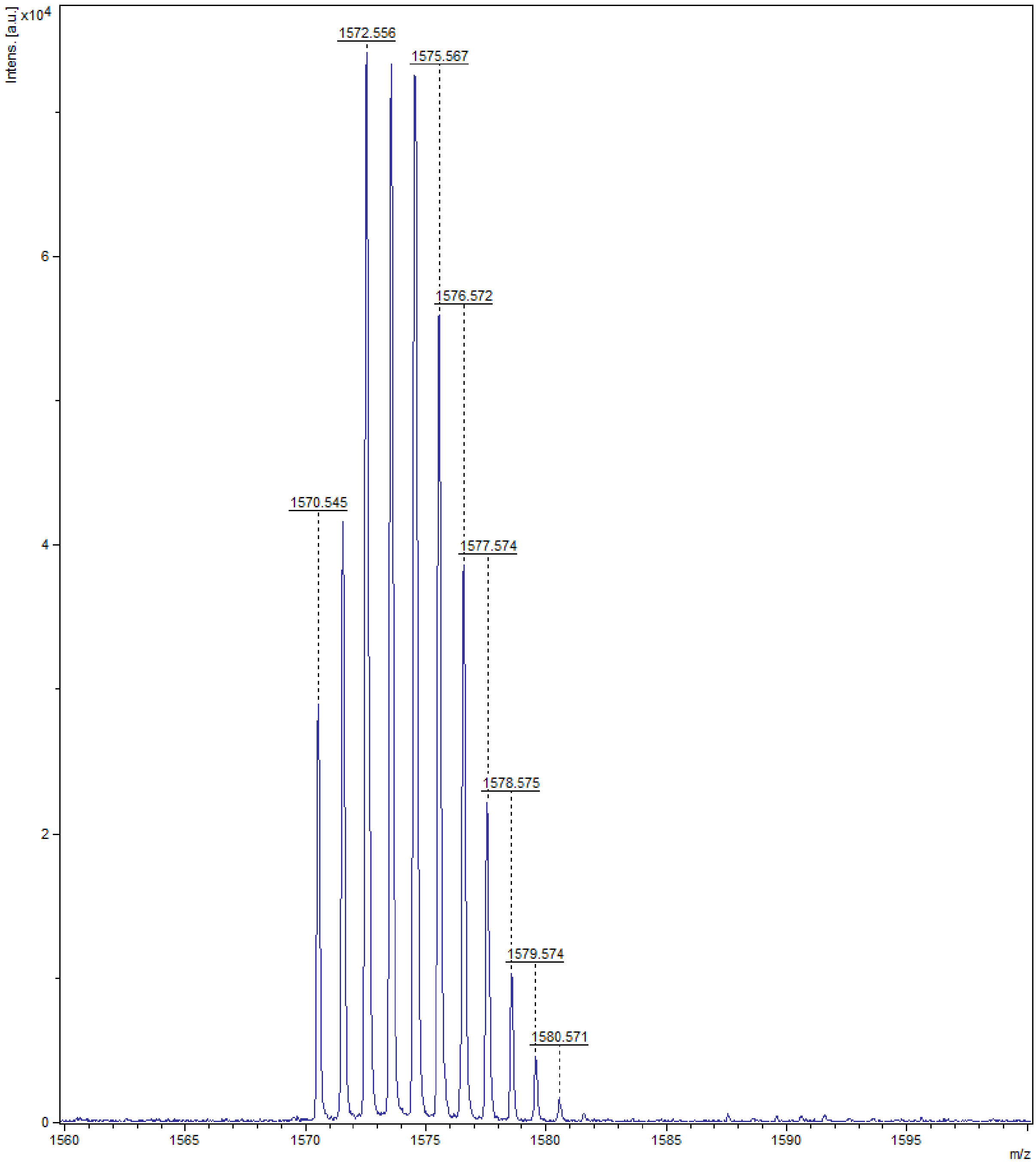

Figure S17. Mass spectrum of IC2Cl-B(*S*)DMO-IC2Cl by MALDI-TOF.

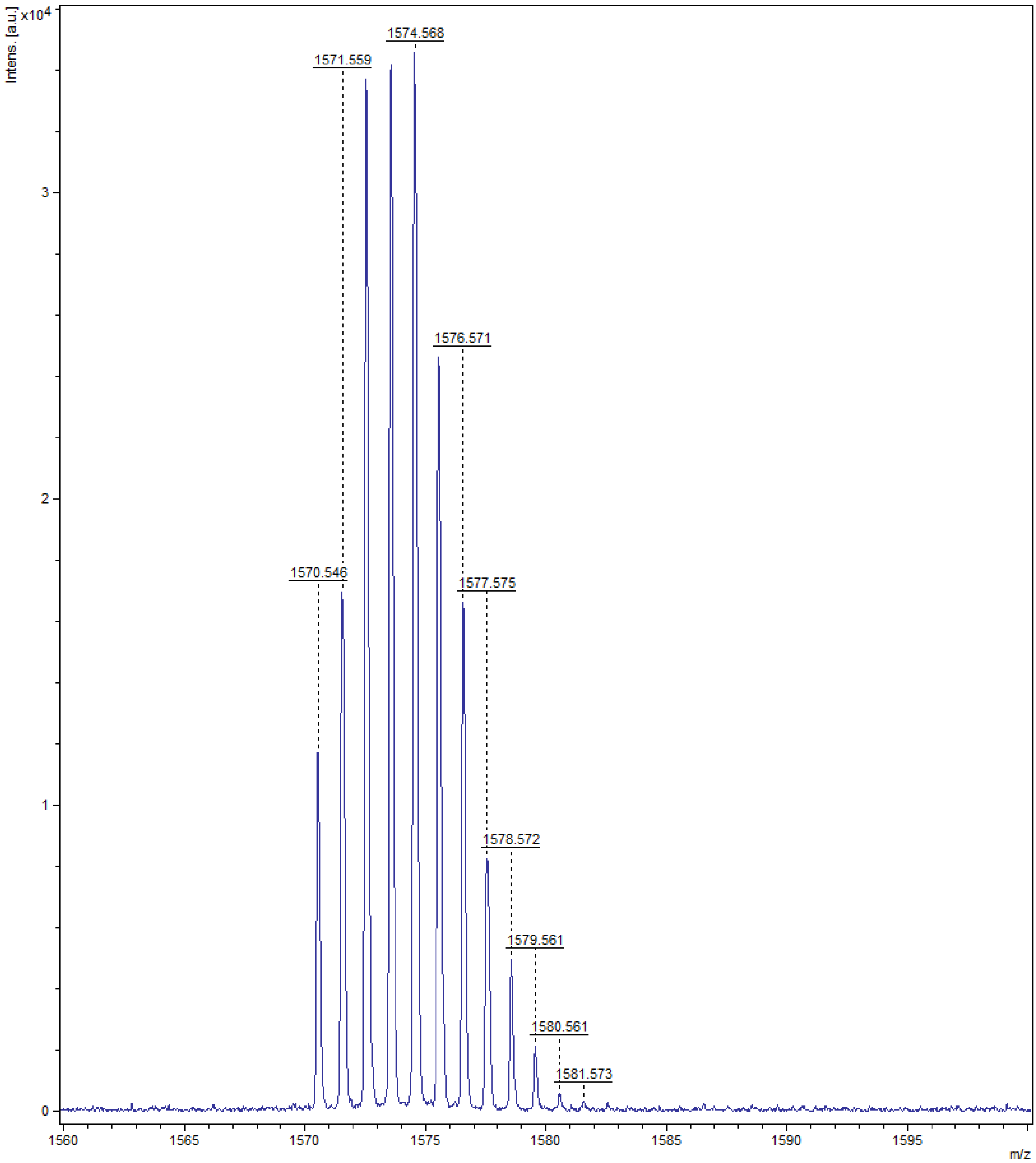

Figure S18. Mass spectrum of IC2Cl-B(*R*)DMO-IC2Cl by MALDI-TOF.

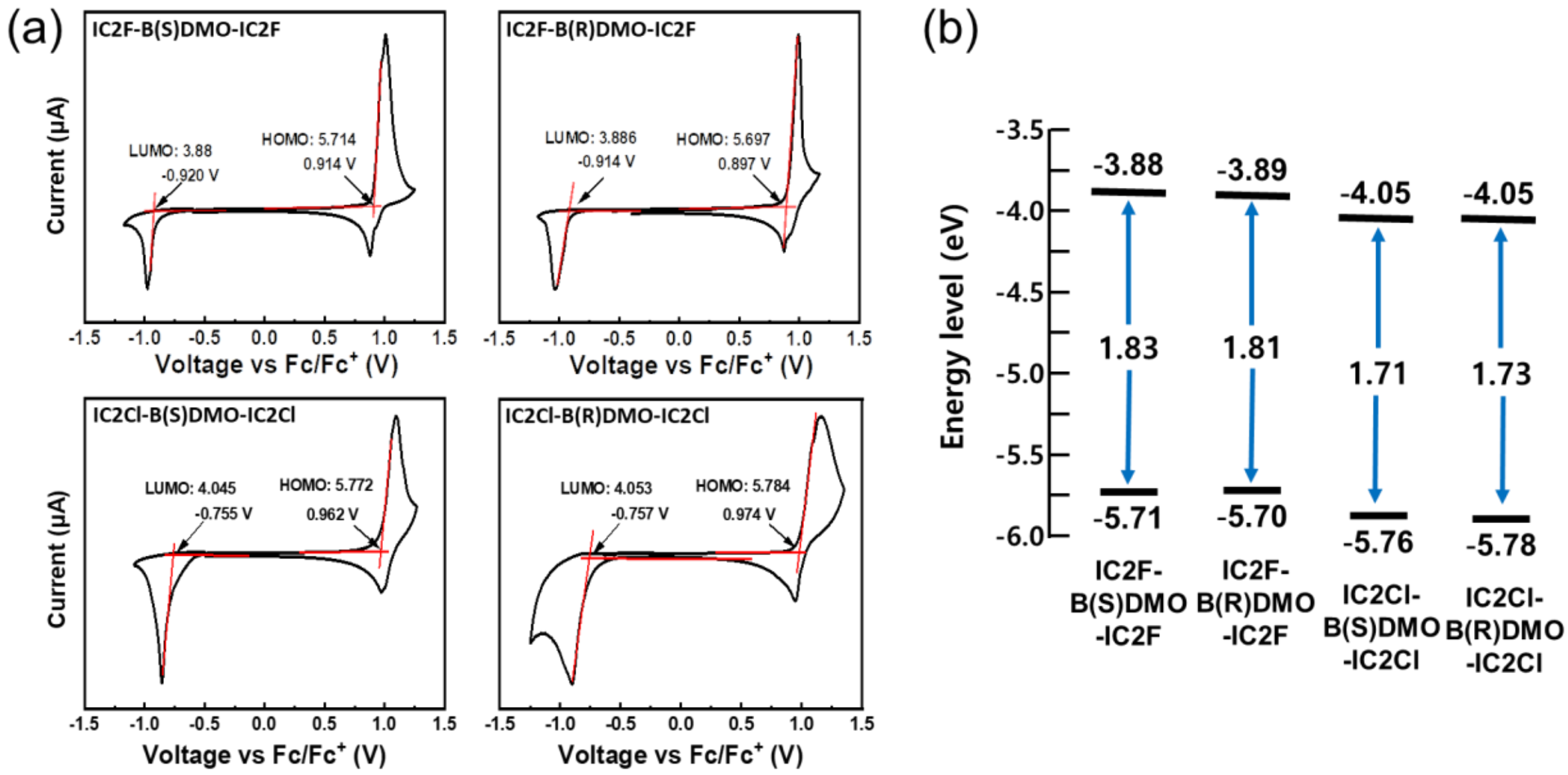

Figure S19. (a) The CV curves and energy levels of IC2F-B(*S*)DMO-IC2F, IC2F-B(*R*)DMO-IC2F, IC2Cl-B(*S*)DMO-IC2Cl, and IC2Cl-B(*R*)DMO-IC2Cl. (b) An energy level diagram of the chiral LFCs.

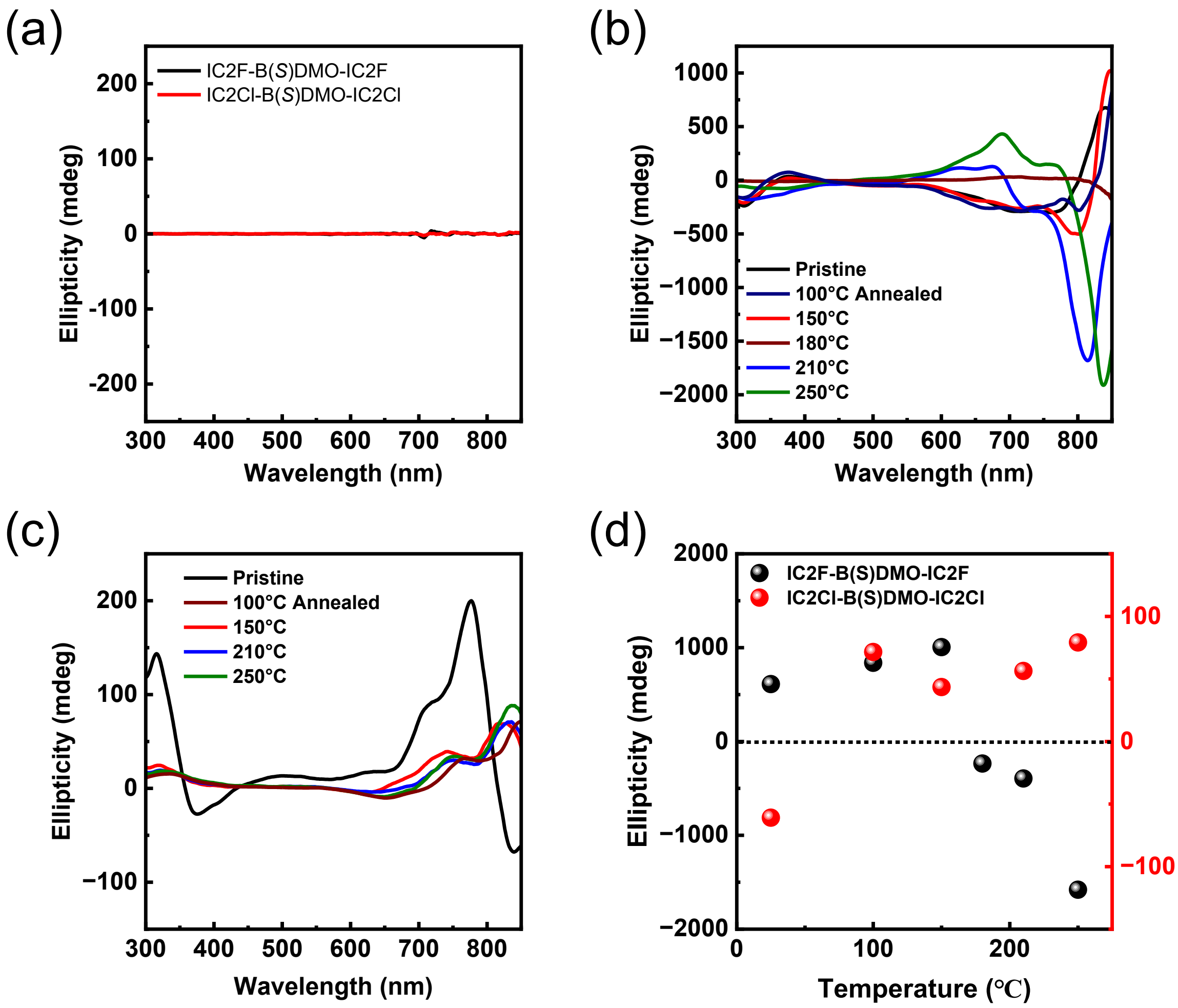

Figure S20. (a) CD spectra of IC2F-B(*S*)DMO-IC2F and IC2Cl-B(*S*)DMO-IC2Cl in solution (10^{-5} M in chloroform). (b, c) CD spectra of (b) IC2F-B(*S*)DMO-IC2F and (c) IC2Cl-B(*S*)DMO-IC2Cl thin films annealed at different temperatures. (d) Ellipticity of IC2F-B(*S*)DMO-IC2F and IC2Cl-B(*S*)DMO-IC2Cl thin films at 850 nm as a function of annealing temperatures.

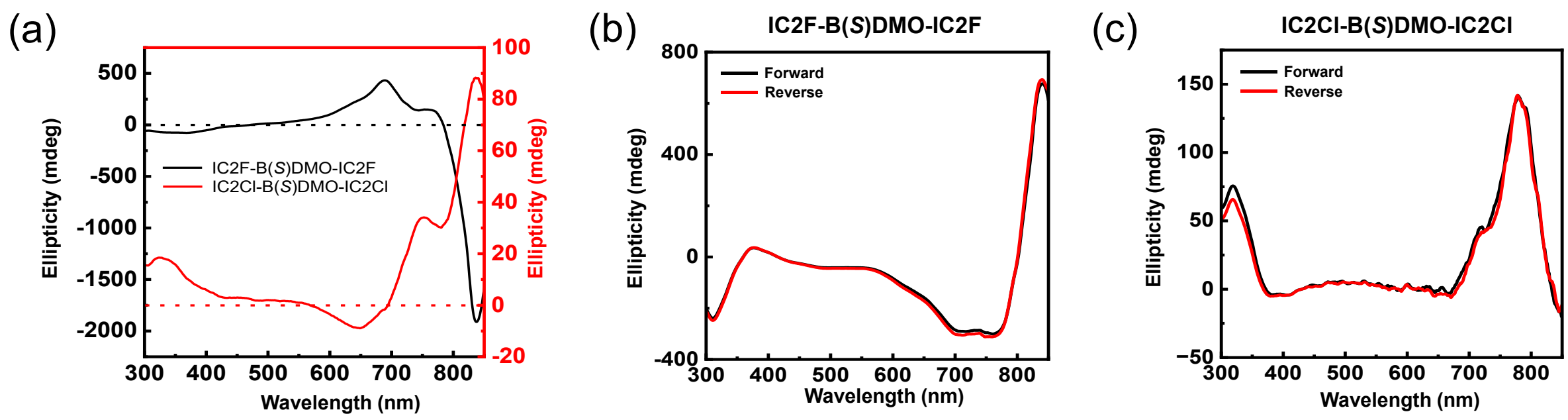

Figure S21. (a) CD spectra of IC2F-B(*S*)DMO-IC2F and IC2Cl-B(*S*)DMO-IC2Cl thin films annealed at 250 °C. (b, c) CD spectra of pristine (b) IC2F-B(*S*)DMO-IC2F and (c) IC2Cl-B(*S*)DMO-IC2Cl thin films under forward and backward light incidence.

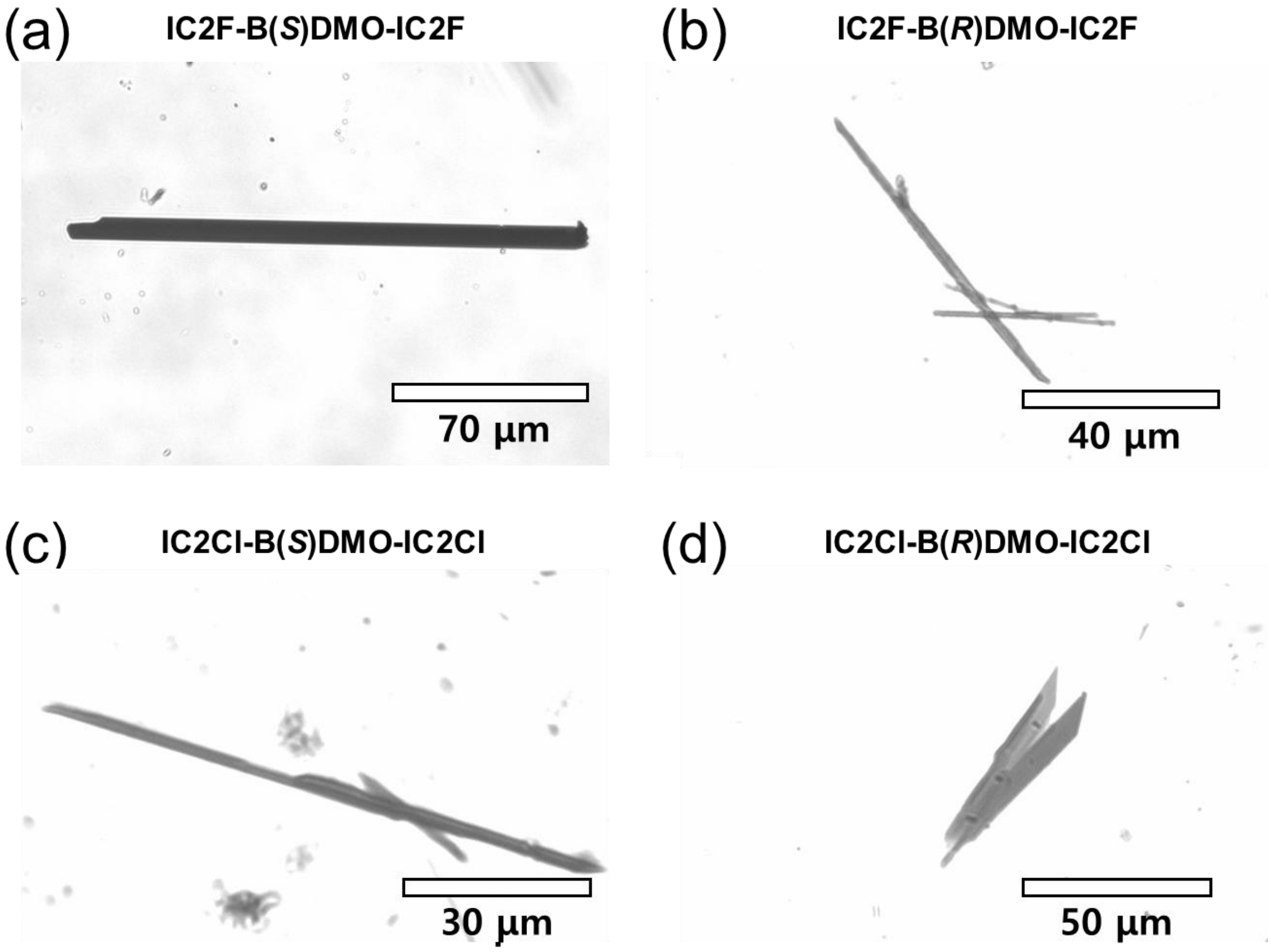

Figure S22. Optical microscope images of (a) IC2F-B(*S*)DMO-IC2F, (b) IC2F-B(*R*)DMO-IC2F, (c) IC2Cl-B(*S*)DMO-IC2Cl, and (d) IC2Cl-B(*R*)DMO-IC2Cl single-crystals.

Table S1. Crystallographic data of chiral LFCs.

Compound name	IC2F-B(*S*)DMO-IC2F	IC2F-B(*R*)DMO-IC2F	IC2Cl-B(*S*)DMO-IC2Cl	IC2Cl-B(*R*)DMO-IC2Cl
Formular	$C_{74}H_{94}N_8O_2S_5F_4$	$C_{74}H_{94}N_8O_2S_5F_4$	$C_{74}H_{94}N_8O_2S_5Cl_4$	$C_{74}H_{94}N_8O_2S_5Cl_4$
Crystal system, space group	Triclinic, P-1	Triclinic, P-1	Triclinic, P-1	Triclinic, P-1
Temperature (K)	100 K	100 K	100 K	100 K
Cell Lengths (Å)	a = 8.3370 (17)<br>b = 18.477 (4)<br>c = 25.725 (5)	a = 8.3170 (17)<br>b = 18.439 (4)<br>c = 25.735 (5)	a = 8.3510 (17)<br>b = 18.822 (4)<br>c = 25.749 (5)	a = 8.3420 (17)<br>b = 18.603 (4)<br>c = 25.698 (5)
Cell Angles (°)	α = 100.77 (3)<br>β = 96.22 (3)<br>γ = 100.02 (3)	α = 101.02 (3)<br>β = 96.04 (3)<br>γ = 100.06 (3)	α = 99.48 (3)<br>β = 97.23 (3)<br>γ = 99.91 (3)	α = 100.03 (3)<br>β = 96.57 (3)<br>γ = 100.13 (3)
Cell Volume ($Å^3$)	3792.6	3774.8	3882.7	3822.3
Z, Z'	Z: 2, Z': 0	Z: 2, Z': 0	Z: 2, Z': 0	Z: 2, Z': 0

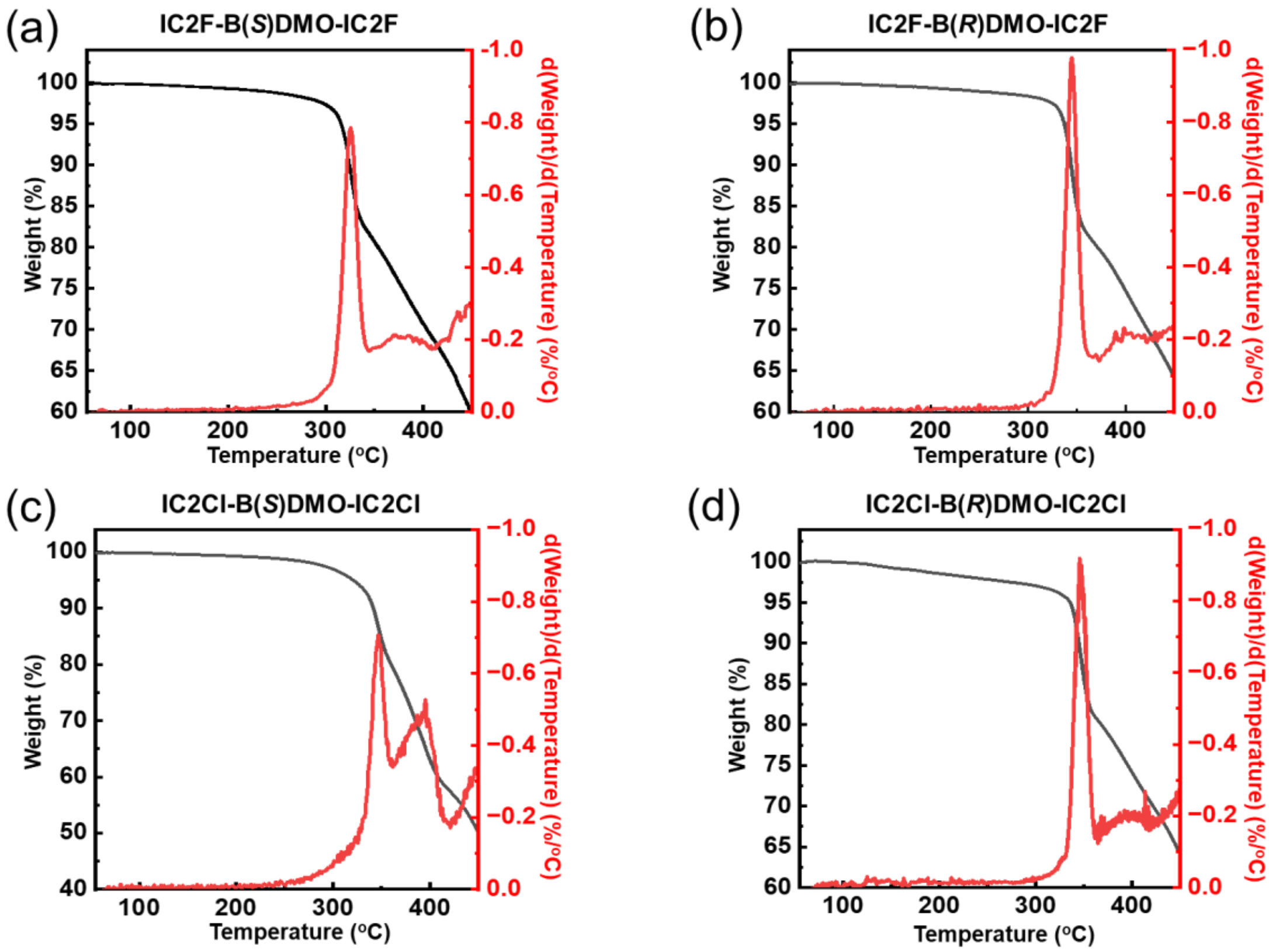

Figure S23. TGA curves of (a) IC2F-B(*S*)DMO-IC2F, (b) IC2F-B(*R*)DMO-IC2F, (c) IC2Cl-B(*S*)DMO-IC2Cl, and (d) IC2Cl-B(*R*)DMO-IC2Cl with a heating rate of 10 °C/min under nitrogen atmosphere.

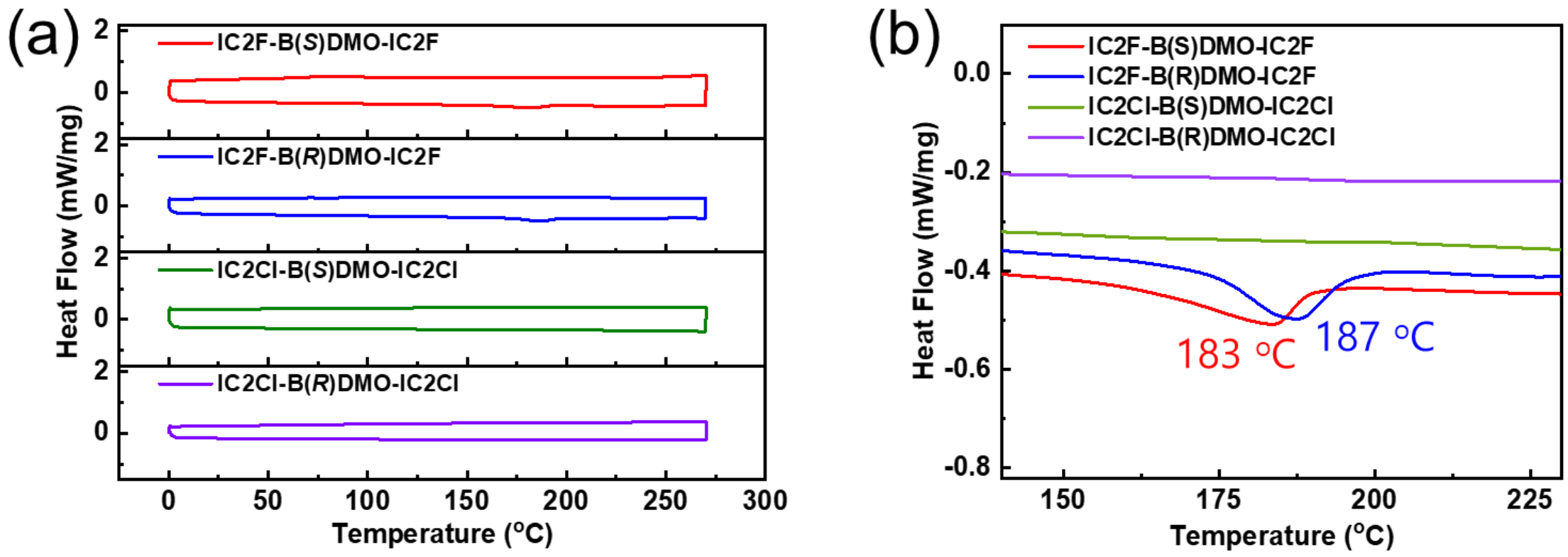

Figure S24. (a) DSC curves of IC2F-B(*S*)DMO-IC2F, IC2F-B(*R*)DMO-IC2F, IC2Cl-B(*S*)DMO-IC2Cl, and IC2Cl-B(*R*)DMO-IC2Cl with a heating rate of 10 °C/min under nitrogen atmosphere. (b) The enlargement of the DSC curve of the LFCs around the glass transition temperature

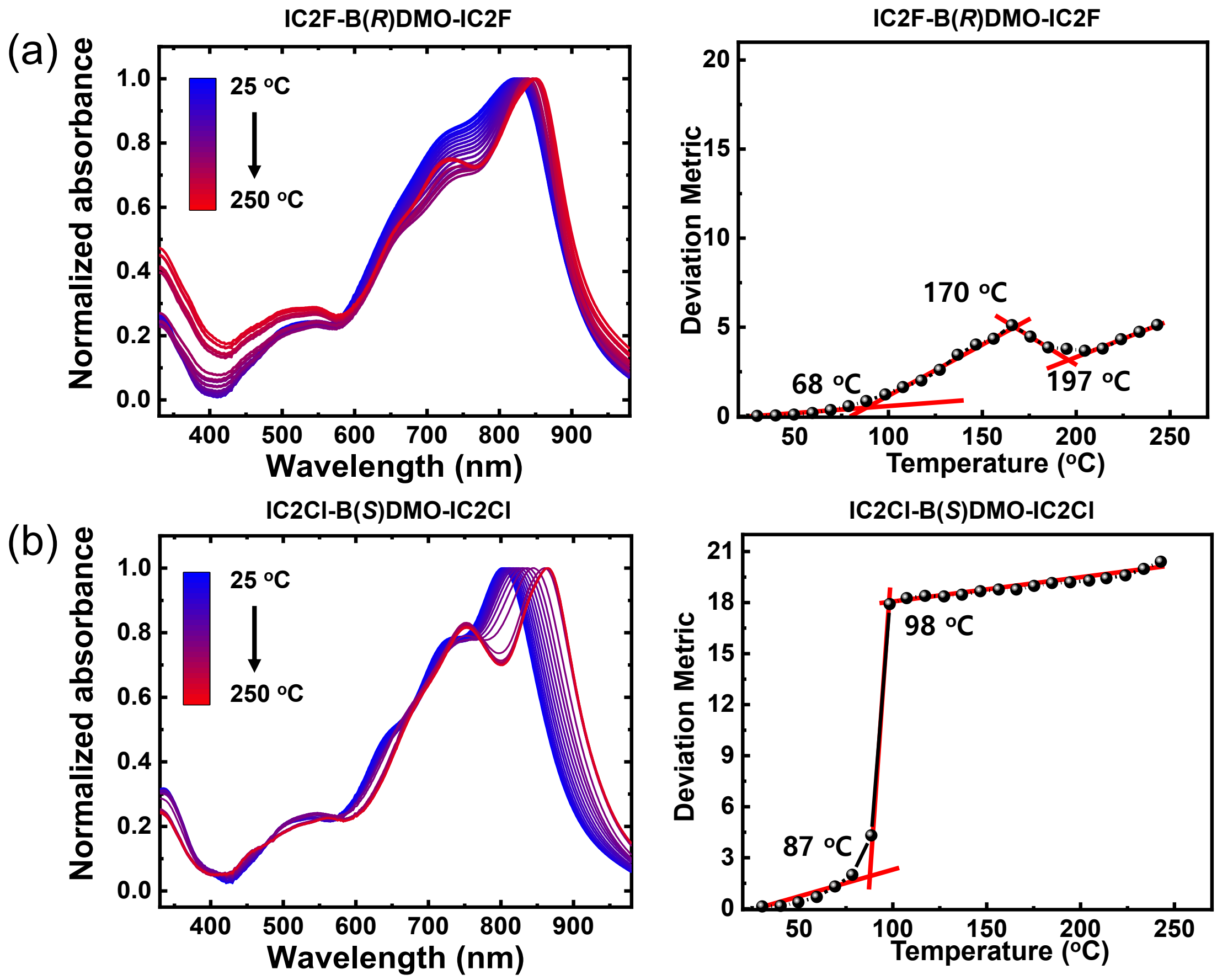

Figure S25. Temperature-dependent UV-vis absorption deviation metric plots for (a) IC2F-B(*R*)DMO-IC2F and (b) IC2Cl-B(*R*)DMO-IC2Cl thin films.

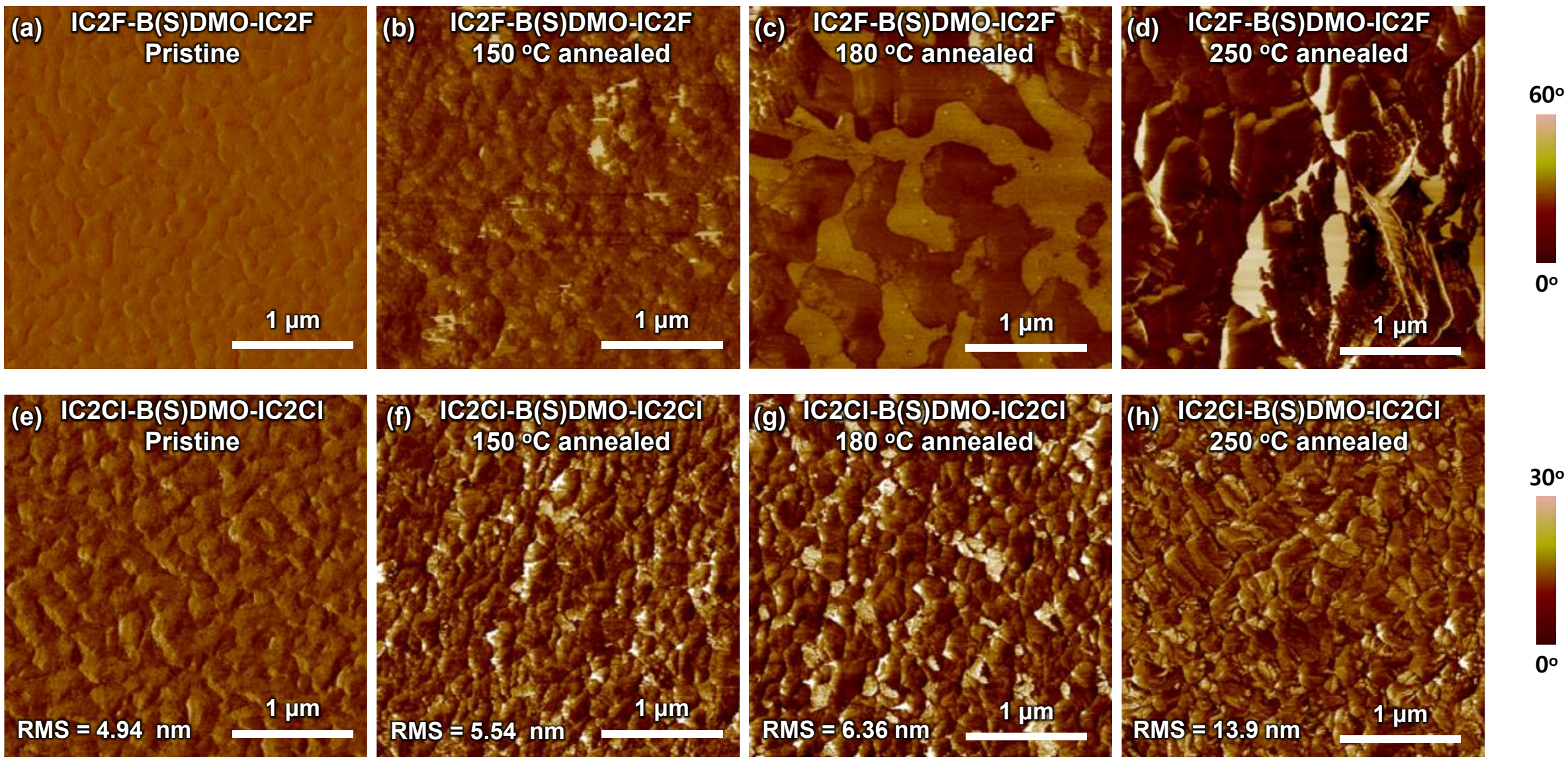

Figure S26. AFM phase images of (a-d) IC2F-B(*S*)DMO-IC2F and (e-h) IC2Cl-B(*S*)DMO-IC2Cl thin films under different annealing temperatures: (a, e) Pristine, (b, f) 150 °C, (c, g) 180 °C, and (d, h) 250 °C.

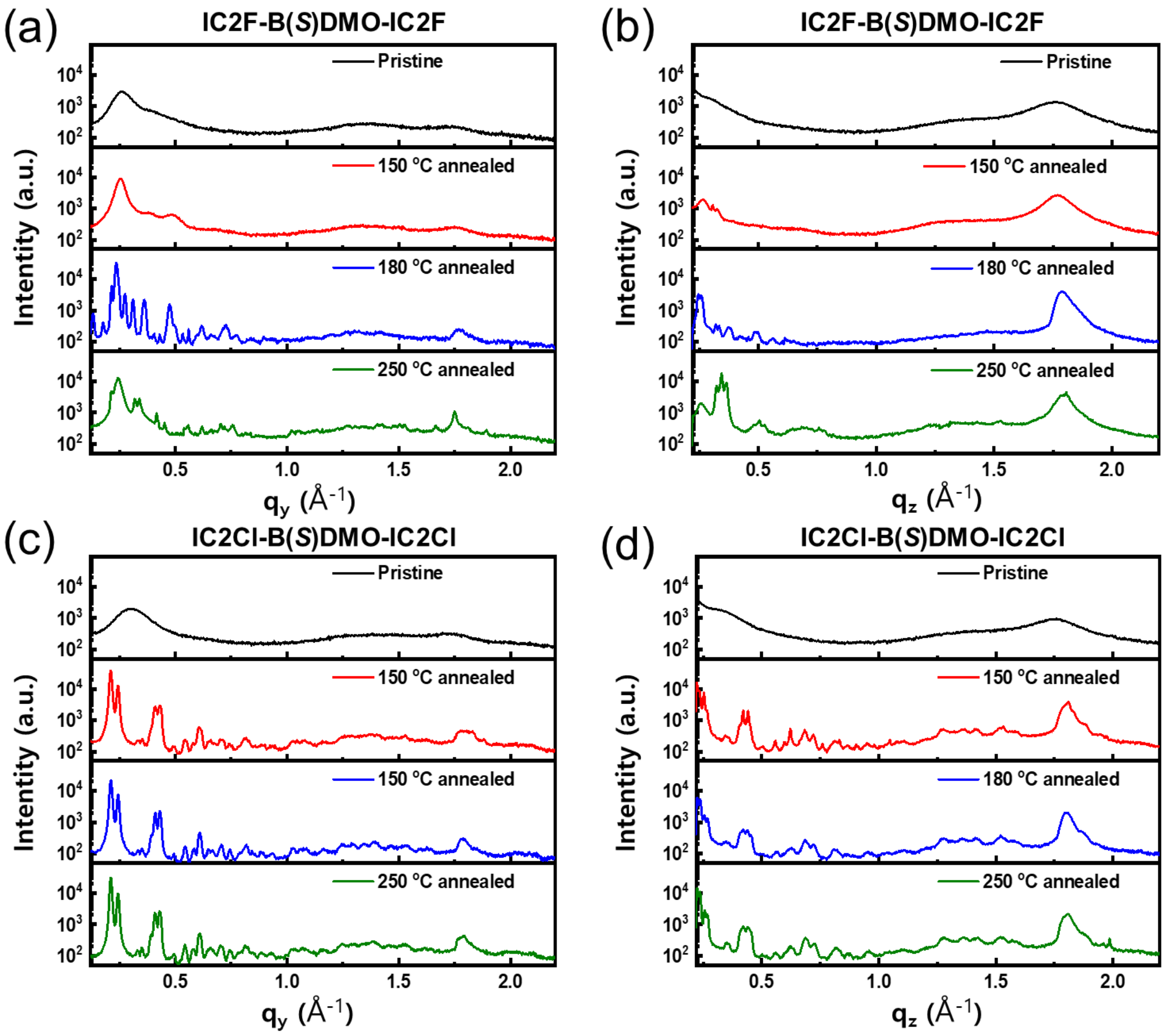

Figure S27. Line-cut profiles of the GIWAXS images for (a, b) IC2F-B(*S*)DMO-IC2F and (c, d) IC2Cl-B(*S*)DMO-IC2Cl films in the (a, c) q_y and (b, d) q_z directions.

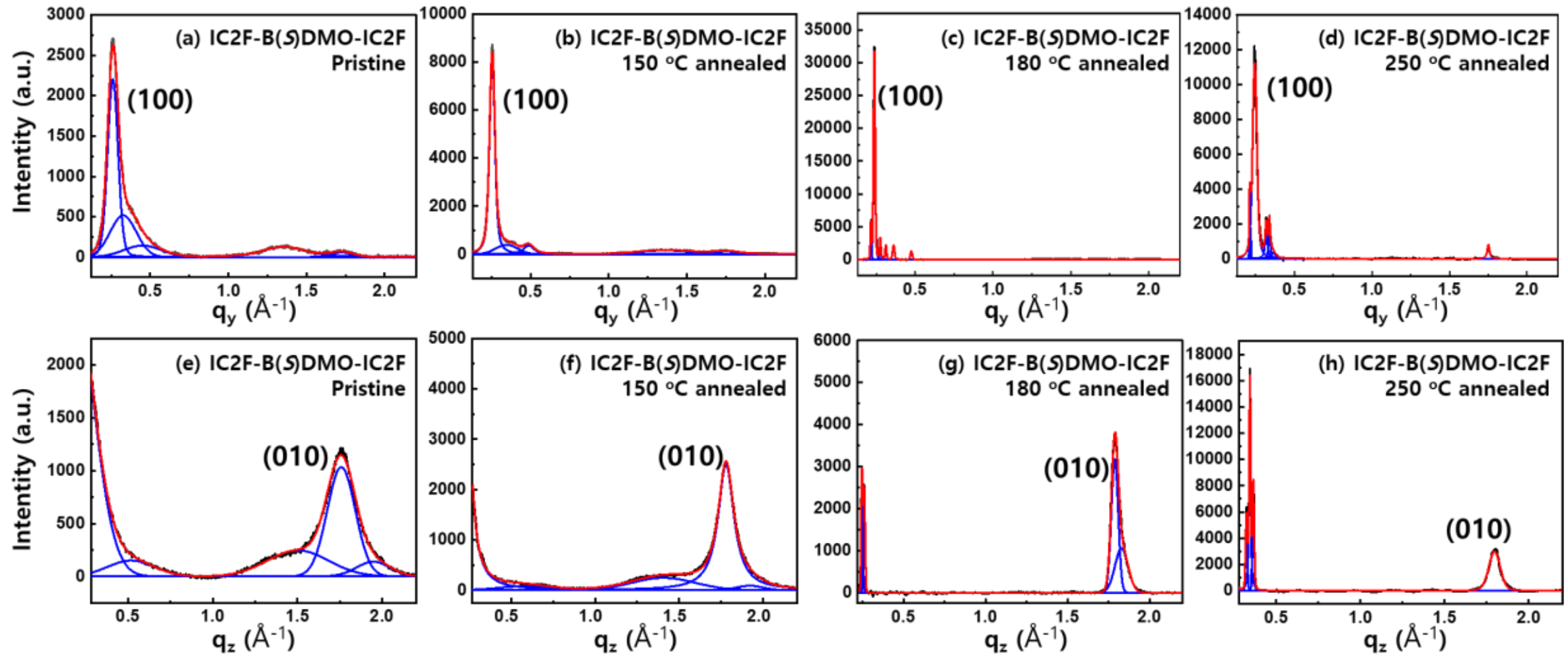

Figure S28. The fitting of the line-cut profiles of the GIWAXS images for IC2F-B(*S*)MDO-IC2F films in the (a-d) q_y and (e-h) q_z directions under different annealing temperatures: (a, e) Pristine, (b, f) 150 °C, (c, g) 180 °C, and (d, h) 250 °C.

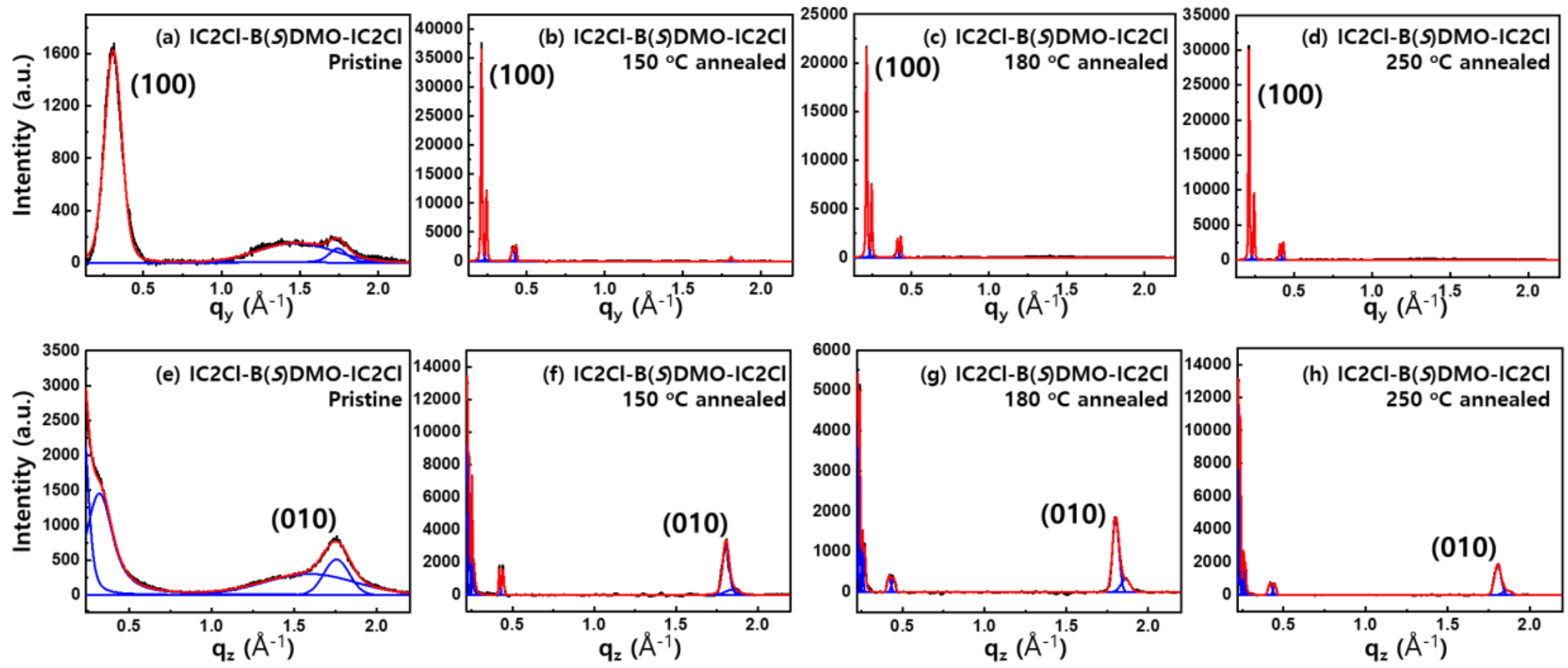

Figure S29. The fitting of the line-cut profiles of the GIWAXS images for IC2Cl-B(*S*)MDO-IC2Cl films in the (a-d) q_y and (e-h) q_z directions under different annealing temperatures: (a, e) Pristine, (b, f) 150 °C, (c, g) 180 °C, and (d, h) 250 °C.

Table S2. Summary of GIWAXS parameters of IC2F-B(*S*)DMO-IC2F and IC2Cl-B(*S*)DMO-IC2Cl films under different annealing temperatures.

Sample	Lamellar spacing from (100) peak in a q_y direction						π-π spacing from (010) peak in the q_z direction					
	q (Å^{-1})	*d* (Å)	Height (a.u.)	Area (a.u.)	FWHM (Å^{-1})	CCL (nm)	*q* (Å^{-1})	*d* (Å)	Height (a.u.)	Area (a.u.)	FWHM (Å^{-1})	CCL (nm)
IC2F-B(*S*)DMO-IC2F Pristine	0.26	24.11	2205.80	194.30	0.07	8.43	1.76	3.57	1033.59	215.26	0.20	2.89
IC2F-B(*S*)DMO-IC2F 150 ºC annealed	0.25	24.76	8244.38	441.61	0.04	13.66	1.78	3.53	2512.41	445.96	0.11	5.00
IC2F-B(*S*)DMO-IC2F 180 ºC annealed	0.24	26.55	31881.50	471.00	0.01	44.21	1.79	3.51	3178.63	166.00	0.05	11.53
IC2F-B(*S*)DMO-IC2F 250 ºC annealed	0.25	25.68	11147.00	464.08	0.02	28.73	1.80	3.50	2988.53	275.51	0.06	8.88
IC2Cl-B(*S*)DMO-IC2Cl Pristine	0.31	20.42	1628.07	239.83	0.13	4.38	1.76	3.58	518.27	102.22	0.19	3.05
IC2Cl-B(*S*)DMO-IC2Cl 150 ºC annealed	0.21	29.77	36800.40	471.78	0.01	52.16	1.81	3.48	3159.67	168.94	0.05	12.36
IC2Cl-B(*S*)DMO-IC2Cl 180 ºC annealed	0.21	29.70	21792.10	277.10	0.01	54.83	1.80	3.49	1829.45	90.61	0.05	12.15
IC2Cl-B(*S*)DMO-IC2Cl 250 ºC annealed	0.21	29.74	30625.50	340.08	0.01	58.85	1.80	3.48	1846.85	91.28	0.05	12.18

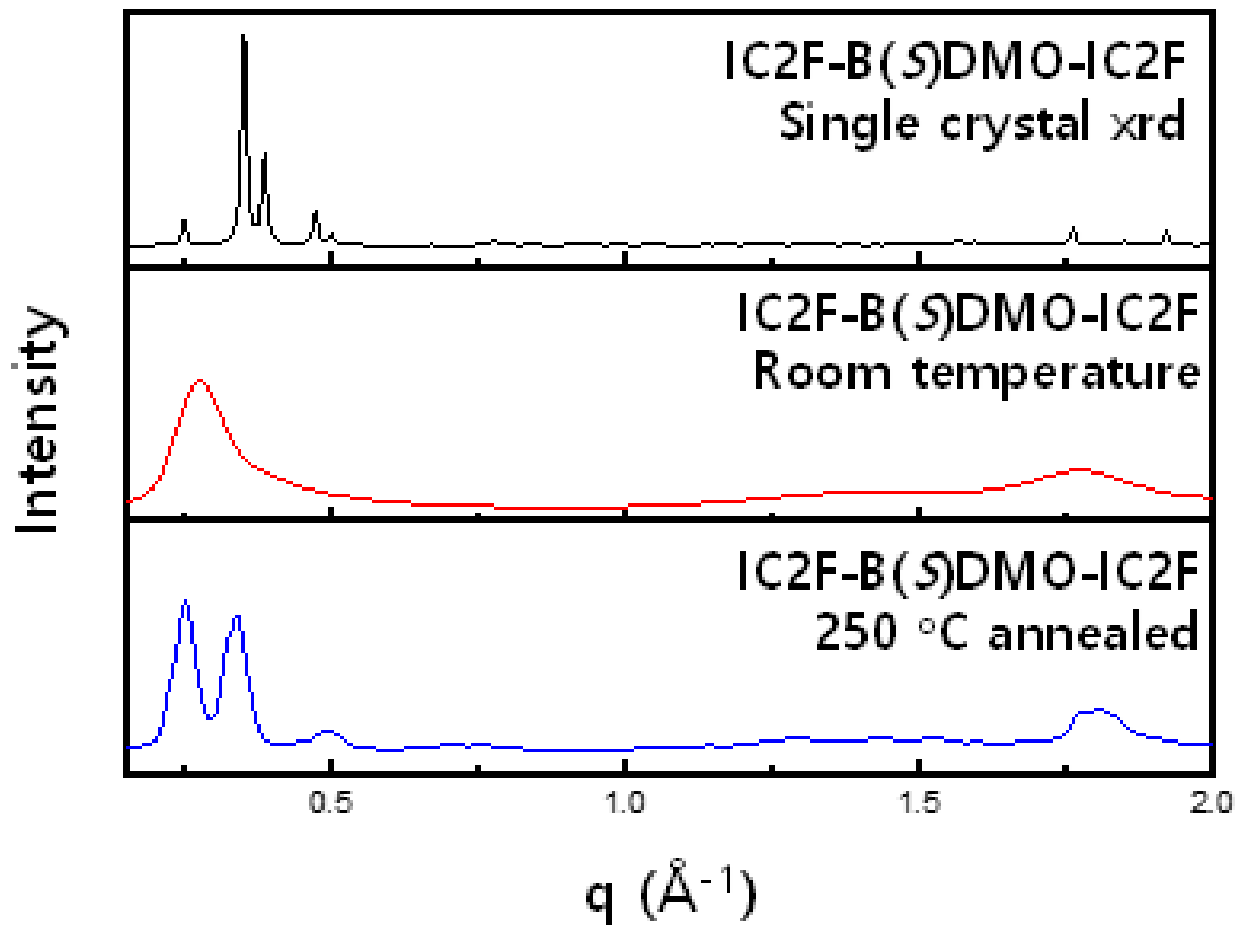

Figure S30. Comparison of the simulated PXRD profile derived from single crystal structure and GIWAXS profile of IC2F-B(*S*)DMO-IC2F thin films.

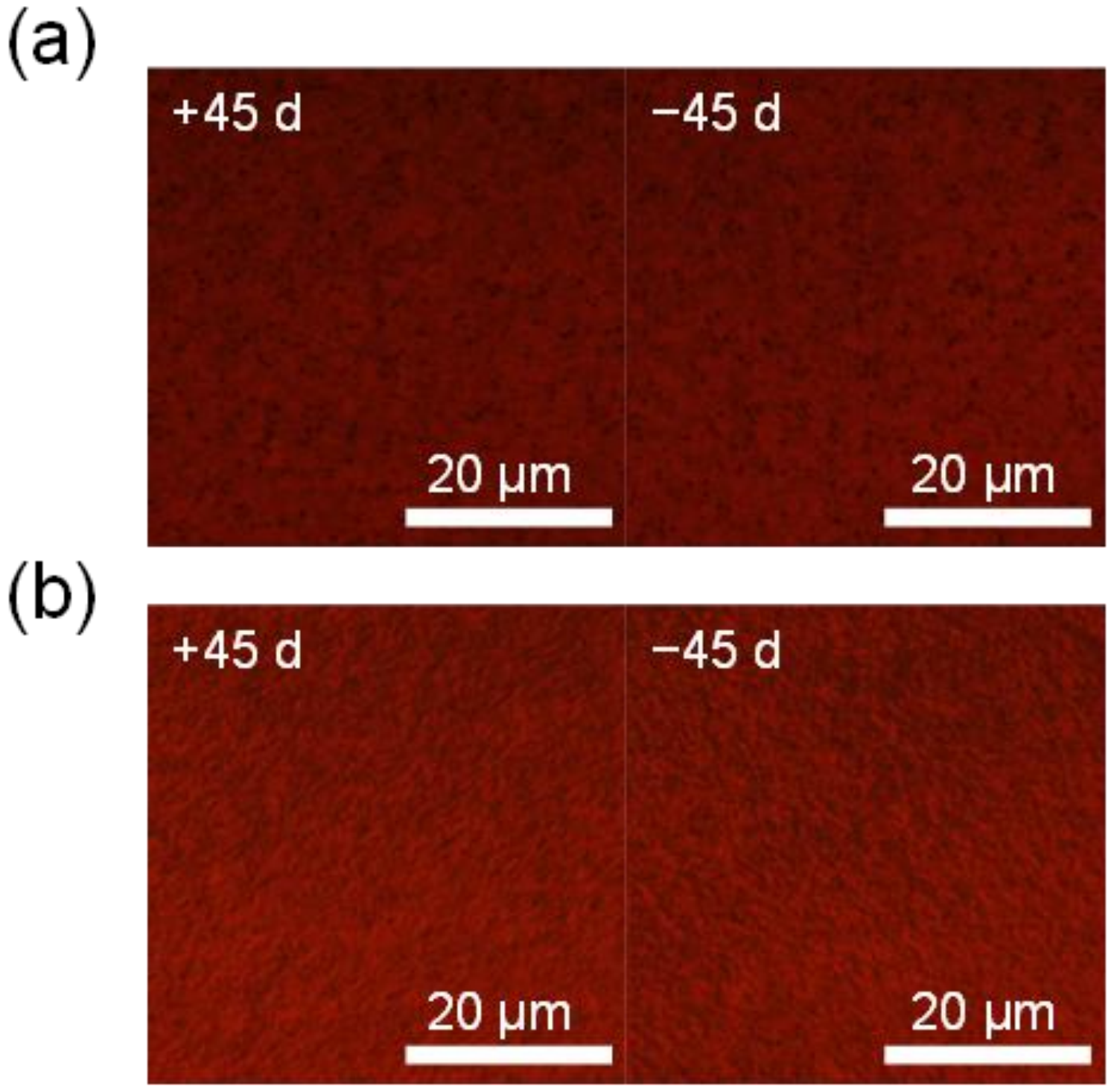

Figure S31. Polarized optical microscope images of (a) IC2F-B(*S*)DMO-IC2F and (b) IC2Cl-B(*S*)DMO-IC2Cl thin films with the angle between two polarizers of ±45°.

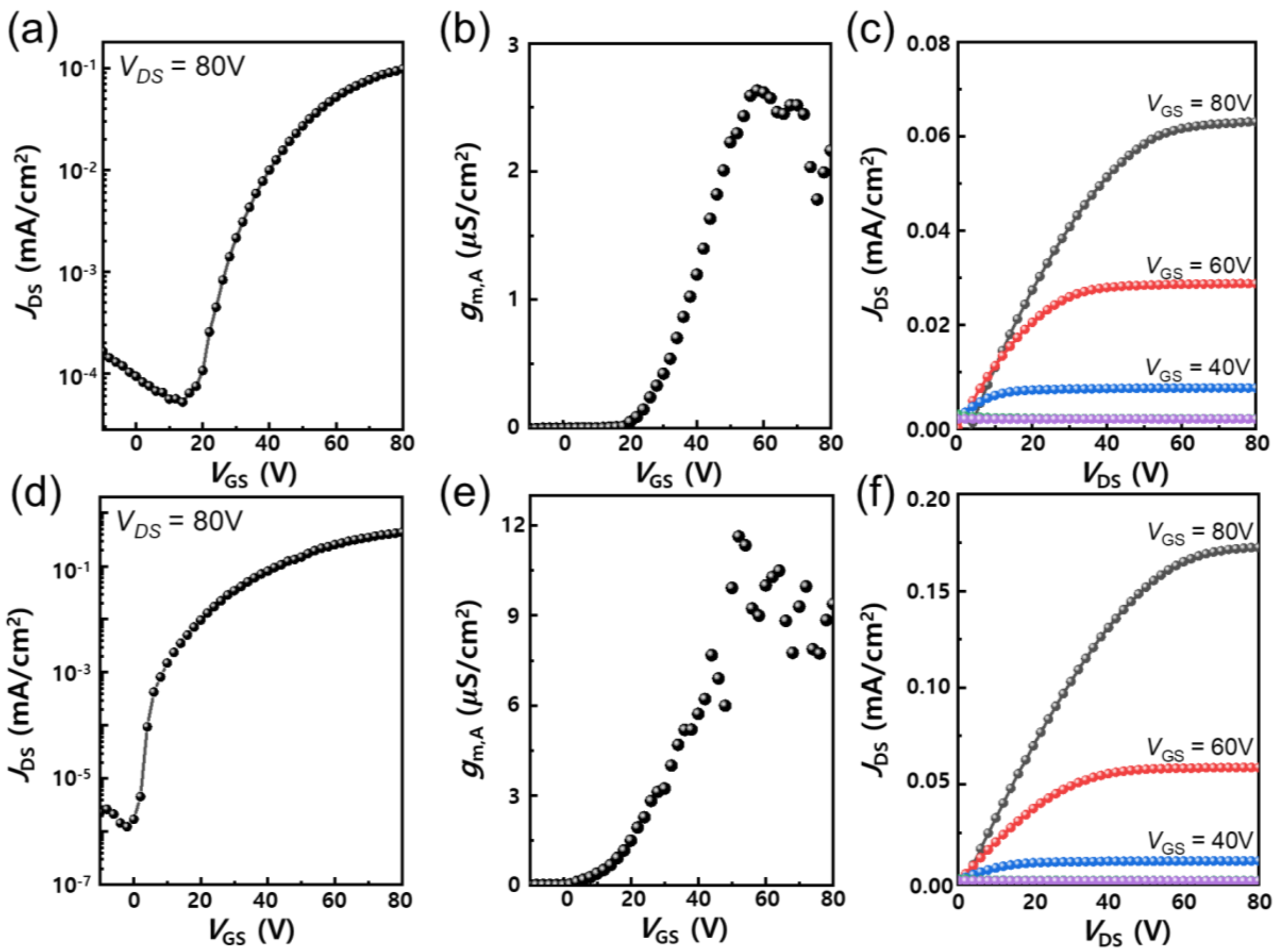

Figure S32. (a) Transfer characteristics, (b) specific transconductance, and (c) output characteristics of SB-VOFETs based on IC2F-B(*R*)DMO-IC2F thin films annealed at 230 °C under dark and vacuum conditions. (d) Transfer characteristics, (e) specific transconductance, and (f) output characteristics of SB-VOFETs based on IC2Cl-B(*R*)DMO-IC2Cl thin films annealed at 230 °C under dark and vacuum conditions.

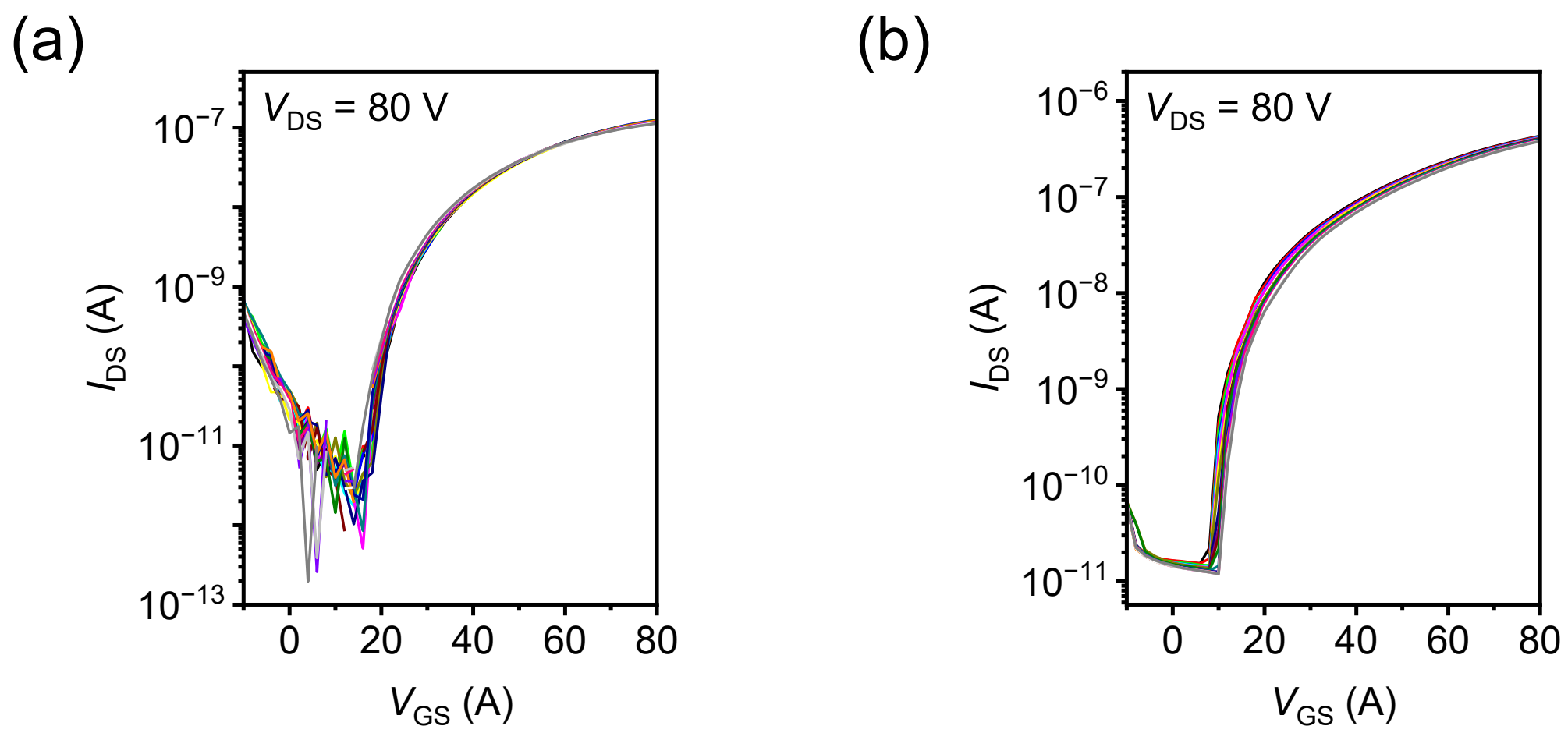

Figure S33. Transfer characteristics of SB-VOFETs based on (a) IC2F-B(*S*)DMO-IC2F thin films and (b) IC2Cl-B(*S*)DMO-IC2Cl thin films annealed at 230 °C, measured over 20 consecutive cycles under dark and vacuum conditions.

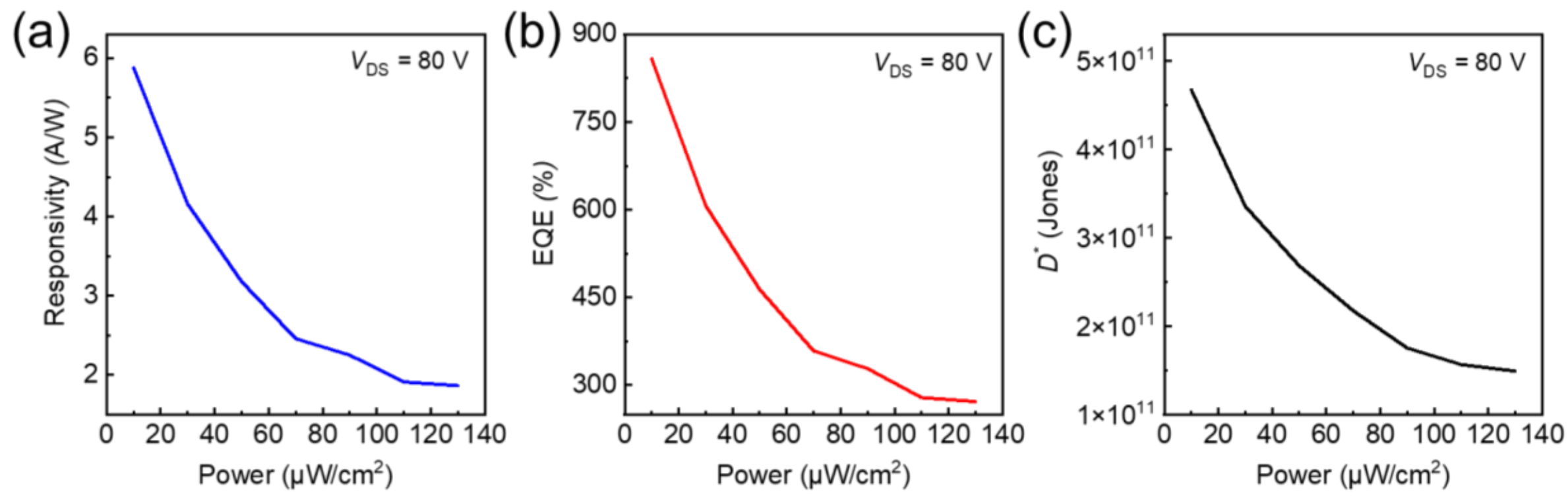

Figure S34. Maximum (a) R, (b) EQE, and (c) D^* values of SB-VOFETs based on IC2F-B(*S*)DMO-IC2F thin films annealed at 230 °C under varying illumination intensities (λ = 850 nm).

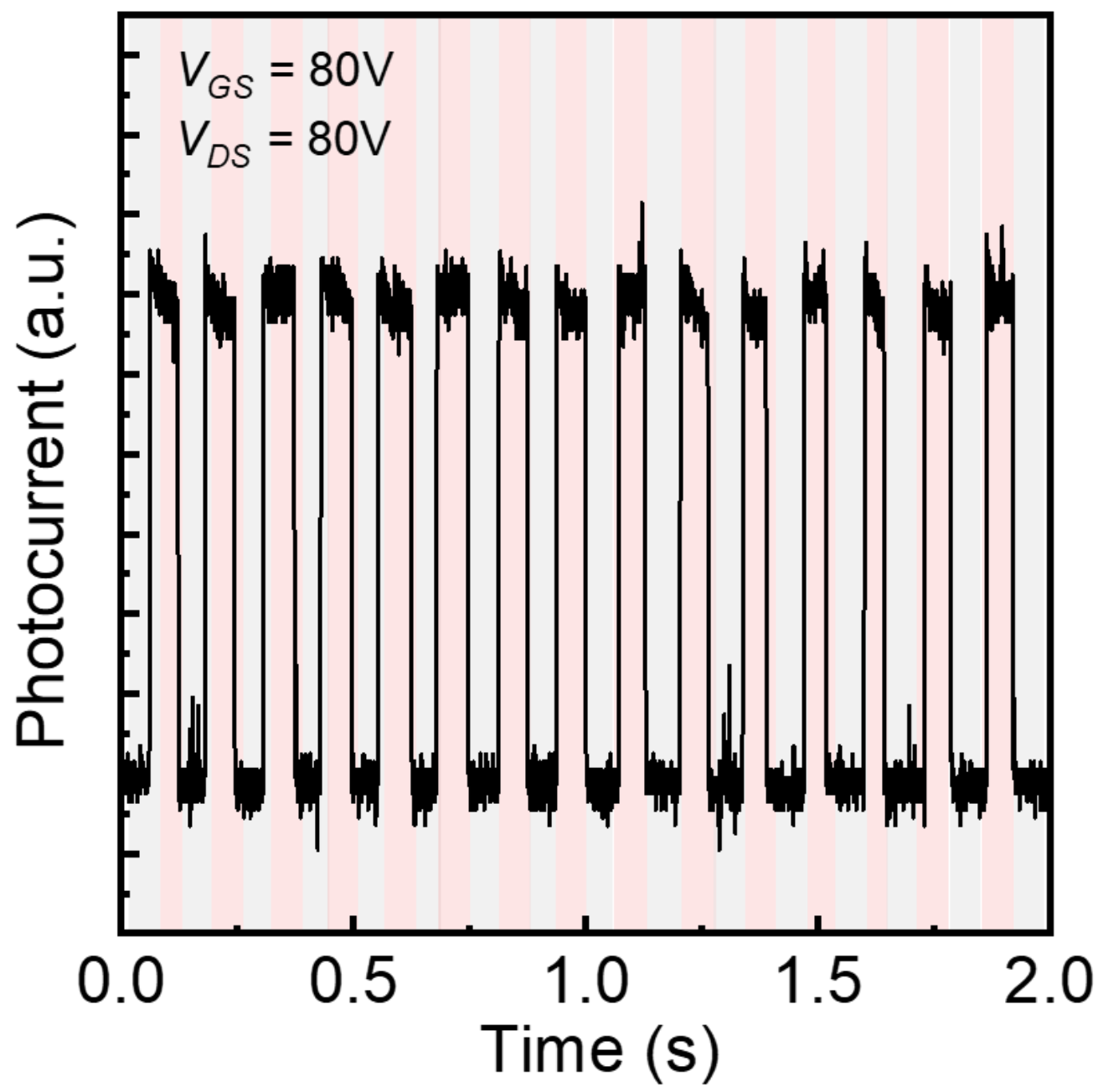

Figure S35. Real-time photoswitching measurements of SB-VOFETs based on IC2F-B(*S*)DMO-IC2F thin films annealed at 230 °C under pulsed illumination (λ = 850 nm).

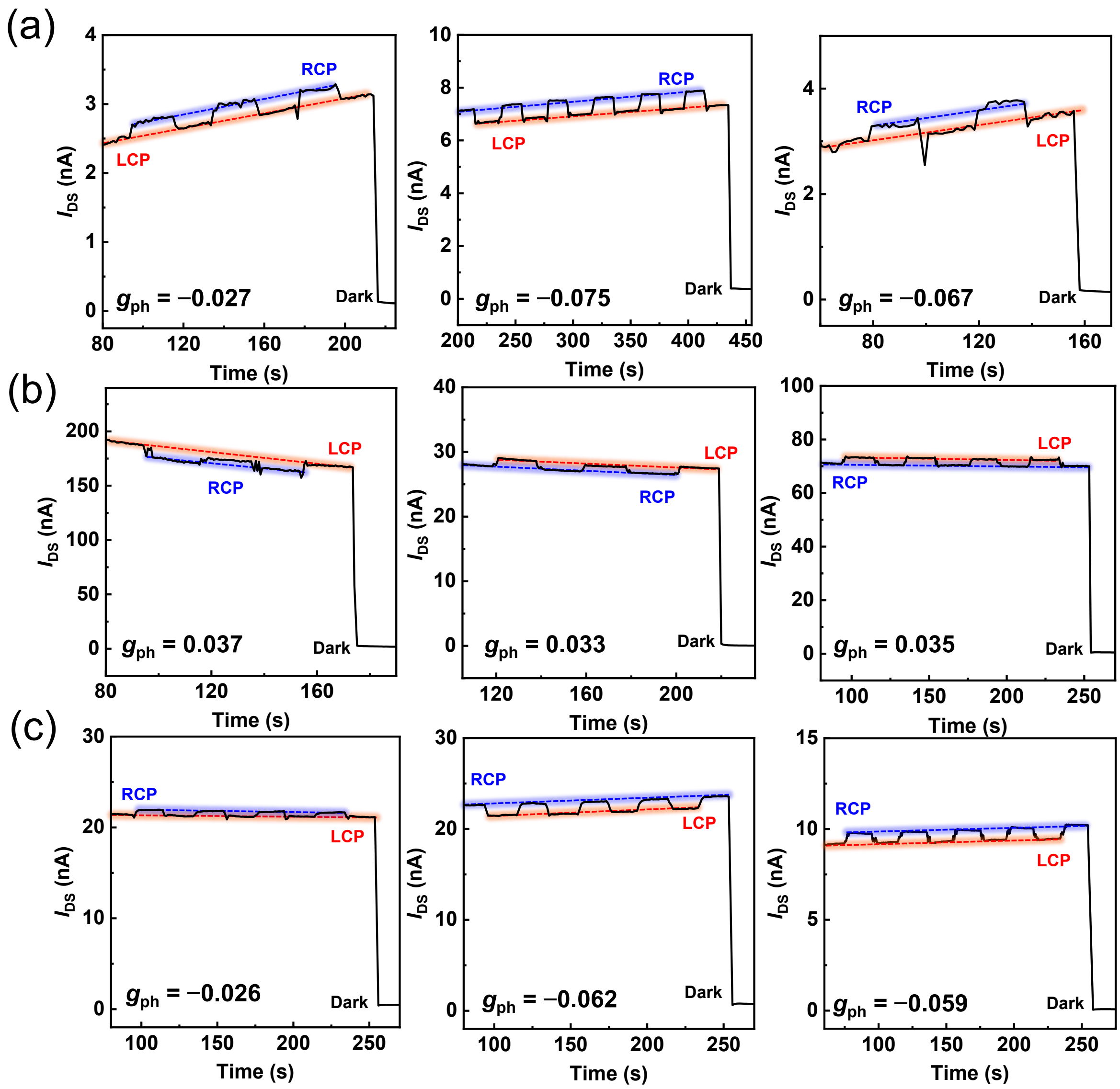

Figure S36. Dynamic photoswitching measurements for extracting g_{ph} values of SB-VOFETs based on (a) IC2F-B(*R*)DMO-IC2F annealed at 100 °C, (b) IC2F-B(*R*)DMO-IC2F annealed at 230 °C, and (c) IC2F-B(*S*)DMO-IC2F annealed at 230 °C.

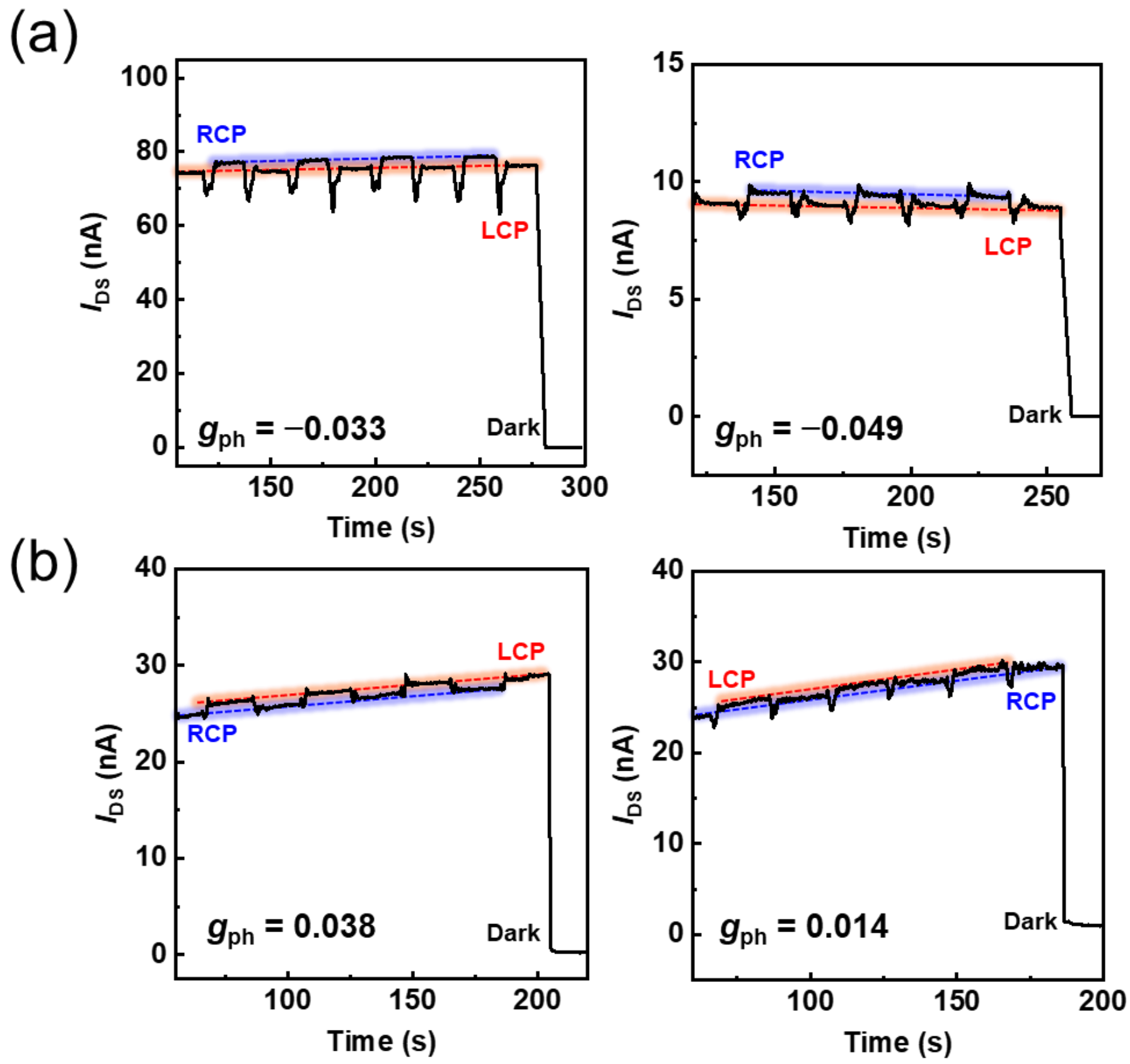

Figure S37. Dynamic photoswitching measurements for extracting g_{ph} values of SB-VOFETs based on (a) IC2Cl-B(*R*)DMO-IC2Cl annealed at 230 °C, and (b) IC2Cl-B(*S*)DMO-IC2Cl annealed at 230 °C.

References

[S1] J. Yuan, Y. Zhang, L. Zhou, G. Zhang, H.-L. Yip, T.-K. Lau, X. Lu, C. Zhu, H. Peng, P. A. Johnson, M. Leclerc, Y. Cao, J. Ulanski, Y. Li, Y. Zou, *Joule* **2019**, 3, 1140.

[S2] S. E. Root, M. A. Alkhadra, D. Rodriquez, A. D. Printz, D. J. Lipomi, *Chem. Mater.* **2017**, *29*, 2646.

[S3] J. W. Shin, D.-W. Kim, D. Kim, D. Moon, *Bull. Korean Chem. Soc.* **2025**, *46*, 594-601.

[S4] Z. Otwinowski and W. Minor, in *Methods in Enzymology,* ed. C. W. Carter, Jr and R. M. Sweet, Academic Press: New York, 1997, vol. 276, part A, p 307-326.

[S5] G. M. Sheldrick, *Acta Cryst.* **2015**, *A71*, 3-8.

[S6] G. M. Sheldrick, *Acta Cryst.* **2015**, *C71*, 3-8.

[S7] Y. Y. Kim, J. Kim, N. Kim, *Zenodo* **2022**, pGIXS: PLS-II 3C SAXS beamline data plot program with MATLAB (0.1 version).
